# Spatially-guided metabolomics profiling of metabolic regions in human tumor tissues

**DOI:** 10.1038/s44320-026-00205-w

**Published:** 2026-04-01

**Authors:** Jia-Ying Joey Lee, Jingtao Zhang, Sin-Chi Chew, Shihleone Loong, Liang Xu, Jia-Wen Carmen Kong, Fedor Grigoryev, Alexander Yaw-Fui Chung, Jin-Yao Teo, Peng-Chung Cheow, Glenn Bonney, Brian K P Goh, Wei-Qiang Leow, Yulan Wang, Lit-Hsin Loo, Pierce Kah-Hoe Chow

**Affiliations:** 1 Bioinformatics Institute, Agency for Science, Technology and Research (A*STAR), Singapore, Singapore; 2 Singapore Phenome Center, Lee Kong Chian School of Medicine, Nanyang Technological University, Singapore, Singapore; 3 Programme in Translational and Clinical Liver Research, National Cancer Centre Singapore, Singapore, Singapore; 4 Department of Anatomical Pathology, Singapore General Hospital, Singapore, Singapore; 5 Department of Hepatopancreatobiliary and Transplant Surgery, Singapore General Hospital and National Cancer Centre Singapore, Singapore, Singapore; 6 Surgery Academic Clinical Programme, Duke-NUS Medical School Singapore, Singapore, Singapore; 7 Division of Hepatobiliary & Pancreatic Surgery, Department of Surgery, University Surgical Cluster, National University Health System, Singapore, Singapore; 8 Human Phenome Institute, Fudan University, Shanghai, China

**Keywords:** Spatial Metabolomics, Mass Spectrometry Imaging, Metabolic Heterogeneity, Hepatocellular Carcinoma, Cancer, Metabolism

## Abstract

Bulk high-resolution mass spectrometry provides sensitive and global snapshots of metabolites involved in cancer metabolism. However, intratumoral heterogeneity obscures the cellular origins of detected metabolites, making it difficult to identify reproducible and predictive metabolic markers. Here, we present “Spatially guided MEtabolomics (SgME) profiling”, a multi-modal metabolomics data analysis approach that delineates and maps metabolic regions (MERs), including overlapping regions, within tumor tissues. We applied SgME profiling to human hepatocellular carcinoma (HCC) tumors and refined potential RNA markers that were also found in previous transcriptomics or bioinformatics studies to those specifically associated with malignant regions. We further estimated that more than 50% of the highly abundant metabolites detected in bulk tumors originated from the non-malignant MERs and are therefore unlikely to be predictive and/or reproducible markers. Importantly, SgME profiling also revealed new potential metabolic markers that were not apparent in bulk analysis because they increased in low-grade tumor regions but declined sharply in necrotic regions. Together, these findings show that SgME profiling overcomes key limitations of conventional metabolomics profiling by enabling more granular, spatially resolved metabolic characterization.

## Synopsis

Spatially-guided MEtabolomics (SgME) profiling is a multi-modal metabolomics data analysis approach to identify reproducible metabolites by integrating the mass separation capabilities of LC-MS/MS and the spatial separation capabilities of mass-spectrometry imaging.

- It deconvolutes heterogenous hepatocellular carcinoma (HCC) tumors into whole-slide maps of overlapping metabolic regions (MERs) associated with different biological and metabolic processes.
- The maps allow the assignment of changed metabolites from bulk metabolomics assays into different MERs and the selection of metabolites spatially associated with tumorigenic MERs and correlated with known potential HCC biomarkers.
- The analysis suggests that more than 50% of the changed metabolites detected in bulk assays may come from non-tumorigenic regions and are therefore unlikely to be predictive and/or reproducible biomarkers of HCC tumorigenesis.

## Abstract (web summary)

Spatially-guided MEtabolomics (SgME) profiling is a multi-modal metabolomics data analysis approach to identify reproducible metabolites by integrating the mass separation capabilities of LC-MS/MS and the spatial separation capabilities of mass-spectrometry imaging.

## Graphical abstract

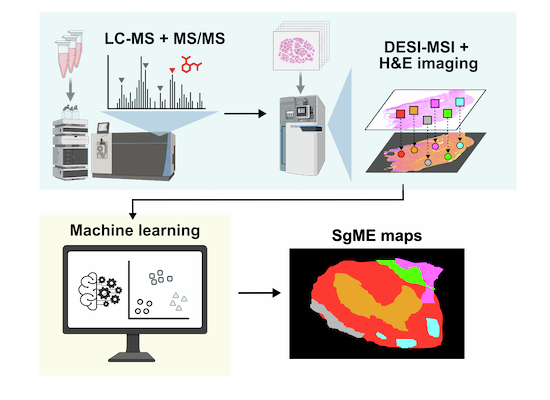

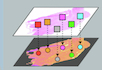

## Introduction

Metabolic reprogramming is a cancer hallmark characterized by changes in the cellular metabolic processes in the tumor microenvironment (TME) that support uncontrolled cancer cell growth and proliferation (Ward and Thompson, 2012; Faubert et al, 2020). Metabolomics profiling using untargeted high-resolution liquid/gas chromatography-mass spectrometry (LC/GC-MS) can provide global and quantitative snapshots of the changes in the intra- and extracellular levels of metabolites consumed or produced by these processes between cancer and normal cells (Han et al, 2020; Sreekumar et al, 2009; Ferrarini et al, 2019) or between different tumor subtypes (Denkert et al, 2012). However, the metabolic environment within a TME is highly heterogeneous due to genetic mutations (Zhai et al, 2017), oxygen and nutrient availability, and the presence of different signaling molecules (Schwörer et al, 2019) or cell types (Kim and DeBerardinis, 2019). Thus, metabolites found in bulk tumors may originate not only from cancer cells but also from other non-cancerous cells, such as normal epithelial, stromal, or immune cells. Furthermore, these metabolites may indicate not only tumorigenesis but also non-tumorigenesis processes, such as steatosis, fibrosis, necrosis, xenobiotic metabolism, or other processes. These intra- and inter-tumoral heterogeneities may lead to the identification of potential metabolic markers that are highly discriminative in specific cohorts, but not generalizable to other cohorts or larger populations (Struys et al, 2010; Jentzmik et al, 2010; Sreekumar et al, 2009).

To better delineate metabolic heterogeneity in tumors, we define a “metabolic region” (MER) as a local cellular niche characterized by the spatial presence of metabolites that are mechanistically associated with key metabolic or biological processes in tumor metabolism or tumorigenesis. This definition is compatible with the concept of “metabolic zonation” in the liver (Kietzmann, 2017; Gebhardt, 1992; Jungermann and Keitzmann, 1996; Porat-Shliom, 2024), in which the spatial compartmentalization of metabolic pathways and functions enables efficient adaptation of liver metabolism to the heterogenous metabolic states and nutritional demands of morphologically similar hepatocytes residing in different local niches. Such zonation spatially separates opposing pathways, thereby preventing competition for shared substrates, and has been described for carbohydrate, amino-acid, lipid, and xenobiotic metabolisms in both parenchymal and non-parenchymal cells (Kietzmann, 2017; Jungermann and Keitzmann, 1996). However, our definition of MER makes explicit a principle that prior definitions of zonation often leave implicit or undefined, namely, the key metabolic or biological processes associated with the metabolites defining a MER may occur in the local, adjacent, or even distant cellular niches. These metabolites may be generated locally or delivered from adjacent or distant regions through transport or diffusion. Therefore, a MER represents a broader and spatially defined tissue domain that may propagate, mediate, or execute the key metabolic or biological processes in tumor metabolism, rather than being restricted to the morphologically or molecularly obvious sites where such processes are most apparent. For example, a metabolically-(ME)-necrotic region may comprise of all tissue regions containing metabolites consumed or produced during cellular necrosis, including histologically non-necrotic regions. As a corollary, this definition allows a single MER to encompass multiple cellular niche types that may have different histological or molecular profiles, while a local cellular niche may be associated with one or more MER types.

The detection and quantification of MERs can be achieved by using mass-spectrometry imaging (MSI) based on desorption electrospray ionization (DESI) (Wiseman et al, 2006) or matrix-assisted laser desorption/ionization (MALDI) (Stoeckli et al, 2001). These analytical technologies can spatially resolve the abundances of metabolites directly on intact tissues, such as esophageal (Sun et al, 2019), breast (Santoro et al, 2020), and gastric (Sun et al, 2023; Wang et al, 2022) tumors. The recent development of air-flow-assisted DESI-MSI (AFADESI-MSI) (He et al, 2011) and MALDI with laser-induced post-ionization MSI (MALDI-2-MSI) (Soltwisch et al, 2015) has further increased the ionization sensitivity and spatial resolutions of MSI.

Despite these advancements in instrumentation, current data analysis approaches for untargeted MSI still have several major limitations that restrict their applications for MER detection. First, altered metabolites were usually identified by comparing different histological regions of interest (ROIs), especially between the tumor and normal tissues (Sun et al, 2019, 2023; Wang et al, 2022; Santoro et al, 2020), or by spatially clustering the detected mass features and computationally dividing the whole MS images into separate tissue regions (Sun et al, 2023; Bemis et al, 2016; Abdelmoula et al, 2016). However, many metabolites may be secreted and/or transported to other tissue regions (Schwörer et al, 2019). Thus, such approaches may miss MERs that spatially encompass multiple types of local cellular niches, each with different histological features, or MERs that spatially overlap on top of each other. Second, the biological processes associated with the identified mass features were usually not elucidated. Ideally, this may be accomplished by performing a spatial transcriptomics assay (Ståhl et al, 2016) and a MSI assay on the same or adjacent tissue sections from the same tumor blocks. However, current tissue storage and processing protocols for freshly frozen tissues for spatial transcriptomic and MSI assays are usually incompatible. Furthermore, most current spatial transcriptomic assays can only capture smaller tissue areas ( < 10 × 10 mm) than MSI assays (whole-slide). Thus, spatial transcriptomic and metabolomic profiling of the same tissue sections remains very challenging. Some recent MSI studies still identified differentially expressed genes based on histopathological regions without using the collected MSI information (Sun et al, 2023). Third, MSI data alone is usually insufficient for metabolite annotation, and MS/MS data is required to fully annotate the detected mass features (Köfeler et al, 2021; Alseekh et al, 2021; Sumner et al, 2007). A recent study found that MALDI-2-MSI alone missed most of the lipids that could be identified by LC tandem MS (LC-MS/MS) (Hendriks et al, 2024). Many current MSI-based studies do not directly generate MS/MS data from MSI, and instead use LC-MS/MS on separate tissue sections to annotate metabolites (Hendriks et al, 2024; Sun et al, 2023; Zhu et al, 2022).

Here, we present “Spatially guided MEtabolomics (SgME) profiling”, a multi-modal and machine-learning-based approach for mapping MERs that integrates spatial (DESI-MSI and hematoxylin and eosin (H&E) imaging) and bulk (untargeted LC-MS/MS and next-generation RNA sequencing (RNA-seq)) profiling of the same or adjacent tissue samples. SgME profiling overcomes the limitations of existing MSI data analysis approaches in deconvoluting the MER compositions of tumor tissues and their associated biological processes. First, SgME profiling uses supervised machine learning to learn the metabolomics profiles of a MER based on multiple small tissue regions selected to exhibit the histological features of one of the key local cellular niches in the MER. Then, the trained model is applied to the whole tissue and automatically identifies all other local cellular niches associated with the MER, some of which may not have the same histology as the initial training cellular niches. Full MER maps that cover the whole tissue are generated. The use of multiple machine-learning models enables overlapping MERs to be defined in these maps. Second, SgME profiling integrates bulk omics profiles from RNA-seq and LC-MS by performing COrrelated MEtabolomics and TranscriptomicS Pathway (COMET’s Path) analysis, which enables the inference of the biological and/or metabolic processes associated with different MERs. Third, SgME profiling combines and leverages the mass separation capabilities of LC-MS/MS and the spatial separation capabilities of MSI to identify and annotate putative metabolites. Our strategy is to start from LC-MS/MS and focus on analyzing highly abundant putative metabolites that can also be found in the MSI data from the same tumors. These metabolites are more likely to be reproducible than low-abundant metabolites. Therefore, SgME profiling can be used to identify, categorize, and interpret MERs from heterogeneous tumor tissues.

Hepatocellular carcinoma (HCC) arises from hepatocytes and is the most common type of primary liver cancer (Sung et al, 2021; Toh et al, 2023). Cholangiocarcinoma (CCA) arises from cholangiocytes and while rare, it represents the second most common type of primary liver cancer (Banales et al, 2016). Intra-hepatic CCA (iCCA) is the most common CCA subtype and presents clinically as mass lesions similar to HCC (Brindley et al, 2021). The absence of robust predictive biomarkers remains central to the challenge of improving clinical outcomes in both HCC and CCA. HCC has high genomic (Zhai et al, 2017, 2022), immunomic (Nguyen et al, 2021), and metabolomic (Berndt et al, 2021) inter- and intra-tumor heterogeneities, which explains why the global collective effort of sequencing bulk HCC tissues to elucidate actionable biomarkers has not been fruitful (Rebouissou and Nault, 2020). Recently, epigenetic changes driven by or giving rise to metabolic changes in the TME have been suggested as major driving factors of dysregulation in HCC (Jeon et al, 2023).

In this study, we used HCC and iCCA tumors as models to evaluate and demonstrate the utility of SgME profiling. We systematically compared the transcriptomics and metabolomics landscapes across different stages of HCC and iCCA and found that early-stage HCC exhibits the greatest intratumoral metabolic heterogeneity. Using SgME profiling, we trained three supervised classifiers based on metabolomic profiles to identify six different MERs in HCC tumors: metabolically (ME)-normal, -low-grade, -high-grade, -necrotic, -fibrotic, and -steatotic regions. Using COMET’s Path analysis, we found many potential HCC biomarkers that had also been reported in previous large-scale transcriptomics or bioinformatics studies, assigned these potential markers to specific MERs, and narrowed them down to those associated specifically with malignant regions. We also estimated that more than 50% of the highly abundant metabolites detected in bulk tumors did not originate from malignant regions and are therefore unlikely to be predictive and/or reproducible biomarkers for HCC. Importantly, we discovered additional potential metabolic markers that were not apparent in bulk analysis because they increased in low-grade tumor regions but sharply decreased in ME-necrotic regions. Our study provides new insights into the metabolic intra-tumor heterogeneity of HCC and may help to guide future metabolomics research.

## Results

### Overview of SgME profiling

**Figure 1:**
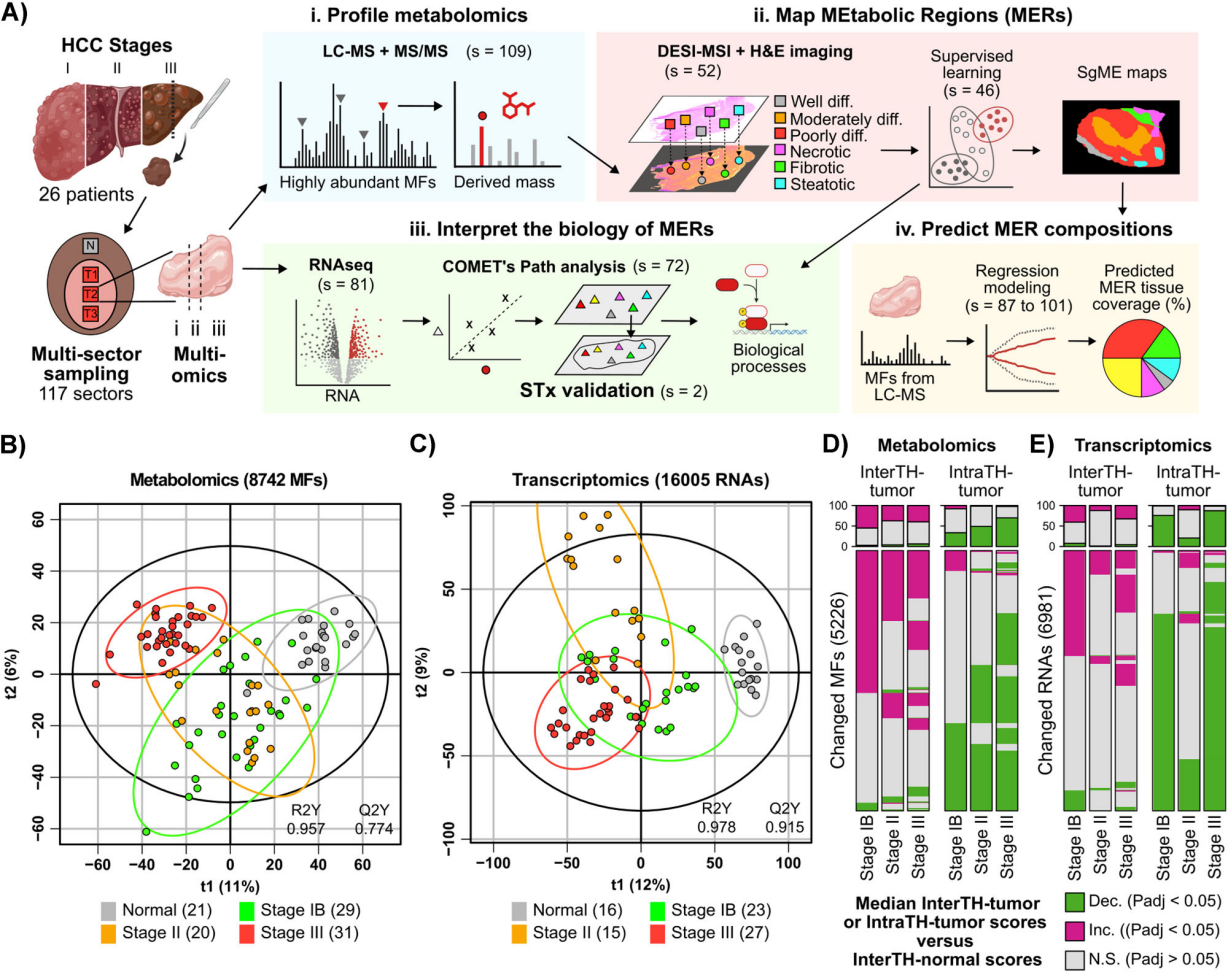
Most disrupted metabolic processes found using bulk profiling are not specific to HCC stages. (**A**) Diagram showing the key steps of Spatially guided Metabolomics (SgME) profiling workflow, including multi-sector sample collection, bulk metabolomics profiling based on LC-MS/MS profiles, metabolic region (MER) mapping based on mass spectrometry and H&E images, biological interpretation based on RNA-seq profiles and COMET’s Path analysis, and MER composition prediction using regression modeling (s = number of tissue sections analyzed using different spatial and/or non-spatial omics profiling methods). PLS-DA score plots showing the (**B**) LC-MS or (**C**) RNA-seq profiles of all the HCC tissue sections mapped to the two most important components (t1 and t2) of the PLS-DA classifiers trained to classify adjacent-normal and tumor sections collected from Stage IB, II, and III HCC patients based on the respective profiles. (Ellipses = Mahalanobis distances, R2Y = explained variances, Q2Y = prediction accuracies of the PLS-DA classifiers.) Stacked bar charts showing the percentages of (**D**) mass features or (**E**) RNAs that had median InterTH-tumor or IntraTH-tumor scores significantly different from median InterTH-normal scores in at least one of the three HCC stages (two-sided Wilcoxon rank-sum test; *P*adj = BH-adjusted *P* values; Inc. = significantly increased, *P*adj <0.05; Dec. = significantly decreased, *P*adj < 0.05; N.S. = not significantly changed, *P*adj > 0.05).

We have developed the SgME profiling approach to generate MER maps for whole tissue sections. In the first stage (Fig. 1Ai), a set of highly abundant mass features is systematically identified from aligned and normalized untargeted LC-MS profiles based on the 90th-percentile and mean abundance levels of all the ions detected across all tumor samples. Putative metabolites are further annotated based on MS/MS fragment patterns and/or manual curations matched with Human Metabolome Database (HMDB) (Wishart et al, 2022) and/or Lipid Maps (Conroy et al, 2024) (“Methods”). Unlike previous methods, we do not directly select mass features by using statistical tests that compare different histological ROIs. In the second stage (Fig. 1Aii), these highly abundant putative metabolites are co-detected from the DESI-MSI images of tissue sections from the same tumors and mapped to multiple MERs using machine-learning methods (“Methods”). To confirm the co-detected peaks are reproducible, the correlations between the normalized bulk LC-MS and averaged DESI-MSI abundance levels of the co-detected peaks are checked across all the tumor samples. SgME maps are then constructed using a set of partial least-squares discriminant analysis (PLS-DA) classifiers (Wold et al, 2001) trained on the abundance levels of these peaks on a set of ROIs annotated by pathologists based on matching H&E images. These classifiers also allow the identification of the most discriminative putative metabolites for each MER. In the third stage (Fig. 1Aiii), the COMET’s Path analysis is used to infer the biology of the MERs. Across all the bulk tumor samples, gene sets with RNA expression levels correlated to the abundance levels of discriminative highly abundant putative metabolites are used to identify enriched biological processes from the Molecular Signatures Database (MSigDB) (Liberzon et al, 2015) (“Methods”). Optionally, the spatial distributions of these genes may also be confirmed using spatial transcriptomic profiling. In the fourth stage (Fig. 1Aiv), the generated SgME maps are used to quantify MER compositions, identify altered putative metabolites across different MERs, and predict MER compositions of bulk tumor samples.

### Different liver cancer types have altered metabolomics landscapes

To evaluate SgME profiling, we studied 117 tumor and matched adjacent-normal tissues from 26 surgically resected primary liver tumors from the Precision Medicine in Liver Cancer across an Asia-Pacific Network (PLANet) cohort (Zhai et al, 2022) (Fig. 1A; Appendix Fig. S1a,b). Twenty-four of the tumors were histologically confirmed as HCC, and two of them were iCCA (Appendix Table S1). To allow quantification of intra-tumor heterogeneity, multiple sectors were harvested per tumor depending on the tumor size (Zhai et al, 2017) (Appendix Table S2). We found that the LC-MS profiles (Appendix Fig. S2) of HCC tumors (*s* = 80), iCCA tumors (*s* = 6), and adjacent-normal tissues (*s* = 21) could be almost perfectly separated (Q^2^Y = 0.839, Appendix Fig. S3). Thus, HCC and iCCA may differentially disrupt the metabolic processes in the liver. Although this was not the main focus of our current study, the results suggest that HCC and iCCA may be distinguished based on metabolic biomarkers. Interestingly, we also found that the adjacent-normal liver tissues from both cancer types have very similar metabolomics profiles. Thus, in the subsequent LC-MS analyses, we studied the tumor sections only from the HCC tumors, but the adjacent-normal tissue sections from both the HCC and iCCA tumors. The inclusion of adjacent-normal sections from the iCCA tumors increased the statistical power of our subsequent analyses.

### Most disrupted metabolic processes found using bulk profiling are not stage-specific

Can we identify stage-specific metabolic changes in HCC? Using PLS-DA (Wold et al, 2001), we classified all the tissue sections according to their HCC stages and found progressive alternations in the metabolomics landscapes from normal tissue to Stage III tumor sections (Fig. 1B). Interestingly, the metabolomics profiles of Stage IB barely overlapped with Stage III, while Stage II overlapped with the other two stages (Fig. 1B). Most (>70%) of the mass features changed monotonically with cancer stage (Appendix Fig. S4a). We also analyzed 81 tissue sections using RNA-seq and found that Stage IB also had different transcriptomics profiles than Stage III (Fig. 1C). However, unlike the metabolomics profiles, Stage II had very different transcriptomics profiles and higher variability than the other two stages (yellow ellipse in Fig. 1C). Most (>60%) of the RNAs also changed monotonically with cancer stage (Appendix Fig. S4b). Together, our results suggest that different metabolic processes were disrupted in early- and late-stage HCC tumors, and metabolomics changes are more distinctive between Stage IB and III HCC tumors than transcriptomics changes. Thus, metabolites specific to early-stage HCC may be identified and used as biomarkers for HCC. However, metabolomics variability (colored ellipses in Fig. 1B) was larger in Stage IB than in Stage III. Identifying these early-stage metabolites may be challenging due to the mixed tissue types in the bulk metabolic profiles.

**Figure EV1:**
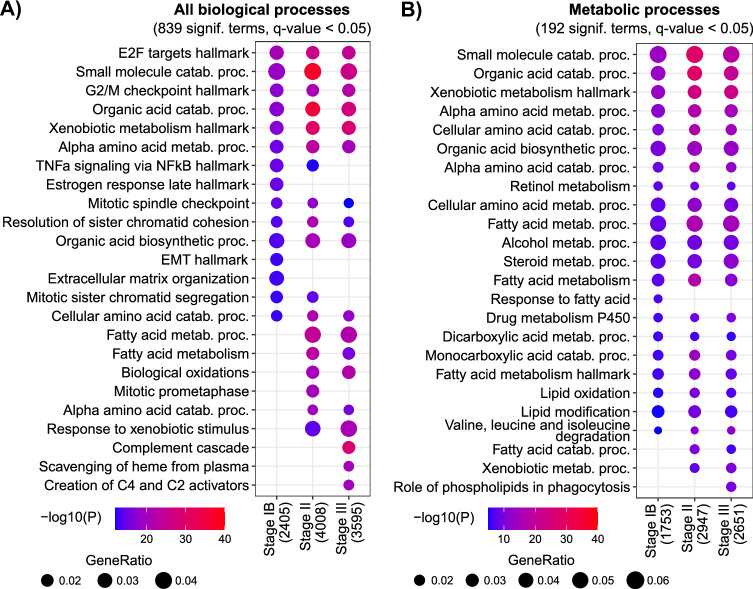
Biological and metabolic processes that changed at different HCC stages. Dot plots showing the top significantly enriched or de-enriched (**A**) biological processes or (**B**) metabolic processes in the differentially expressed genes between adjacent-normal tissues and Stage IB, II, or III tumor sections (two-sided hypergeometric tests; *q* value = expected positive false discovery rate obtained from the Storey’s procedure (Storey, 2002); numbers after the *x* axis labels = the numbers of found differentially expressed genes).

To interpret the observed metabolomics changes, we identified differentially expressed genes between HCC tumor and adjacent-normal sections (*P*_adj_ <0.05 and |log2FC | >0.263; “Methods”), and found that 839 of the 9509 biological processes in the MSigDB database (Liberzon et al, 2015) were significantly enriched/de-enriched (*q* value < 0.05). Our results show that cell division and catabolic processes are consistently perturbed across the three HCC stages, but cell signaling and stromal remodeling start to occur at Stage IB, followed by lipid metabolism and DNA replication at Stage II, and immune activation at Stage III (Fig. EV1A; Appendix Fig. S5a).

To have a more specific analysis of cancer metabolism, we focused on a subset of 1719 metabolism-related processes and found that 192 of them were significantly enriched/de-enriched (*q* value < 0.05). Most of them (such as metabolisms of organic acids, retinols, amino acids, steroids, or fatty acids) are commonly enriched/de-enriched across all or two of the stages (Fig. EV1B; Appendix Fig. S5b). These results agree with the gradual changes of global metabolomics landscapes observed across the three HCC stages (Fig. 1C). Most disrupted metabolic processes found by bulk tissue profiling are not specific to HCC stages.

### Early-stage HCC has maximum intratumoral metabolic heterogeneity

HCC tumors may comprise multiple cellular subpopulations with different or even distinct metabolic states or functions. We hypothesize that the observed gradual metabolic changes may be due to mix-ups of these subpopulations during the bulk metabolomics and transcriptomics analyses. To test the hypothesis, we quantify inter- and intra-tumor heterogeneity scores at the global metabolomics and transcriptomics levels using the multi-sector samples from the same tumors (Fig. 1A and “Methods”). For inter-tumor heterogeneity scores, we found that both metabolomics and transcriptomics profiles had the highest values at Stage IB (Fig. 1D,E). However, for intra-tumor heterogeneity scores, metabolomics and transcriptomics profiles had the highest values at Stages IB and II, respectively (Fig. 1D,E). The transcriptomics results agree with our previous observations that mid-stage HCC tumors have the highest transcriptomic intra-tumor heterogeneity (Zhai et al, 2022). Our current results further suggest that early-stage HCC tumors are more heterogeneous metabolically than transcriptionally. This may indicate the existence of multiple regions or niches with different metabolic properties within the same tumor microenvironments.

### LC-MS and DESI-MSI abundance levels of highly abundant metabolites are positively correlated

**Figure 2:**
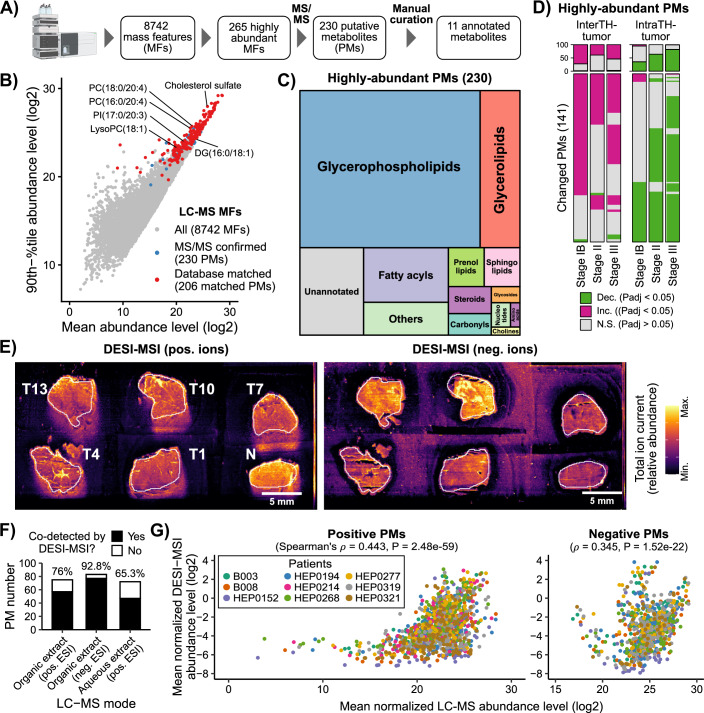
DESI-MSI can detect and spatially resolve the tissue abundance levels of most of the highly abundant metabolites found using LC-MS. (**A**) Diagram showing the key steps in identifying a set of highly abundant putative metabolites (PMs) and a subset of annotated metabolites from the LC-MS profiles of HCC tumor samples. (**B**) Scatter plot showing the 90th-percentile and mean abundance levels for all the 8742 mass features detected by LC-MS and the 230 PMs confirmed by MS/MS across all the HCC tissue sections. (**C**) Tree plot showing the distribution of the key chemical families of the 230 highly abundant PMs. (**D**) Stacked bar charts showing the percentages of highly abundant PMs that had median InterTH-tumor or IntraTH-tumor scores significantly different from median InterTH-normal scores in at least one HCC stage (*P*adj = BH-adjusted *P* values, Inc. = significantly increased, Dec. = significantly decreased; N.S. = not significantly changed. Wilcoxon rank-sum tests). (**E**) DESI-MSI images for patient HEP0152 showing the total ion currents for all the detected positive (left) or negative (right) ions (white lines = annotated tissue section boundaries). (**F**) Bar charts showing the numbers of highly abundant PMs co-detected by LC-MS and DESI-MSI. (**G**) Scatter plots showing the correlations between mean normalized DESI-MSI versus LC-MS abundance levels for all the co-detected positive (left) or negative (right) highly abundant PMs (*ρ =* Spearman’s rank correlation coefficients). Source data are available online for this figure.

To detect reproducible metabolites, we focused on a subset of 265 highly abundant ions detected from all the tissue sections (Fig. 2A,B; Appendix Table S3). We putatively confirmed 230 of them using MS/MS and matched 206 of them with metabolites in HMDB (Wishart et al, 2022) and/or Lipid Maps (Conroy et al, 2024) (“Methods”). The three largest chemical families found are glycerophospholipids, glycerolipids, and fatty acyls (Fig. 2C). Among them, 141 putative metabolites were found to be significantly altered in either Stage IB, II, and/or III tumors compared to adjacent normal tissues (*P*adj <0.05, |log2FC | >0.26; Appendix Fig. S4c). Some of these highly abundant putative metabolites include phosphatidylcholine (PC) (18:0/20:4), PC(20:4/16:0), cholesterol sulfate, and others (Fig. 2B). We also found that the inter- and intra-tumor heterogeneity scores of these putative metabolites were very similar to the global metabolomics trends (Fig. 2D). We still kept those putative metabolites with insignificant changes, which may be due to intra-tumor heterogeneity.

**Figure EV2:**
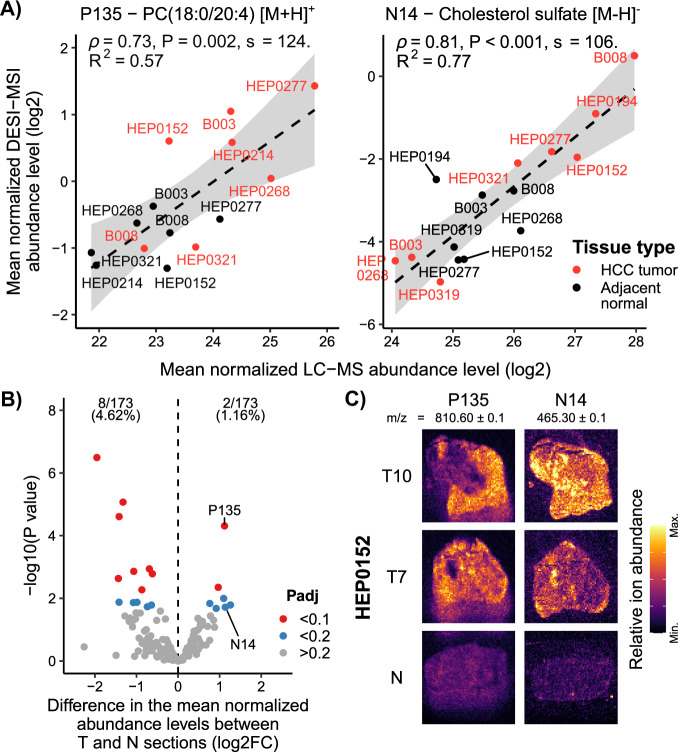
Highly abundant putative metabolites that changed significantly between matched tumor and adjacent-normal tissue sections. (**A**) Scatter plots showing the correlations between mean normalized DESI-MSI versus LC-MS abundance levels for PC(18:0/20:4) (left) or cholesterol sulfate (right) (*ρ =* Spearman’s rank correlation coefficients, *P* = *P* values of the Spearman’s rank correlation coefficient values). (**B**) Volcano plot showing highly abundant putative metabolites with significantly changed averaged tissue abundance levels between the adjacent normal tissue and tumor sections (two-sided *t* test, *P*adj = BH-adjusted *P* values). (**C**) Exemplary DESI-MSI images of PC(18:0/20:4) (left) and cholesterol sulfate (right) ions on three selected tissue sections from patient HEP0152 (N = adjacent normal tissue sections, T7 and T10 = HCC tumor sections).

We performed DESI-MSI (Fig. 2E) followed by H&E imaging on 52 tissue sections from 11 patients (Appendix Fig. S1c). The mass values of 78.7% of the MS/MS-confirmed and highly abundant putative metabolites were successfully matched to the DESI images (Fig. 2F). If these matched peaks correspond to the same metabolites, we expect their bulk LC-MS and averaged DESI-MSI abundance levels to be positively correlated to each other across all the tissue sections. Indeed, we found significant positive correlations without obvious patient- or batch-dependent effects (Spearman’s *ρ* = 0.443 or 0.345 for positive or negative ions, respectively; *P *< 0.001; Fig. 2G). The positive correlations were also observed at the individual metabolite levels (Fig. EV2A), suggesting we were able to obtain highly reproducible DESI-MSI measurements.

To identify metabolites that may distinguish between tumor and normal tissues, we traced the boundaries of all the sections based on the total ion current (TIC) images (Fig. 2E and “Methods”) and measured the mean normalized abundance levels of all the highly abundant putative metabolites on each section. We found 173 co-detected highly abundant putative metabolites in at least two adjacent-normal and two HCC tumor sections from different patients, but only two (1.16%) or eight (4.62%) of them were significantly increased or decreased, respectively (*P*adj <0.10, two-sided *t* test; Fig. EV2B). For example, PC(18:0/20:4) and cholesterol sulfate were among the highly abundant putative metabolites with the largest increases in tumor sections, which were also visually confirmed from their DESI images (Fig. EV2C). PC(18:0/20:4) contains stearic acid (18:0) and arachidonic acid (20:4) at the sn-1 and sn-2 positions. PC and phosphatidylethanolamine (PE) are the two most abundant phospholipids in the plasma membranes of all mammalian cell types, and changes in the molar ratio between PC and PE have been linked to metabolic dysfunction-associated steatotic liver disease (MASLD) in animal models and humans (van der Veen et al, 2017). Cholesterol sulfate is synthesized via the sulfonation of cholesterol by the cholesterol sulfotransferase SULT2B1b and can be found in many types of biofluids and tissues, including liver (Strott and Higashi, 2003). Elevated levels of plasma cholesterol sulfate were found in patients with liver cirrhosis and hypercholesterolemia (Tamasawa et al, 1993). Despite getting some interesting “hits” at this stage, the overall number of putative metabolites that were found to be significantly changed at the tissue-averaged level was very low. This is likely due to the high inter- and intra-tumor heterogeneities in HCC tumors (Fig. 2D). Again, we did not select or discard any potential HCC biomarker solely based on these tissue-averaged results because some of them may change at specific MERs.

### Key histological features of HCC have different metabolomics profiles

**Figure 3:**
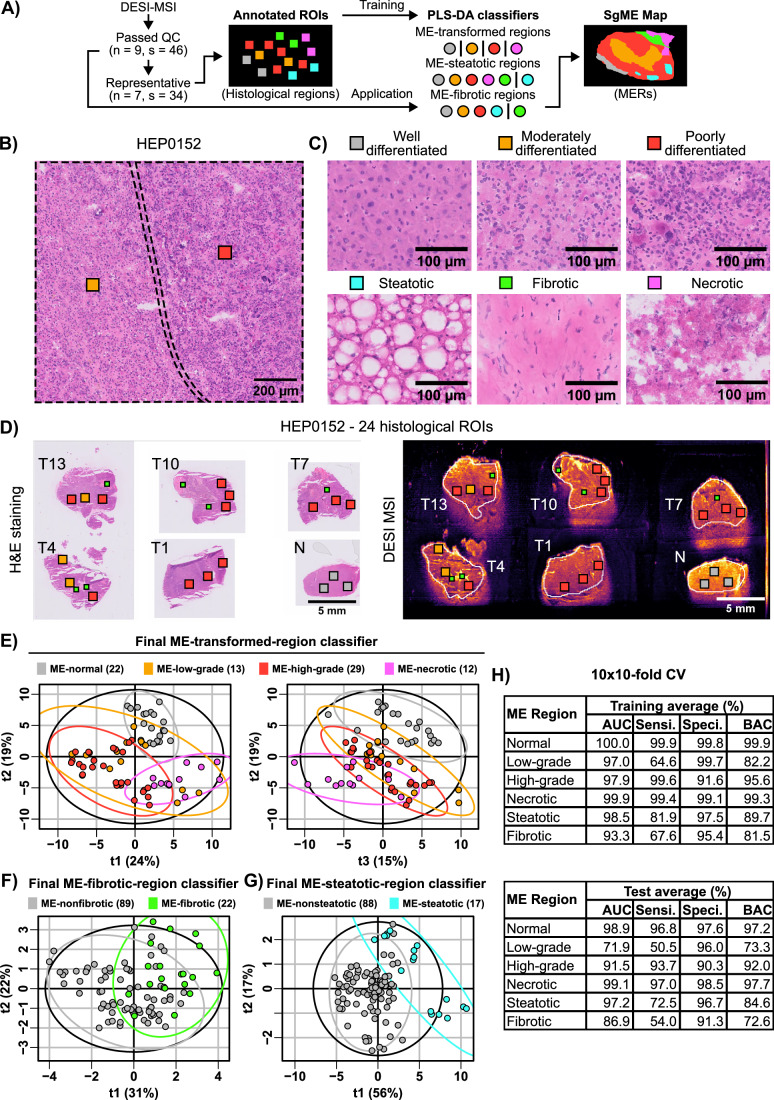
Key histological features of HCC are associated with MERs with different metabolomics profiles. (**A**) Diagram showing the key steps in constructing SgME maps based on the annotated ROIs using three different PLS-DA classifiers (squares = annotated histological ROIs on H&E and DESI-MSI images, circles = metabolomics profiles of the ROIs from DESI-MSI images). Exemplary H&E image from patient HEP0152 showing (**B**) heterogeneous histological features within the same HCC tumor (dashed lines = annotated histological regions; orange or red regions = moderately or poorly differentiated hepatocytes, respectively), and (**C**) the six key histological features annotated in our study. (**D**) Exemplary H&E and DESI-MSI images from the same patient showing the locations of the annotated ROIs that clearly exhibit one of the six key histological features (refer to “Methods” for the ROI annotation procedures). The MSI image shown is the same MSI image shown in Fig. 2E for the sample. (**E**–**G**) PLS-DA score plots showing the highly abundant putative metabolite profiles of all the annotated ROIs mapped to the two or three most important components (t1, t2, and t3) of the three PLS-DA classifiers trained to classify the indicated MER types (ellipses =  Mahalanobis distances.) (**H**) Average training and test performances of the three final classifiers estimated using a 10 × tenfold cross-validation (CV) procedure (BAC = balanced accuracy). Source data are available online for this figure.

Due to the high inter-tumor heterogeneity, training classifiers based on tumor cases without matching adjacent normal tissues may lead to overfitted classifiers that pick up individual-specific changes. Thus, we required all the training cases to have matching adjacent-normal and tumor sections from the same patients, and all the sections to be representative of their expected pathological types. The H&E images were examined and annotated by a trained pathologist and cross-checked by another trained pathologist (“Methods”). The adjacent “normal” section from patient HEP0194 was found to have very few normal hepatocytes, and the tumor sections from patient HEP0319 had abnormally high necrotic regions. Therefore, DESI images from these two patients were excluded from classifier training, but the final classifiers were still being applied to them to construct SgME maps (Fig. 3A; Appendix Table S2).

**Figure EV3:**
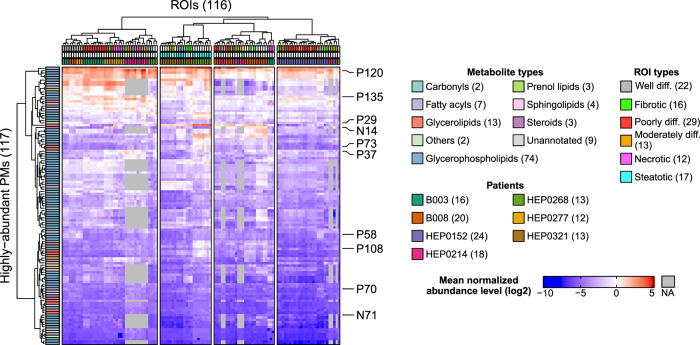
The abundance levels of highly abundant putative metabolites on DESI-MSI ROIs. Heatmap showing the mean normalized abundance levels of the 117 highly abundant putative metabolites that could be detected in more than 50% of these ROIs on the DESI-MSI images. The rows and columns were clustered using hierarchical clustering and Ward’s linkage. Selected putative metabolites of interest were highlighted [P29 = laudanosine, P37 = oleoylcarnitine, P58 = lysoPC(18:1), P70 = DG(16:0/18:3), P73 = DG(16:0/18:1), P108 = PC(15:0/18:0), P120 = PC(16:0/20:4), P135 = PC(18:0/20:4), N14 = cholesterol sulfate, and N71 = PI(17:0/20:3)] (NA = data not available because negative DESI-MSI ions were not collected for HEP0214).

The examination of the H&E images further revealed that most tumor sections were heterogeneous (Fig. 3B; Appendix Fig. S6) and composed of six histological features: well-, moderately-, or poorly differentiated, fibrotic, steatotic, or necrotic regions (Fig. 3C). For example, the formation of large lipid droplets is a characteristic of the steatotic regions, and fibrous scar tissues and collagens are characteristics of the fibrotic regions. Based on these features, a total of 116 ROIs were annotated from the H&E images and spatially mapped to the matching DESI images (Fig. 3D). We detected 117 highly abundant putative metabolites in >50% of these ROIs and computed their mean abundance levels for each ROI (Fig. EV3).

Three PLS-DA classifiers were trained based on the mean ROI abundance levels of these highly abundant putative metabolites (“Methods”). The first classified each pixel into one of the four MER types, namely ME-normal, -low-grade, -high-grade, or -necrotic regions (Fig. 3E). The second classified each pixel into either ME-fibrotic or non-ME-fibrotic regions (Fig. 3F); and the third classified each pixel into either ME-steatotic or non-ME-steatotic regions (Fig. 3G). The last two classifiers allow the predicted MERs to overlap with other MERs. We found that the classifiers achieved 81.5–99.9% balanced training accuracies and 72.6–97.7% balanced test accuracies (Fig. 3H). The ME-normal, -high-grade, and -necrotic regions had the highest test accuracies (>90%), while ME-low-grade and -fibrotic regions had the lowest test accuracies (>70%). We also performed similar analyses using support vector machines (SVMs) with linear and radial-basis-function kernels and found that PLS-DA classifiers outperformed them (Appendix Fig. S7). Our results show that the six HCC histological features are spatially associated with different metabolomics signatures.

### MERs in HCC are defined by different discriminative metabolites

**Figure 4:**
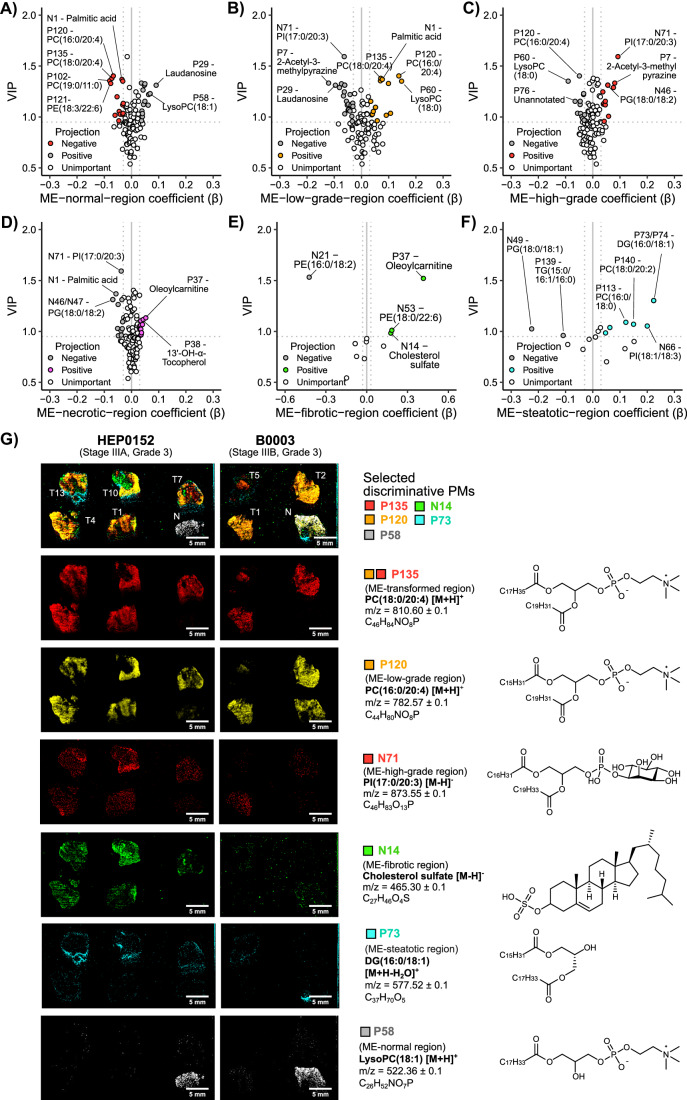
MERs in HCC are defined by different sets of putative metabolites. (**A**–**F**) Volcano plots showing the variable influence on projection (VIP) (Wold et al, 2001) values versus the PLS-DA model coefficients (*β*) for each of the indicated MERs (negative = *β* < −0.03 and VIP > 0.95, positive = *β* > −0.03 and VIP > 0.95, unimportant = |*β* | < 0.03 or VIP < 0.95). (**G**) Exemplary DESI-MSI images showing the spatial abundance levels of selected most discriminative putative metabolites (with the highest VIP and/or |*β*| values) on HCC tumor and adjacent normal tissue sections. The chemical structures shown were annotated based on the MS/MS spectra of the ions (Appendix Fig. S8). Source data are available online for this figure.

**Figure EV4:**
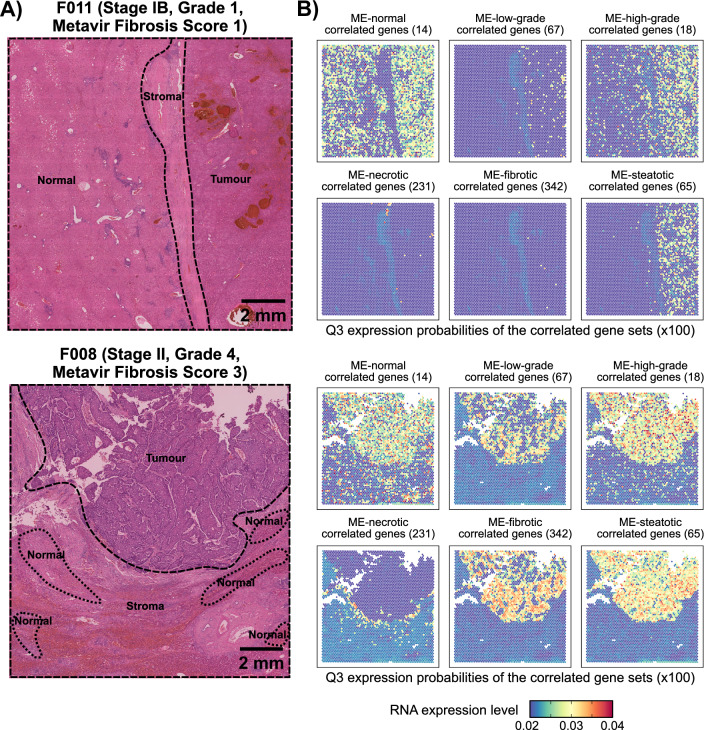
Spatial RNA expression patterns of gene sets significantly correlated with discriminative metabolites from each MER. (**A**) H&E images showing the tissue areas captured by the spatial transcriptomics slides for HCC tumors from patients F008 and F011. (**B**) Heatmaps showing the 75th percentiles (Q3) of the expression probabilities of all the positively correlated gene sets (numbers after the correlated gene-set labels = the numbers of genes from the correlated gene sets also detected by the spatial transcriptomics assays).

We identified the most discriminative putative metabolites for each MER classifier by extracting the VIP scores (Wold et al, 2001) and β coefficients (Mehmood et al, 2012) of the final classifiers. The sign of β indicates if the putative metabolite is positively or negatively associated with a specific MER. By using these two criteria (VIP > 0.95 and *β* > 0.03), we identified 3 to 16 discriminative and increasing highly abundant putative metabolites for each of the six MER types (Fig. 4A–F); except for the ME-normal-region, we also identified putative metabolites with *β* < −0.03, which have higher abundances in the non-ME-normal or “ME-transformed” regions (Fig. 4A). We selected 11 of them (mostly with the largest VIP or |*β*| values) and manually verified their annotations based on MSMS fragmentation spectra (Appendix Fig. S8). They include cholesterol sulfate, diacylglycerol (DG) (16:0/18:1), glycolithocholate acetate, laudanosine, lysophosphatidylcholine (lysoPC(18:1/0:0)), lysoPC(18:0/0:0), oleoylcarnitine, PC(16:0/20:4), phosphatidylinositol (PI(17:0/20:3)), PC(18:0/20:4), and 13’-OH-α-tocopherol (Appedix Table S3). We also confirmed their expected tissue localization patterns based on the DESI-MSI images (Fig. 4G). For example, PC(18:0/20:4) was found to be more abundant in the tumor than in the adjacent-normal sections (Figs. EV2B and 4G). Some of them, such as oleoylcarnitine, were found to be associated to more than one MER type (Fig. 4D,E). Surprisingly, we also found some non-endogenous metabolites, such as laudanosine, a metabolite of the neuromuscular-blocking drugs, atracurium and cisatracurium (Fodale and Santamaria, 2002), which were typically used as muscle relaxants during the liver surgeries of our patients. These non-endogenous metabolites are “discriminative” in our study, may be because tumor tissues have different xenobiotic metabolism capabilities than normal tissues (Fig. EV1). However, these metabolites are not related to tumorigenesis and thus should not be used as HCC biomarkers. The results show the high sensitivity of mass spectrometry imaging and also the importance of accurate metabolite annotations during spatial metabolomics profiling of human tumor tissues. Overall, our results support the hypothesis that HCC tissues are metabolically heterogeneous and can be spatially divided into multiple MERs. Each of these regions is defined by multiple highly abundant putative metabolites. We did not observe a single dominant putative metabolite in any of the classifiers, suggesting that single markers are insufficient to recognize these MERs.

### These metabolites are correlated to RNAs involved in different biological processes

**Figure 5:**
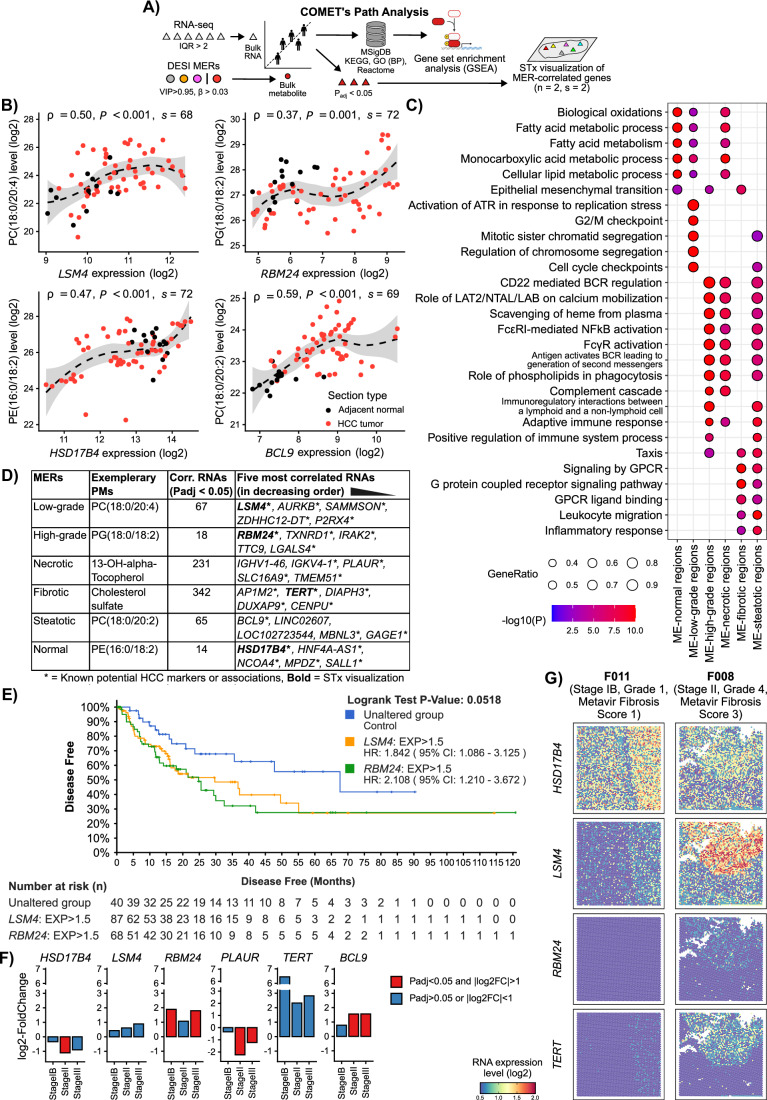
MERs in HCC are correlated with specific biological processes and metabolic enzymes. (**A**) Diagram showing the key steps of COMET’s Path analysis. (**B**) Scatter plots showing the relationships between the four top correlated genes and selected discriminative metabolites that are positively correlated with them (ρ = Spearman’s rank correlation coefficient, *s* = number of tissue sections). (**C**) Dot plots showing the top-most significantly enriched biological processes found using Gene Set Enrichment Analysis (GSEA) in all the genes ranked according to their correlations to the discriminative putative metabolites in every MER (*P*adj = BH-adjusted *P* values, *P*adj <0.05, nonparametric permutation test in GSEA; BCR = B-cell receptor). (**D**) The list of top five genes with the highest positive correlations and the total number of genes with significant positive correlations (*P*adj <0.05, Spearman’s rank correlation test) to the discriminative putative metabolites for each MERs. (**E**) Disease-free survival curves obtained from cBioPortal on a HCC dataset from the TCGA PanCancer Atlas (HR = hazard ratio, EXP = mRNA expression z-scores relative to normal samples). (**F**) Bar charts showing the changes in the bulk RNA expression levels of the top correlated genes in different HCC stages (log2FC = log2-fold change of expression levels between tumor and normal sections, *P*adj = BH-adjusted *P* values, two-sided student’s *t* test between tumor and normal sections). (**G**) Heatmaps showing the spatial RNA expression patterns of four top correlated genes in tumor samples from two HCC patients. Source data are available online for this figure.

The inference of the biological processes or metabolic pathways involving these discriminative putative metabolites is very challenging due to the difficulties in performing both spatial transcriptomics and metabolomics assays on the same or adjacent tissue sections (see “Introduction”). Instead, we developed a COrrelated MEtabolomics and TranscriptomicS Pathway (COMET’s Path) analysis method (Fig. 5A and “Methods”) to computationally infer the RNAs expressed in each MER by correlating bulk RNA-seq and LC/MS profiles. The method assumes that RNAs and metabolites with correlated abundance levels across all the collected tissue sections are likely to localize in the same MERs and be involved in the same or related biological or metabolic processes. We focused on analyzing only discriminative putative metabolites that contribute positively to the SgME classifiers (i.e., those with VIP > 0.95 and *β* > 0.03).

We ranked all the changed RNAs from the bulk RNA-seq experiments according to their mean absolute rank correlations to the discriminative putative metabolites from each of the six MERs (Fig. 5B). Then, we applied Gene Set Enrichment Analysis (GSEA) (Subramanian et al, 2005) to the ranked gene lists and found 58–333 significantly enriched biological processes for the six MERs (Fig. 5C; Appendix Fig. S9). Many of these processes, such as fatty acid and lipid metabolic processes or G2/M and cell cycle checkpoints, were also found to be commonly enriched in the differentially expressed genes from all HCC stages under bulk RNA-seq analysis (Fig. EV1). However, COMET’s Path analysis allowed us to associate these processes with more specific MERs that are not obvious from the bulk analysis. Specifically, we found that fatty acid and lipid metabolic processes were enriched in the ME-normal, -low-grade, and -necrotic regions, but surprisingly not in the ME-high-grade region (Fig. 5C). Similarly, key tumorigenesis processes, such as G2/M and cell cycle checkpoints, were enriched only in ME-low-grade regions but not any other MERs.

Importantly, we also found that most of these biological processes agree with our definitions of MERs. For example, discriminative putative metabolites of the ME-normal regions are correlated with genes involved in core metabolic functions of normal livers, such as lipid and fatty acid metabolism, catabolic, and biosynthetic processes. We also found that putative metabolites of the ME-low-grade and -steatotic regions are correlated with RNAs involved in cell cycles, divisions, and proliferation, which are the characteristics of hepatocytes during tumorigenesis or steatosis (Hall et al, 2021). Unexpectedly, we found that the metabolites in the ME-high-grade regions are quite different from those in the ME-low-grade regions and correlated with RNAs involved in adaptive immune responses, including activations of B-cell receptors (BCR), high-affinity IgE receptors (FcεRI), and the complement systems. Our results suggest that in ME-high-grade regions, the transformed hepatocytes may slow down their proliferation and metabolism functions, while B cells may produce antibodies that form immune complexes with tumor antigens, activate the complement pathways, and drive chronic inflammation, angiogenesis, and T-cell suppression in HCC (Xiao et al, 2022). Interestingly, these pathways were also enriched in the ME-necrotic and -steatotic regions, suggesting these three MERs may overlap or be close to each other in the tumor tissues. Finally, we found that leukocyte activation and migration and vasculature development were enriched only in the ME-fibrotic and -steatotic regions, agreeing with known roles of T-cell and other lymphocytes and angiogenesis in liver fibrosis and fatty livers (Hammerich and Tacke, 2023; Peiseler et al, 2022).

### COMET’s Path assigns potential HCC biomarkers to specific MERs and metabolites

To further investigate and interpret the biological processes that may drive the discriminative metabolites in different MERs, we determined the significantly correlated genes for each of the six MERs (*P*_adj_ <0.05, Spearman’s rank correlation test; Fig. 5D). Interestingly, we found that almost all of the top-most significantly correlated genes have been previously found to be potential HCC markers or associated with HCC (Fig. 5E). Many of them are differentially expressed between tumor and adjacent-normal regions in our cohort (Fig. 5F). For some of these potential HCC markers, their elucidated functions or mechanisms agree with COMET’s Path’s assignment of metabolic regions. For example, telomerase reverse transcriptase (*TERT*) promoter mutation is an early somatic genetic alteration associated with the transformation of cirrhosis (advanced stage of liver fibrosis) to HCC (Nault et al, 2014; Müller et al, 2020). COMET’s Path correctly assigned the gene to the ME-fibrotic region. Another example is B-cell CLL/lymphoma 9 (BCL9), which activates Wnt/β-catenin signaling pathways (Gay et al, 2019) that modulate lipid formation in MASLD and HCC (Wang et al, 2023). COMET’s Path also correctly assigned the gene to the ME-steatotic region.

Importantly, many of these top-correlated genes were discovered as potential HCC markers via large-scale bioinformatics analyses, and most of them have unknown, unclear, or even conflicting previous reports on their functions or roles in HCC. By using SgME profiling and COMET’s Path analysis, we were able to assign them to specific metabolic regions or metabolites and hypothesize their potential roles in HCC. The first example is the Like-Smith (LSM) protein family, a group of RNA-binding proteins involved in RNA metabolism. In a previous integrative bioinformatics analysis based on multiple clinical databases, *LSM4* was found to be overexpressed in HCC and a strong diagnostic marker (AUC ~ 0.9) (Chen et al, 2022). COMET’s Path found that *LSM4* is the most correlated RNA with the ME-low-grade region, including PC(18:0/20:4) (Fig. 5B), and thus the gene is likely to be involved in the tumorigenesis process and a strong HCC marker candidate. The second example is the urokinase-type plasminogen activator receptor (uPAR) encoded by *PLAUR*, which was found to be a potential HCC invasiveness and prognosis marker (Zheng et al, 2000), but the precise roles of the receptor in HCC are unclear. COMET’s Path assigned *PLAUR* to the ME-necrotic region, agreeing to its known roles in extracellular matrix remodeling and known inductions by hypoxia and inflammation (Graham et al, 1998; Smith and Marshall, 2010), Our results suggest that the receptor may involve in repairing the injured regions in or around necrotic regions within tumors, and thus may not be a predictive marker for tumorigenesis. The third example is RNA-binding motif protein 4 (RBM4), which is a post-transcriptional regulator that promotes cell differentiation by modulating alternative splicing and translation of target genes. In some studies, RBM4 was found to be anti-tumor with low expressions indicating more aggressive HCC and poorer prognosis (Chen et al, 2017; Wang et al, 2014); while in other studies, RBM4 was found to be pro-tumor with animal knockdown resulting in reduced liver tumor growth and angiogenesis (Han et al, 2023). COMET’s Path found that *RBM4* is the most correlated with the metabolites from the ME-high-grade region, including PG(18:0/18:2) (Fig. 5B). Thus, the molecule is unlikely to be directly involved in tumorigenesis, but may be involved in immune responses or activations (Gowen et al, 2015). In conclusion, COMET’s Path is a very powerful method to use the discriminative metabolites found by SgME classifiers to help us to interpret and prioritize potential HCC markers for further mechanistic or clinical validations.

### Spatial transcriptomic profiles confirm the tissue localizations of the top correlated RNAs

To further confirm the spatial localization patterns of the significantly correlated gene sets found, we performed spatial transcriptomics profiling based on oligonucleotide-barcoded slides (Ståhl et al, 2016) on tumor sections from two other patients chosen to have low-grade (Grade 1) or high-grade (Grade 4) tumors, respectively (Fig. EV4A). We focused on visualizing the tissue regions around tumor boundaries to capture the normal, stromal, and tumor regions all within the relatively small capture areas on the spatial transcriptomics slides. We found that most of the gene sets were expressed in the expected histological regions. A few interesting trends were observed. First, at the adjacent-normal tissue regions, the ME-normal gene set had higher expression levels than all other gene sets (Fig. EV4B). An example of such a gene is *HSD17B4*, which encodes the D-bifunctional protein (DBP) that breaks down fatty acids in the peroxisomes (Fig. 5G). However, the spatial transcriptomics assays also show that these genes were not spatially restricted to the normal regions only and could also be highly expressed at the tumor regions (Fig. 5G). Thus, many of these ME-normal genes may not be discovered by standard differentially expressed gene analyses that compare tumor versus normal tissues. Second, the ME-low-grade gene set tends to have higher expression levels at the tumor regions, especially in higher-grade tumors (Fig. EV4B). These observations support our hypothesis that the ME-low-grade regions and metabolites are related to tumorigenesis. Third, although the ME-high-grade gene set was also highly expressed at the tumor regions, especially in higher-grade tumors, we found that these genes tend to higher expressions at the normal regions than the ME-low-grade genes (Fig. EV4B). These results agree with the COMET’s Path analysis that adaptive immune processes are enriched at the ME-high-grade regions, and thus these regions or associated metabolites are not restricted to the tumor regions and may also be found at the adjacent-normal regions. Finally, the ME-fibrotic gene set was only expressed in the second tumor, which had a higher fibrosis score assigned by trained pathologists than the first tumor (i.e., Metavir Fibrosis Scores 3 versus 1). The expression of *TERT* clearly exhibited this trend and was almost not expressed in the first tumor (Fig. 5B). Together, our results obtained from COMET’s Path suggest possible biological interpretations of the six MERs and the specific biological processes that the highly abundant putative metabolites localized in these regions may be associated with. Some of these metabolites may be the reactants, products, or regulators of the found biological processes.

### SgME maps reveal the intra-tumor heterogeneity in the MER compositions of HCC tissues

**Figure 6:**
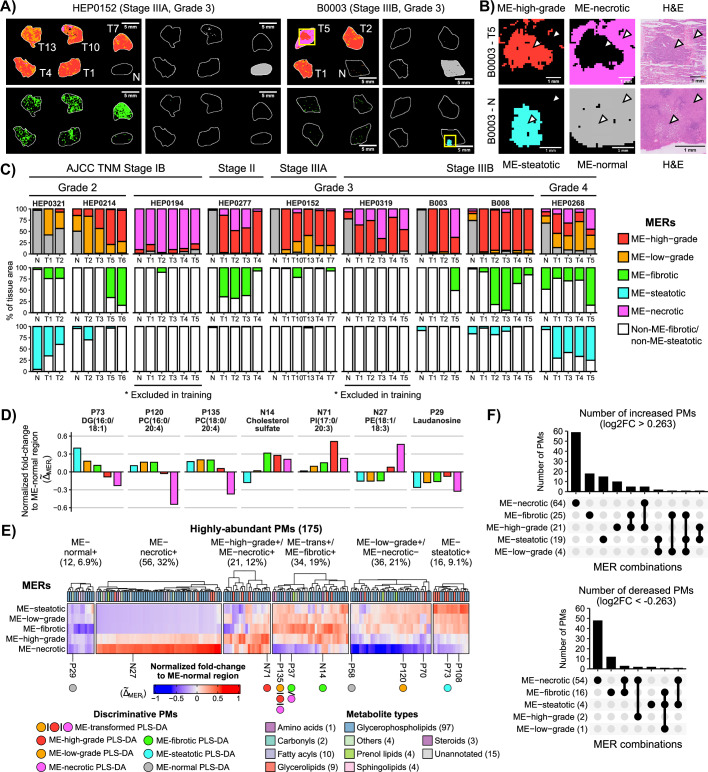
SgME maps reveal intratumoral heterogeneity in the MER compositions of HCC tumor and adjacent-normal tissues. (**A**) Exemplary SgME maps for the liver tumor and adjacent-normal tissue sections collected from four Stage IB to III HCC patients. For visualization only, the predicted ME-normal regions are shown as separate maps (white lines = annotated tissue boundaries). (**B**) Magnified views of selected regions on the SgME maps for patient B0003 (yellow boxes in (**A**)) and their corresponding H&E images (arrows on T5 section = a ME-high-grade region surrounded by a ME-necrotic region; arrows on N section = a ME-steatotic region on top of a ME-normal region). (**C**) Stacked bar charts showing the percentages of tissue areas covered by the predicted MERs. (**D**) Bar charts showing the mean normalized log2 fold change in the abundance levels between each MER to the ME-normal region (${\widetilde{\triangle }}_{{{\mbox{MER}}}_{i}}$) for seven putative metabolites of interest. (**E**) Heatmap showing the ${\widetilde{\triangle }}_{{{\mbox{MER}}}_{i}}$ values for all the highly abundant putative metabolites detected in the whole tissue regions. All the putative metabolites were clustered into six groups using a hierarchical clustering algorithm with Ward’s linkage. Selected discriminative highly abundant putative metabolites for the indicated PLS-DA classifiers were highlighted [P29 = laudanosine, P37 = oleoylcarnitine, P58 = lysoPC(18:1), P70 = DG(16:0/18:3), P73 = DG(16:0/18:1), P108 = PC(15:0/18:0), P120 = PC(16:0/20:4), P135 = PC(18:0/20:4), N14 = cholesterol sulfate, N27 = PE(18:1/18:3), and N71 = PI(17:0/20:3)]. (**F**) MER distributions of highly abundant putative metabolites that were increasing (left) or decreasing (right) compared to the ME-normal regions (numbers after the MER labels = total numbers of putative metabolites detected at the MERs). Source data are available online for this figure.

To survey the spatial metabolomics landscape of HCC, we applied the classifiers to all the DESI-MSI images, including those from patients HEP0194 and HEP0319 (Fig. 3A). The maps revealed the complex and heterogeneous organizations and architectures of TMEs (Fig. 6A; Appendix Fig. S10). We found that some of these spatial structures, especially for ME-high-grade, -necrotic, and -fibrotic regions, are usually spatially associated with clear histopathological features in the H&E images (Fig. 6B, top row). However, other spatial structures, especially for ME-normal, -low-grade, and -steatotic regions, have histological features that are less easily demarcated from the H&E images (Fig. 6B, bottom row).

Furthermore, the full SgME maps confirmed our hypothesis that MERs may overlap on top of each other. For example, we found a ME-steatotic region that overlapped with a ME-normal region in tumor B0003 (lower panels, Fig. 6). Both regions were automatically assigned by our trained PLS-DA classifiers. The DESI images of selected discriminative metabolites, DG(16:0/18:1) and lysoPC(18:1), also show the same trends (tumor B0003, last two rows, Fig. 4G). From the corresponding H&E image, we confirmed that the predicted SgME maps were accurate because lipid droplets could be observed in the predicted ME-steatotic region, while normal hepatocytes could be observed in the predicted (white triangles, lower panels, Fig. 6). Many such instances of overlapping MERs can be found from the full SgME maps for all samples (Appendix Fig. S10). Importantly, ME-steatotic regions do not always overlap with ME-normal regions (for example, sample HEP0152, last two rows, Fig. 4G). If pure spatial clustering of the DESI images were used to identify MERs, many fragmented clusters (e.g., ME-steatotic-only, ME-normal-only, ME-steatotic-plus-normal, and possibly many other combinatorial clusters) would have been found, thereby making downstream consolidations and interpretations of these regions more challenging.

These high-resolution and large spatial metabolomics maps allow us to quantify the MER compositions and their intra-tumor heterogeneity for all the tissue sections (Fig. 6C). The obtained MER compositions generally agree with Edmondson–Steiner’s grades (Edmondson and Steiner, 1954) independently assigned by pathologists. Grade 2 or 3 tumors usually had higher percentages of ME-low-grade or -high-grade regions, respectively (Fig. 6C). However, most of the tumors were composed of heterogeneous MERs. For example, although ME-normal regions covered most of the adjacent normal sections, they could also be found in the tumor sections, such as for patients HEP0321 and HEP0268 (Fig. 6A,C). Most tumor sections had ME-low-grade, -high-grade, and -necrotic regions. Some patients, such as HEP0214, HEP0277, and HEP0268, exhibited high intra-tumor heterogeneity in their ME-transformed-region compositions (Fig. 6C). Interestingly, ME-fibrotic and -steatotic regions could be found in both the tumor and normal-tissue sections. If not resolved, these observed intra-tumor heterogeneities are likely to convolute metabolomics measurements based on bulk samples and lead to false positive or irreproducible markers.

We also found that the SgME maps for the two patients excluded from the classifier training correctly predicted the exclusion reasons of these samples. The SgME map for patient HEP0194 correctly predicted empty ME-normal region in the adjacent—“normal” section from the patient (Fig. 6C). Similarly, the SgME map for patient HEP0319 correctly predicted large ME-necrotic regions (~18.8–65.7%) in all the tumor sections (Fig. 6C). These results show that our classifiers are generalizable and can be applied to new DESI-MSI images unseen by the classifiers before.

### SgME maps reveal the spatial distributions of putative metabolites across different MERs

The SgME maps further allow us to study the spatial distributions of the 230 highly abundant putative metabolites. We quantified their mean abundance levels in every MER and computed their normalized log2 fold changes with respect to the matching ME-normal regions (${\widetilde{\Delta }}_{{{\mbox{MER}}}_{i}}$, “Methods” and Fig. 6D). We found that 175 of these putative metabolites have non-zero mean abundance levels in the ME-normal regions, and their ${\widetilde{\Delta }}_{{{\mbox{MER}}}_{i}}$ values can be divided into six clusters (Fig. 6E). The first cluster, “ME-steototic+ ” (9.1%), was highly abundant in the ME-steatotic regions and mildly abundant in the ME-low-grade and -fibrotic regions. Many of them are glycerolipids. The second cluster, “ME-low-grade+/ME-necrotic-” (21%), was highly increased in the ME-low-grade regions but highly reduced in the ME-necrotic regions, such as PC(16:0/20:4) (P120)—a discriminative putative metabolite for the ME-low-grade classifier. The third cluster, “ME-trans+/ME-fibrotic+ ” (19%), progressively increased from ME-low-grade to -fibrotic regions, such as PC(18:0/20:4) (P135). Some of them were highly abundant in both ME-fibrotic and -high-grade regions, such as cholesterol sulfate (N14) (Fig. 6D). Most of these putative metabolites barely changed in the ME-necrotic regions. The fourth cluster, “ME-high-grade+/ME-necrotic+ ” (12%), had the highest abundance levels in the ME-high-grade and/or -necrotic regions, such as PI(17:0/20:3) (N71) from the ME-high-grade classifier (Fig. 6D). The fifth cluster, “ME-necrotic+ ” (32%), was almost specific to the ME-necrotic regions. Finally, the sixth cluster, “ME-normal+ ” (6.9%), was highly abundant in the ME-normal regions. Among these six metabolite clusters, metabolites from the ME-steatotic+ , ME-low-grade+/ME-necrotic-, and ME-trans+/ME-fibrotic+ clusters are the most important because they may detect early or pre-HCC lesions.

Which MERs generate or deplete the most numbers of metabolites? We found that ME-necrotic region generated the largest number of specific metabolites, followed by ME-fibrotic and then ME-steatotic regions (Fig. 6F, left). The results suggest that necrotic or dying hepatocytes, fibrous septa, and steatotic hepatocytes are likely to release or secrete large numbers of metabolites. Together, the ME-necrotic+ , ME-high-grade+/ME-necrotic+ , and ME-steatotic+ clusters account for ~53.1% of all the detected highly abundant putative metabolites in our study. Surprisingly, we also found that ME-necrotic regions depleted the largest number of specific metabolites (Fig. 6F, right). Most of them are associated with the ME-low-grade+/ME-necrotic- cluster. This is consistent with the metabolism pathways/processes associated with the ME-low-grade regions, likely rapidly dividing hepatocytes (Fig. 5C), being abruptly disrupted when the cells become necrotic.

This systematic clustering of all the putative metabolites also enables us to further analyze the significantly or insignificantly changed putative metabolites found in the earlier tissue-averaged results (Fig. EV2B). As expected, we found that most of the significantly increased putative metabolites came from the ME-steatotic+ or ME-trans+/ME-fibrotic+ clusters, and some of the significantly decreased putative metabolites came from the ME-normal+ cluster (Appendix Fig. S11). Surprisingly, we also found that most of the significantly decreased putative metabolites came from the ME-low-grade+/ME-necrotic- or ME-necrotic+ clusters. Although most putative metabolites from the ME-low-grade+/ME-necrotic- cluster are increased in the ME-low-grade regions, their larger decreases in the ME-necrotic regions reduced their tissue-averaged abundance levels and made them appear to be overall “decreasing”. Similarly, ME-necrotic+ putative metabolites also tend to be significantly decreased and not increased in the tumor sections at the tissue-averaged level (Fig. 6C). Together, our results demonstrate the advantages and potential applications of SgME maps in delineating intra-tumor heterogeneity in the MER compositions of tumor tissues. Most of these results are not obvious under bulk tissue-averaged profiles.

### Regression models trained on SgME maps accurately deconvolve the MER compositions of bulk tumor samples

**Figure 7:**
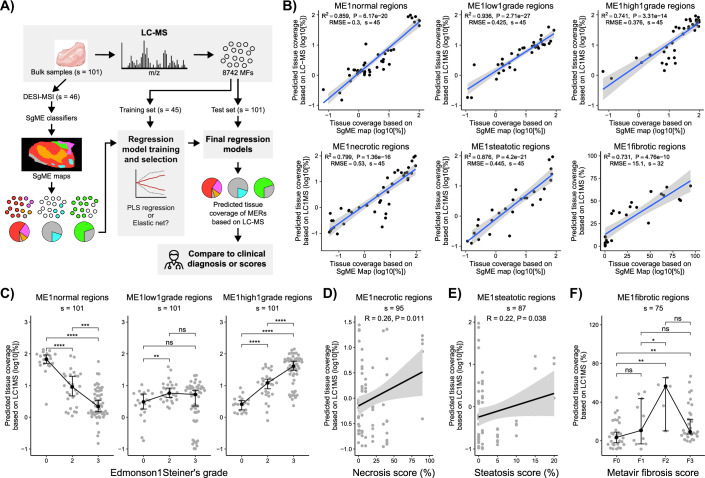
Regression models trained on SgME maps accurately deconvolved the MER compositions of bulk HCC tumor samples. (**A**) Diagram showing the key steps to deconvolve the MER compositions of bulk LC/MS data using regression models trained on SgME maps. (**B**) Scatter plots showing the predicted MER tissue coverages based on LC-MS profiles versus the measured MER tissue coverages based on the SgME maps. Only the optimum regression model for each MER is shown. The performances for all tested regression models are shown in Appendix Fig. S12. (*R*^2^ = coefficients of determination, RMSE =  root mean square errors of the regression models, *P* = *P* values of the t-statistics of the linear regression models.) Comparisons of the predicted MER tissue coverages of all the tissue sections based on LC-MS profiles using the final regression models to the (**C**) Edmonson-Steiner’s Grades, (**D**) necrosis scores, (**E**) steatosis scores, and (**F**) Metavir fibrosis scores assigned by trained pathologists based on independent serial tissue sections collected from the same tumors (for (**C**, **F**), dots =  medians, error bars = 95% confidence intervals of the median values estimated by bootstrapping, n.s. = *P*adj >0.05, *Padj <0.05, **Padj <0.01, ***Padj <0.001, ****Padj <0.0001, one-sided Wilcox rank-sum tests, *P*adj = BH-adjusted *P* values. (**D**, **E**) R = Pearson’s correlation coefficient, gray areas = 95% confidence intervals of the linear regression models. *s* =  number of tissue samples used.). Source data are available online for this figure.

SgME maps can also be used to train regression models to deconvolute heterogeneous tissue samples based on bulk LC-MS profiles (Fig. 7A). To demonstrate this application, we trained either a PLS regression model (Wold et al, 2001) or an elastic net (Zou and Hastie, 2005) (“Methods” and Appendix Fig. S12) to predict the MER compositions quantified from the SgME maps of 46 tissue sections (Fig. 6C). We found that highly accurate regression models can be trained for all six MERs (*R*^2^ ranging from 0.731 to 0.936; 10 × ten-fold cross-validation; Fig. 7B). Although these high R² values were very encouraging, they may reflect some degree of overfitting and should therefore be interpreted with appropriate caution. The true generalizability of the models will require confirmation in independent external cohorts. The final regression models were then applied back to the LC-MS profiles of all patients (*s* = 101), including those without any DESI-MSI data (Fig. 7A).

To understand the relationships between the predicted MER compositions and current clinically-used histopathological scores, we compared the predicted tissue coverages of ME-normal, low-grade, and high-grade regions to Edmondson–Steiner’s grades (Edmondson and Steiner, 1954) independently assigned by pathologists to these cases (Fig. 7C). We found that the predicted % of ME-normal regions significantly decreased from the adjacent normal tissue sections (Grade 0) to Grade 2 and then to Grade 3 sections (*P*_adj_ < 0.0001 and *P*_adj_ < 0.001, respectively; one-sided Wilcoxon rank-sum test). The predicted % of ME-low-grade regions on Grade 2 sections were significantly higher than Grade 0 sections (*P*_adj_ < 0.01; one-sided Wilcoxon rank-sum test), but Grade 3 sections were more heterogeneous and thus its difference to Grade 0 was insignificant. We also found that the predicted % of ME-high-grade regions significantly increased from Grade 0 to Grade 3 sections (*P*_adj_ < 0.0001 and *P*_adj_ < 0.0001, respectively; one-sided Wilcoxon rank-sum test).

We also compared the predicted % of ME-necrotic or -steatotic regions to the necrosis or steatosis scores assigned by pathologists (Fig. 7D,E). We found that the predictions and the scores were mildly positively correlated (*R* = 0.26 and 0.22, *P* < 0.011 and <0.038, respectively). The mild correlations may be due to the high intra-tumor heterogeneity in necrosis or steatosis (Fig. 6C), and are not necessarily indicative of inaccuracy in the predicted values.

Finally, we compared the predicted % of ME-fibrotic regions to the Metavir fibrosis scores (Bedossa and Poynard, 1996) assigned by pathologists (Fig. 7F). F0 is no fibrosis, F1 is portal fibrosis without bands of scar tissue or “septa”, F2 is few septa, F3 is numerous septa without cirrhosis, and F4 is cirrhosis. Since no cirrhotic ROI was annotated for classifier training, we only used tissues from F0 to F3. We found that the predicted % of ME-fibrotic regions of F0 sections were lower than F1 to F3 sections, and the differences between F0 and F2 or F3 sections were significant (both *P* < 0.01, one-sided Wilcoxon rank-sum test). Thus, the predicted ME-fibrotic regions are likely portal-tract fibrosis and/or septa. Together, our results show that the MER compositions predicted by models trained using SgME profiles mostly agree with the histopathological scores.

## Discussion

SgME profiling overcomes the limitation of conventional bulk metabolomics profiling and infers information at a more granular and spatial level. Using this approach, we observed several interesting and reproducible putative metabolites, such as PC(18:0/20:4) and PC(16:0/20:4), which were highly abundant in the ME-low-grade regions (Fig. 4). A previous study of 320 phospholipid species in 32 HCC patients has found that PCs are the most elevated phospholipid class (Liu et al, 2020). However, our results also show that other PCs, such as PC(18:0/20:2) and PC(16:0/18:0), have elevated levels in the ME-steatotic regions that may also localize in the normal tissues too. Another interesting putative metabolite is cholesterol sulfate, which can inhibit gluconeogenesis in hepatocytes by targeting hepatocyte nuclear factor 4α (Shi et al, 2014). However, the cellular origin of the metabolite is unclear from previous studies. Using SgME profiling, we found that it is associated with the ME-fibrotic, -high-grade, and -necrotic regions (Fig. 6D), and thus may be a late-stage HCC marker. Overall, SgME profiling can delineate the spatial localizations of key metabolites in HCC, and provide better understanding of their potential roles in HCC progression.

By using COMET’s Path analysis, we assigned many biological and metabolic processes that appear to be “commonly” changed across all HCC stages at the bulk-tissue level (Fig. EV1) to different MERs in the tumors (Fig. 5C; Appendix Fig. S9). Our results suggest that ME-normal regions can still perform normal liver functions, including fatty acid and lipid metabolisms; ME-low-grade and -steatotic regions may start to actively proliferate and divide; ME-high-grade and ME-necrotic regions may secrete many metabolites and tumor antigens that trigger adaptive immune responses and phagocytosis; and ME-fibrotic and -steatotic regions may activate angiogenesis and/or inflammatory responses. Importantly, we also found that most of the top genes correlated to discriminative metabolites that define the MERs are potential HCC markers known to be overexpressed in HCC or associated with poor prognosis of the disease. Many of these genes were originally discovered via large-scale bioinformatics analyses, and thus have unknown, unclear, or even conflicting previous reports on their exact functions or roles in HCC. COMET’s Path analysis is able to assign most of them to specific MERs or metabolites and hypothesize their potential roles in HCC.

Our study provides many new insights into the metabolic intra-tumor heterogeneity of HCC, which may guide future metabolomics studies. We estimated that >50% of the highly abundant putative metabolites detected from bulk HCC tumors may come from the ME-necrotic, -fibrotic, and/or -steatotic regions. Even though these metabolites may change significantly between tumor and normal tissues, they are unlikely to be predictive and/or reproducible biomarkers of HCC tumorigenesis or development. Furthermore, we discovered a potentially new group of metabolites, namely the ME-low-grade+/ME-necrotic- cluster, which increases at the low-grade tumor regions but dramatically decreases at the necrotic tumor regions. At the tissue-averaged level, these metabolites appear to be “decreasing” in tumors and thus are likely to be missed by bulk metabolomics profiling. Surprisingly, the ME-necrotic regions both generate and deplete the largest numbers of metabolites (Fig. 6F). These abrupt changes in the metabolomics landscapes may indicate metabolic reprogramming via a currently unknown mechanism.

Our approach is general and may be applied to study other cancer types and/or metabolic diseases. Our usage of known histopathological features to identify and label ROIs for classifier training is simple and interpretable, and the identified SgME maps can be easily checked or validated based on molecular pathways known to be associated with these histopathological regions. However, such histopathology-based labeling of the training tissue regions may not fully capture the molecular complexity of MERs and thus may either completely miss a MER type (which may have unknown histology) or partially miss cellular niches with different histological features. To discover novel MERs or fully elucidate MERs, our approach may be further extended by defining ROIs for classifier training based on molecular profiles of key cancer metabolism or tumorigenesis processes (such as RNA or protein expressions or a combination of them). This will require the development of new methodologies to perform and analyze multiplex protein-marker imaging or spatial transcriptomics assays on the same or adjacent freshly frozen tissue sections. Another limitation of our current study is the small number of samples tested using spatial transcriptomics. Follow-up studies based on more tissue samples from different disease stages are needed to confidently map out the spatial localization patterns of the highly correlated genes found using multiplex protein-marker imaging or spatial transcriptomics assays. Also, our metabolite annotations were based on m/z and MS^2^, which is a confidence level II (Sumner et al, 2007). In addition, there could be discrepancies in accurate mass between MSI and LC-MS instruments. Our approach may also be further improved by coupling to ion mobility separation to MSI to enable the separation of isomeric metabolites and the generation of more accurate SgME maps. Our framework could eventually enable systems-level (transcriptomics-proteomics-metabolomics) understandings of the roles of local cellular niches in cancer metabolism and tumorigenesis. This holds the promise of biomarker discovery and subsequent patient stratification for disease treatments and management in HCC and potentially other cancer types. In future studies, we plan to evaluate the predictive performance of this workflow on clinically annotated HCC therapeutic cohorts and assess its potential to unravel predictive biomarkers. Finally, many metabolites are secreted to the circulatory system, including cholesterol sulfate (Strott and Higashi, 2003), and some of the predictive metabolites may also be detected from blood samples. Thus, non-invasive blood-based assays that can infer spatial MER compositions may be developed based on our results and be applied to unresectable tumors as well.

## Methods

**Table:** Reagents and tools table

Reagent/resource	Reference or source	Identifier or catalog number
**Experimental models**		
FF liver tissues (*H. sapiens*)	PLANET study cohort (NCT03267641)	
FFPE liver tissues (*H. sapiens*)	PLANET study cohort (NCT03267641)	
**Recombinant DNA**		
**Antibodies**		
**Oligonucleotides and other sequence-based reagents**		
Dual Index Kit TS Set A	10X Genomics	1000251
**Chemicals, enzymes, and other reagents**		
Acetic acid	Fisher Chemical	A113-50
Acetonitrile (ACN)	Fisher Chemical	047138.K7
Ammonium acetate	Sigma-Aldrich	A2706
Ammonium formate	Sigma-Aldrich	78314
Bluing buffer	Agilent	DKO.CS70230
Dichloromethane (DCM)	Fisher Scientific	AC610050040
Ethanol	Chemtech	-
Ethanol, ≥99.45%	Sigma-Aldrich	E7023
Formic acid	Fisher Chemical	A117-50
Hydrochloric acid	Supelco	1.00317
Isopropanol (IPA)	Fisher Chemical	A4614
Methanol (MeOH)	Fisher Chemical	A4564
NucleoSpin® totalRNA FFPE XS kit	Machery-Nagel	740982.5
Phosphoric acid	Supelco	93752
RNA 6000 Pico kit	Agilent	5067-1513
Visium CytAssist Reagent Accessory Kit	10X Genomics	1000499
Visium CytAssist Spatial Gene Expression for FFPE, Human Transcriptome	10X Genomics	1000520
Visium CytAssist Tissue Slide Cassette, 6.5 mm	10X Genomics	1000471
**Software**		
HistoPath Analytics Platform	https://hpashare.org	
ImageJ/FIJI software v1.54 f	https://imagej.net/software/fiji/	
Loupe Browser v6.2.0	10X Genomics	
Masslynx software v4.2	Waters	
Progenesis QI software v2.0	Nonlinear Dynamics	
QuPath v0.4.3	https://qupath.github.io/	
R statistical software v4.3.1	https://www.r-project.org/	
Space Ranger	10X Genomics	
Waters High-Definition Imaging software v1.4	Waters	
**Other**		
Acquity UPLC with 2777 C auto sampler	Waters	
Agilent Bioanalyzer	Agilent Technologies	
UPLC BEH C8 column	Waters	
Acquity BEH HILIC column	Waters	
High-resolution Waters Synapt G2-Si MS	Waters	
Histocore Spectra ST 839	Leica	
Illumina NovaSeq 6000	Illumina	
Leica microtome RM2265	Leica	
Leica cryostat-microtome CM 1950	Leica	
Philips IntelliSite UltraFast scanner	Philips	
Visium CytAssist	10X Genomics	
Xevo G2-XS QToF mass spectrometry	Waters	
Zeiss Axioscan 7	Carl Zeiss	

### Patient recruitment

Biosamples and clinical phenotypes were obtained prospectively from the PLANet study cohort (NCT03267641), which consisted of patients with primary liver cancers that have undergone surgical resections. PLANet recruitment criteria required the patients to have no extrahepatic metastasis (defined as lymph node <2 cm, lung modules <1 cm, farther lymph nodes <2 cm) with R0 or R1 resection and Child-Pugh ≤7 points without clinical ascites. Patient consents were obtained according to the guidelines of the SingHealth Central Institutional Review Board, which approved the study (CIRB Ref: 2016/2626 and 2018/2112).

### Preparation and processing of the liver tissues

Tumor and adjacent normal liver tissues were collected from surgical resection performed at Singapore General Hospital and National University Hospital Singapore according to a stringent protocol as previously described (Zhai et al, 2022). The resected specimens were transported immediately on ice in a temperature-controlled cooler to a pathologist, where each specimen was subjected to multi-region sampling (Zhai et al, 2022). Non-tumor adjacent normal tissues were defined as segments at least 2 cm away from the tumor. The tumor was then cut through the capsule, photographed, and inspected for fibrosis, necrosis, hemorrhage, and cystic changes. Then, multiple sectors along one axis of a single tumor slice were harvested (Fig. 1A). Depending on the size of the tumor, between 2 and 6 sectors were collected from each patient. Each of these sectors was further divided into three or more parts and subjected to the different omics profiling modalities, including untargeted high-resolution LC-MS profiling (*s* = 109), RNA-seq profiling (*s* = 81), DESI-MSI profiling and H&E imaging (*s* = 52), and spatial transcriptomic profiling (*s* = 2). For each modality, we applied stringent matching criteria and quality-control assessments. Thus, the numbers of matching sections across different omics modalities were different and usually less than the total number of profiled sections (Appendix Table S2). All tissues were snap-frozen immediately and stored at -80 °C, unless otherwise indicated.

### Histopathology diagnosis and evaluation based on clinical diagnosis slides

The tissues were sectioned with a thickness of 4 µm and stained with H&E, examined under a light microscope, and scanned into whole-slide images using a Philips IntelliSite UltraFast scanner (Koninklijke Philips N.V., Amsterdam, Netherlands). The stained tissue sections were evaluated by trained consultant pathologists for histological diagnosis, HCC staging according to the American Joint Committee on Cancer (AJCC) primary tumor, regional lymph nodes, and distant metastasis (TNM) Staging System v8 (Amin et al, 2017), tumor grading according to the Edmondson–Steiner’s Grading (Edmondson and Steiner, 1954), fibrosis scoring according to the Metavir fibrosis scoring system (Bedossa and Poynard, 1996), steatosis scoring according to the percentage of macrovesicular steatosis, and necrosis scoring according to the percentage of tumor necrosis. Both fibrosis and steatosis scores are determined based on the adjacent normal tissues.

### LC-MS data generation

Aqueous and lipid metabolites were extracted using the protocols as previously described (Vorkas et al, 2015). The tissues (50–150 mg) were mixed with pre-chilled methanol/water solution (1:1) and followed by homogenization under liquid nitrogen (Tissue homogenizing CKMix, Bertin Technologies, France). Aliquots of the supernatant were dispensed into Eppendorf tubes and dried under vacuum. The dried supernatant was then re-suspended in 100 μL UPLC-MS grade water/acetonitrile (5:95) before analysis. The lipid metabolites were extracted by adding pre-chilled dichloromethane/methanol (3:1) solution proportionally into the residual pellet and followed by homogenization. A total of 100 μL of the lipid phase supernatant were collected after centrifugation and dried in an extractor hood. The dry material from the lipid extracts of the tissue sample were reconstituted in 200 µL of water/acetonitrile/isopropyl alcohol (1:1:2) before analysis. Quality control samples and dilution series were pooled samples from the extracts. Long-term reference samples were made for batch variation correction for future study, following the same sample preparation procedures.

The samples were analyzed on Xevo G2-XS QToF mass spectrometry (Waters Corporation, Massachusetts, USA) with an electrospray ionization (ESI) source in both positive mode and negative mode. Mass spectrometry instrument parameters were as follows: spray voltage 2.0 kV (positive)/1.5 kV(negative), source temperature 120 °C, cone gas flow 150 L/h, desolvation temperature 60 °C, desolvation gas flow 1000 L/h. The injection volume is 2 µL. Autosampler temperature is 4 °C. Isopropanol was used as a needle wash.

The untargeted metabolic profiles for lipid extracts were separated using UPLC BEH C8 column (1.7 μm, 2.1 × 100 mm) (Waters Corporation, Milford, MA, USA) with both positive and negative ESI modes spray voltage 2.0 kV (positive)/1.5 kV(negative). The columns were maintained at a temperature of 55 °C. Solvent A consisted of 50% water, 25% acetonitrile and 25% isopropanol with 5 mM ammonium acetate, 0.05% acetic acid and 20 µM phosphoric acid. Solvent B consisted of 50% isopropanol and 50% acetonitrile with 5 mM ammonium acetate and 0.05% acetic acid. The total of 13.25 min runtime with an initial flow rate of 0.6 ml/min with 1% solvent B, increasing to 30% solvent B at 2 min, 90% solvent B at 11.5 min, 99.9% solvent B at 12.0 min, held at 99.9% solvent B for 0.5 min before returning to the initial conditions. At the end of the run, 0.75 min of column equilibration with 1% mobile phase B was performed.

The untargeted metabolic profiles of aqueous extracts were separated using an Acquity BEH HILIC column (1.7 μm, 2.1 × 150 mm) (Waters Corporation, Milford, MA, USA) with positive ESI mode, spray voltage 1.5 kV. Solvent A was 20 mM ammonium formate and 0.1% formic acid in water and solvent B was 0.1% formic acid in acetonitrile. The total of 12.65 min runtime with an initial flow rate of 0.6 mL/min with 95% solvent B, decreasing to 80% solvent B at 4.6 min, 50% solvent B at 5.5 min, holding 50% solvent B till 7.0 min, gradually returning to the initial conditions (Zhang et al, 2012; Want et al, 2010). At the end of the run 5.65 min of column equilibration with 95% mobile phase B was performed. The columns were maintained at a temperature of 40 °C.

### LC-MS data processing

The raw LC-MS data were processed using the Progenesis QI software (v2.0; Nonlinear Dynamics, Newcastle, UK), which included automatic alignment using RT, peak picking, and deconvolution. Potential drift in intensity during the analysis was corrected using regression as previously described (Lewis et al, 2016). Missing values were replaced with half minimum intensity values, and all the intensity values were log transformed (Bijlsma et al, 2006). Additional features filtering was performed using the profiles obtained from various dilutions of quality control samples as detailed previously (Want et al, 2010). We extracted 8742 ions from positive aqueous extracts and positive and negative organic extracts of the 109 tissue samples using LC-MS (Appendix Fig. S2). The aqueous ions were mostly small molecules (*m/z* = ~78–811); while the organic ions were usually larger molecules (*m/z* = ~148–1610), such as lipids or fatty acids. Two of the samples had incomplete mass spectra, thus only the remaining 107 samples were further analyzed.

Significantly changed mass features were identified by using two-sided *t* tests between the mean log2 abundance levels in the tumor sections versus the mean log2 abundance levels in the adjacent normal tissue sections. All *P* values were adjusted for multiple testing using the Benjamini–Hochberg (BH) procedure (Benjamini and Hochberg, 1995), and the significant thresholds used were *P*_adj_ < 0.05 and log2-fold change (log2FC) > |0.2630 | , which corresponds to a fold change of 120%.

### RNA-seq data processing

The RNA-seq data were obtained from a previously published study of the PLANEet cohort (Zhai et al, 2022; Transcriptomic 90 patient cohort data, 2022; RNA-seq data for 67 patient cohort, 2022). The RNA-seq data were aligned to GRCh38 reference genome and GENCODE version 33 annotation using the STAR pipeline (Dobin et al, 2013). Raw read counts were quantified using RSEM (Li and Dewey, 2011) with default parameters. All subsequent RNA-seq data analyses were performed under the R statistical software environment (v4.3.1, R Foundation, Austria).

The RSEM raw counts were normalized using DESeq2 package (v1.40.2) (Love et al, 2014) along with log2 transformation for downstream analysis. Low-varying transcripts with IQR less than 10 counts were removed. A total of 16005 transcripts were retained. Differentially expressed genes were identified by using negative binomial generalized linear models (GLM) as implemented in DESeq2 by comparing the mean counts of each gene in the tumor sections to the mean counts of the same gene in adjacent normal tissue sections. All *P* values were adjusted for multiple testing using the BH procedure, and the differentially expressed gene thresholds used were *P*_adj_ < 0.05 and log2-fold change (log2FC) > |0.2630 | .

### Pathway enrichment analysis

Annotated gene sets from the Molecular Signature Database (MSigDB) (Liberzon et al, 2015) package (v7.5.1) were used for RNA expression pathway analysis. A total of 9509 hallmark (H), KEGG (CP:KEGG), Reactome (CP:REACTOME), and GO biological process (GO:BP) gene sets for homo sapiens were used. To filter out potential metabolism-related gene sets, the following keywords were used: “metab”, “lipid”, “catab”, “tty_acid”, “synth”, “egrad”, “TCA”, and “genesis”. The pathway enrichment/de-enrichment analyses and dot plots were performed using the clusterProfiler (v4.8.3) (Wu et al, 2021) and enrichplot (v1.20.3) packages. All *P* values were obtained using two-sided hypergeometric tests and adjusted for multiple testing using the BH procedure. The significant threshold used was *q* value < 0.05 (Storey, 2002).

### Inter- and intratumoral heterogeneity quantification

To quantify the inter-tumoral heterogeneity (InterTH) of a mass feature or gene across normal liver tissue sections (“InterTH-norm” scores), all the pairwise differences between the log2 abundance or expression levels of the mass feature or gene were determined across all the normal tissue sections from different patients. The median value of the InterTH-norm scores indicates the expected biological variation of the mass feature or gene in normal liver tissues.

To quantify the inter- and intratumoral heterogeneities of a mass feature or gene across HCC tumor sections (“InterTH-tumor” and “IntraTH-tumor” scores, respectively), the mean log2 abundance or expression levels for all mass features or genes were first determined for all the tumor sections. Then, the InterTH-tumor scores for the mass feature or gene were the set of all the pairwise differences between the mean log2 abundance or expression levels of the mass feature or gene across all the tumor sections collected from different patients. Similarly, the IntraTH-tumor scores for the mass feature or gene were the set of all the pairwise differences between the log2 abundance or expression levels of the mass feature or gene across all the tumor sections collected from the same patients. The median values of the InterTH-tumor or IntraTH-tumor scores indicate the expected biological variations of the mass feature or gene across tumor sections from different or the same patients, respectively.

Finally, the differences between the median InterTH-tumor or IntraTH-tumor scores to the median of InterTH-norm scores were determined by performing two-sided Wilcoxon rank-sum tests. All *P* values were adjusted for multiple testing using the BH procedure, and the significant threshold used was *P*_adj_ < 0.05.

### MS/MS for putative metabolites identification

Highly abundant mass features were identified based on the 90th-percentile and mean abundance levels of the 8742 mass features across all the tissue samples (Fig. 2B). These mass features were further subjected to data-dependent MS/MS fragmentation experiments using UPLC-ESI-time of flight (QTOF)-MS on Acquity UPLC with 2777 C auto sampler (Waters Corporation, Massachusetts, USA) coupled with Xevo G2-XS QToF mass spectrometry (Waters Corporation, Massachusetts, USA) with the same chromatographical conditions (“LC-MS data generation”) on the pooled quality control samples. The mass features were fragmented using low, medium, and high level of collision energy to obtain the MS/MS fragmentation ion spectra, which were processed and analyzed using the Masslynx v4.2 software (Waters Corporation, Massachusetts, USA). A list of putative metabolites (Appendix Table S3) was subsequently generated based on comparison of the highly abundant mass features and MS/MS fragmentation ions with an in-built database using the Progenesis QI v2.0 software (Nonlinear Dynamics, Newcastle, UK).

### Manual annotations of MS/MS-validated putative metabolites of interest

The retention time and fragmentation patterns obtained from the MS/MS fragmentation experiments were used to identify a set of 11 putative metabolites. Their possible structural assignments were generated out of the multiple results from the screening of mass spectrometry databases, including Human Metabolome Database (Wishart et al, 2022) and Lipid Maps (Conroy et al, 2024). These discriminative putative metabolites were further manually curated by comparing MS/MS spectra to reference spectra available online to provide reasonable annotation and structural attributions (Appendix Table S4). The distinct pattern ions corresponding to these annotated putative metabolites’ structure are shown in Appendix Fig. S8.

### DESI-MSI and H&E imaging

The tissues were embedded in gelatin (Fluka Chemie AG, Massachusetts, USA) before being snap-frozen. They were then sectioned at −15 °C using the CM 1950 Microsystems cryostat-microtome (Leica Biosystems, Wetzlar, Germany) with a thickness of 15 µm, thaw-mounted on glass slides, and stored in a vacuum desiccator at room temperature prior to imaging. Tissue sections were imaged using a high-resolution Waters Synapt G2-Si MS (Waters Corporation, Massachusetts, USA) fitted with a DESI-source (Waters Corporation, Massachusetts, USA). The interface between Synapt and DESI-source was achieved using the Waters High-Definition Imaging software v1.4 (Waters Corporation, Massachusetts, USA). Images acquisitions were performed both in positive and negative ion mode over the mass range of 50–1200 *m/z* using ionization voltage: 3 kV; cone voltage: 50 V; source temperature: 150 °C; scan time: 1 sec; solvent: methanol:water (98:2) containing 0.1% formic acid; solvent flow rate: 2 μL/min; leucine enkephalin lockmass: 100 pg/μL; pixel size: 100 μm. The captured DESI-MSI raw data were converted and saved as imzML files. The DESI-MSI images for patient HEP0276 were found to have much lower abundance levels than all other images, and it was suspected that the tissue sample may be degraded. Thus, the patient was removed from our study.

After performing DESI-MSI, the tissue sections were stained with H&E and scanned into whole-slide images using a Philips IntelliSite UltraFast scanner (Koninklijke Philips N.V., Amsterdam, Netherlands) at ×20 magnification. The H&E images were processed and visualized using the HistoPath Analytics (HPA) Platform (Bioinformatics Institute, Singapore).

### DESI-MSI data processing

The DESI-MSI data were loaded and analyzed using the Cardinal MSI package (v3.2.1) (Bemis et al, 2015) under the R statistical software environment (v4.3.0, R Foundation). The spatial abundance levels of highly abundant putative metabolites were determined by matching the m/z values of their LC-MS ions to the loaded DESI-MSI spectra using the MsCoreUtil package (v1.12.0) (Rainer et al, 2022). The abundance levels were further normalized by computing their log2 fold changes with respect to the mean abundance levels of a set of highly abundant peaks that were commonly and uniformly found across all the tissue sections.

### Tissue boundary annotations

To visualize tissue sections on DESI-MSI images, the sum of the abundances of all ions detected at a spot (total ion current or “TIC”) was computed and exported as PNG files for all samples using the png (v0.1-8) package. Then, the boundaries of all the tissue sections were manually traced on the TIC images using the ImageJ/FIJI software (v1.54 f, National Institutes of Health, USA) (Schindelin et al, 2012) and saved as ImageJ ROIs. The coordinates of these tissue boundaries were loaded back into R using the RImageJROI (v0.1.2) package for future analysis.

### Histological ROI annotations

The H&E images of the tissue sections were annotated by a trained consultant pathologist. Random sections were cross-checked by another trained consultant pathologist, and discordance was resolved by consensus. Image annotation and visualization were performed using the QuPath software (v0.4.3, University of Edinburgh, UK) (Bankhead et al, 2017) and the HistoPath Analytics (HPA) Platform (Bioinformatics Institute, Singapore). For each normal or tumor section, up to three different histological region types were first marked on the H&E image of the section. Regions with hepatocytes displaying ample cytoplasm and minimal nuclear irregularities were marked as the “well-differentiated” regions. Regions with hepatocytes displaying greater nuclear irregularities and angulations were marked as the “moderately differentiated” regions. Regions with hepatocytes displaying increased nuclear pleomorphisms and angulations were marked as the “poorly differentiated” regions. These three region types did not necessarily cover the whole tissue sections, because some of the tissue regions might have hepatocytes with unclear or different phenotypes than these three defined histological features. After that, up to three additional histological region types were further marked on the same H&E image. Regions with the presence of macro- and/or micro-vesicular hepatocytes were marked as the “steatotic” regions. Regions with fibrous septa, where cells had less uniformly rounded morphologies and exhibited more pinkish eosin stains, are marked as the “fibrotic” regions. Regions with mesh-like patterns characterized by the absence of hepatocytes and/or more purplish eosin stains are marked as the “necrotic” regions. These three additional region types may overlap with the first three region types or the unannotated tissue regions.

Within each of these marked regions, one or more representative square ROIs for these histological phenotypes were further highlighted. The locations of these ROIs on the corresponding DESI-MSI TIC images were manually matched using the ImageJ/FIJI software (v1.54 f, National Institutes of Health, USA) (Schindelin et al, 2012). The sizes of these square ROIs on the DESI-MSI images were 8 × 8 pixels for well, moderately, and poorly differentiated regions; and 4 × 4 pixels for necrotic, fibrotic and steatotic regions. A total of 116 no-overlapping ROIs were annotated and matched.

### MER definitions and PLS-DA classifiers

The MERs were fully defined by PLS-DA classifiers that were trained based on the metabolomic profiles of the initial local cellular niches (i.e., small square ROIs) annotated as described under “Histological ROI annotations”. Thus, both the mass features and histological labels of these initial local cell niches influenced the MERs identified by the classifiers. To ensure reproducibility, only 117 highly abundant putative metabolites that could be detected in >50% of all the 116 ROIs were used for training. To reduce the contributions of noise or spurious peaks, the mean abundance levels of these 117 peaks were computed for each ROIs and used for the classifier training (Fig. EV3). The PLS-DA classifiers were trained using the ropls package (v1.3.2) (Thévenot et al, 2015). For each classifier, the optimum number of predictive components (predI) was tuned to be the largest number of components before the predictive performance (Q2Y) (Wold et al, 2001) stopped increasing or started to decrease. The Q2Y values were estimated using a fivefold cross-validation procedure (Kohavi, 1995). The statistical significance levels of the Q2Y value were estimated by randomly permuting the labels. All the PLS-DA classifiers reported in this study have *P* values < 0.05. The final trained PLS-DA classifiers were applied to the whole MSI images of all samples, including those not used for training, to obtain the full-tissue maps for all the MERs.

### SVM classifiers

The SVM classifiers were trained using the Caret package (v6.0-94). The optimum parameters for SVMs were estimated using grid searches of possible parameter values. For SVMs based on radial-basis-function kernels, the searched values were sigma ∈ {10^−4^, 10^−3^, …, 10^1^, 10^2^} and cost = ∈ {2^−2^, 2^−1^, …, 2^6^, 2^7^}. For SVMs based on linear kernels, the searched values were cost = ∈ {2^−2^, 2^−1^, …, 2^6^, 2^7^}. The training and test performances of the classifiers were estimated using a 10 × 10 cross-validation procedure (Kohavi, 1995).

### COMET’s path analysis

For each final trained PLS-DA classifier, putative metabolites with Variable Influence on Projection (VIP) (Wold et al, 2001) > 0.95 and regression coefficients (*β*) >0.03 were selected to be the “discriminative” putative metabolites. The numbers of discriminative putative metabolites selected were 16, 15, 12, 13, 3, and 7 for ME-normal, -low-grade, -high-grade, -necrotic, -fibrotic, and -steatotic region classifiers, respectively. Then, all the low-varying transcripts with IQR < 2 from the RNA-seq dataset were removed. The Spearman’s rank correlation coefficients between the expression levels of all the remaining 6679 transcripts and the abundance levels of all the discriminative putative metabolites were computed across all the matching bulk tissue samples with both RNA-seq and LC-MS measurements. All the changed transcripts from the bulk RNA-seq experiments were ranked according to their mean absolute rank correlation coefficients to the discriminative putative metabolites from each of the six MERs across all the tissue sections. Then, Gene Set Enrichment Analysis (GSEA) (Subramanian et al, 2005) was used to rank gene lists and identify significantly enriched pathways from MSigDB (“Pathway enrichment analysis”) at an adjusted *p*-value threshold of 0.05. To identify the top correlated genes, all the genes were filtered according to their rank correlation coefficients to all the discriminative metabolites. Only those genes with significantly positive correlations with at least 50% of the discriminative metabolites were retained (Spearman’s test; *P*adj < 0.05; all *P* values were adjusted using the BH procedure) (Benjamini and Hochberg, 1995). The ME-normal, -low-grade, -high-grade, -necrotic, -fibrotic, and -steatotic correlated gene sets had 14, 67, 18, 231, 342, and 65 genes, respectively. The survival analysis was performed using cBioPortal (Cerami et al, 2012) on a liver hepatocellular carcinoma dataset of 372 patients from the TCGA PanCancer Atlas (Liu et al, 2018). The high- and low-expression samples were divided based on the mRNA expression z-scores relative to normal samples at a threshold of 1.5.

### Spatial transcriptomics data generation

The tissues were fixed with formalin and embedded in paraffin to produce formalin-fixed paraffin-embedded (FFPE) blocks. Then, the FFPE tissues were processed following the protocols in “Visium CytAssist Spatial Gene Expression for FFPE – Tissue Preparation Guide” (CG000518 Rev C, 10X Genomics, Pleasanton, USA). The FFPE blocks were sectioned using a Leica RM2265 microtome (Leica Biosystems, Wetzlar, Germany) with a thickness of 10 µm and stored in a desiccator box at room temperature. Genomic RNA was isolated using a NucleoSpin totalRNA FFPE XS kit (MACHEREY-NAGEL GmbH & Co, Dueren, Germany) according to the manufacturer’s instructions. Total RNA was eluted with 20 mL of RNase-free water. The DV200 values were estimated using the RNA 6000 Nano Kit and Agilent Bioanalyzer (Agilent Technologies Inc., California, USA) and samples with DV200 > 50% were used for subsequent steps.

FFPE tissue sections were de-paraffinized, stained for H&E, and subsequently imaged at ×20 resolution using a slide scanner (Zeiss Axioscan 7, Carl Zeiss AG, Baden-Württemberg, Germany). After decoverslipping and de-crosslinking, tissue sections were hybridized to probe pairs with human-specific probes targeting 18,536 genes (Visium Human Transcriptome Probe Set v2.0, 10X Genomics, Pleasanton, USA). The transcriptomic probes from the standard glass slides were transferred to the capture areas (6.5 × 6.5 mm) on oligonucleotide-barcoded slides (10X Genomics, Pleasanton, USA) using a slide alignment and sample transfer tool (Visium CytAssist, 10X Genomics, Pleasanton, USA). The probe pairs are extended to incorporate complements of the spatial barcodes, and sequencing libraries are prepared following the protocols in “Visium CytAssist Spatial Gene Expression Reagent Kits User Guide” (CG000495 Rev E, 10X Genomics, Pleasanton, USA). Lastly, the libraries are sequenced by a service provider (Novogene-AIT Inc., Singapore) using an Illumina NovaSeq 6000 with 50,000 reads per spot targeted sequencing depth. The two tissue sections had 4658 and 4992 spots, respectively. The median numbers of detected genes per spot were 3621 and 1812, respectively.

### Spatial transcriptomics data analysis

The FASTQ files and H&E images of the FFPE tissue sections (“Spatial transcriptomics data generation”) were processed using the Space Ranger software (v.2.0.0, 10X Genomics, Pleasanton, USA). The gene expression matrix associated with each spot was loaded into the R statistical software environment (v4.3.1, R Foundation, Austria) using the Seurat package (v5.0.0) (Hao et al, 2024), and normalized and variance-stabilized using the sctransform package (v0.4.1) (Hafemeister and Satija, 2019). Transcripts with <100 total counts across all spots were removed from further analysis. For each spot, the normalized count for a transcript was further normalized by dividing it by the mean normalized count for all transcripts at the same spot.

After that, the spatial distribution of each transcript on each tissue section was quantified as a discrete probability distribution function by dividing its normalized count on each spot to its total normalized count across all spots from the tissue section. In general, we do not expect all the positively correlated genes to be highly co-expressed together at the same time. Thus, for each gene set, the 75th percentile (Q3) expression probability of all the detected genes in the gene set at each spot was determined and visualized over the whole captured region. The values for all spots were visualized using the SpatialFeaturePlot() function in the Seurat package and saved as PNG files.

### SgME map constructions

For every tissue section, three PLS-DA classifiers were trained based on the annotated square ROIs to construct three SgME maps. The normalized abundance levels of the highly abundant putative metabolites were determined and averaged across all the pixels within each of the ROIs on the DESI-MSI images. If a ROI had any undetected putative metabolite, a K^th^ nearest neighbor (KNN) algorithm from the impute package (v1.74.1) was used to impute the missing value. The first classifier (“ME-transformed”) was a multi-class classifier trained to classify ME-normal, -low-grade, -high-grade, and -necrotic regions based on the DESI-MSI profiles of the well, moderately, poorly differentiated, and necrotic ROIs. The second classifier (“ME-fibrotic”) was a binary classifier trained to classify ME-fibrotic and -non-fibrotic regions based on the DESI-MSI profiles of the fibrotic and well-/moderately-/poorly differentiated/steatotic ROIs. The third classifier (“ME-steatotic”) was a binary classifier trained to classify ME-steatotic and -non-steatotic regions based on the DESI-MSI profiles of the steatotic and well-/moderately-/poorly differentiated/fibrotic/necrotic ROIs. Once trained, these three final classifiers were applied to all the pixels within the tissue regions (“Tissue boundary annotations”) to yield three SgME maps for the tissue section. Only for classifier applications, if any pixel had an undetected putative metabolite, the minimum value of the putative metabolite across all other pixels was used to replace the missing value. For visualization only, the ME-normal regions were plotted on separate maps from other ME regions predicted by the ME-transformed classifiers.

### Metabolite clustering based on SgME maps

After the SgME maps were constructed, the abundance levels for all the highly abundant putative metabolites at every single spot/pixel within the annotated tissue boundaries (“Tissue boundary annotations”) on the DESI-MSI images were loaded and normalized (“DESI-MSI data preprocessing”). A total of 175 out of 230 putative metabolites were detected in ≥1% of the total loaded tissue area, and the rest of the putative metabolites were rare or mostly zeros and thus not used for clustering. For each retained putative metabolite, its mean abundance levels in every predicted MER across all the SgME maps were computed, where ${I}_{{{\rm{pixel}}}_{j}}$ is the abundance level on the *j*th pixel of the *i*th MER on the maps.

$${\mu }_{{{{\rm{MER}}}}_{i}}=\left(\frac{1}{|{{{\rm{MER}}}}_{i}|}\right)\cdot \mathop{\sum }\limits_{{{{\rm{pixel}}}}_{j}\in {{{\rm{MER}}}}_{i}}{I}_{{{{\rm{pixel}}}}_{j}}$$

Then, the log2 fold changes between the mean abundance levels in all the MERs and the ME-normal region were determined,

$${\Delta }_{{{{\rm{MER}}}}_{i}}={\log }_{2}\left({\mu }_{{{{\rm{MER}}}}_{i}}/{\mu }_{{{\rm{ME}}}-{{\rm{normal}}}}\right)$$

and normalized by dividing the obtained values by the sum of absolute magnitude of the mean abundance levels across all MERs,

$${\widetilde{\Delta }}_{{{{\rm{MER}}}}_{i}}={\Delta }_{{{{\rm{MER}}}}_{i}}/\left(\mathop{\sum }\limits_{{{{\rm{MER}}}}_{i}}|{\Delta }_{{{{\rm{MER}}}}_{i}}|\right).$$

The normalized abundance log2 fold change (${\widetilde{\Delta }}_{{{\rm{MER}}}_{i}}$) allows us to identify the MER with the lowest or highest fold changes (increasing or decreasing). The normalized abundance fold changes for all the putative metabolites and MERs were rearranged into a matrix, clustered, and visualized using the ComplexHeatmap package (v2.16). The clustering of all the putative metabolites was performed using a standard hierarchical clustering algorithm with Euclidean distance and Ward’s linkage.

### Computational deconvolution of bulk LC/MS data

The tissue coverages of six MERs (ME-normal, -low-grade, -high-grade, -necrotic, -fibrotic, and -steatotic) were quantified by using the SgME maps for all 34 tissue sections (“SgME map constructions”). For each MER, the tissue coverage on a section was the number of pixels for the MER on the SgME map divided by the total number of pixels in the map. Among the quantified sections, 33 of them also had matching LC-MS measurements and were used as training data to train regression models to predict the tissue coverages of these six MERS. For each MER, both elastic nets and PLS regression models were trained based on either all the mass features or the highly abundant mass features. The glmnet (v4.1.8) (Zou and Hastie, 2005) and ropls (v1.3.2) (Thévenot et al, 2015) packages were used to train these models. The built-in cross-validation procedures in these two packages were used to determine the optimum model parameters. The best regression model and mass feature sets with the highest training coefficient of determination (*R*^2^) values were chosen and applied to all the samples with LC-MS measurements.

## Supplementary information

Peer Review File

Source data Fig. 2

Source data Fig. 3

Source data Fig. 4

Source data Fig. 5

Source data Fig. 6

Source data Fig. 7

Appendix

Expanded View Figures

## Data Availability

All raw LC-MS and DESI-MSI datasets are publicly available through EMBL’s MetaboLights database (accession number MTBLS13432). All raw spatial transcriptomics datasets are publicly available through NCBI’s Gene Expression Omnibus (accession number GSE312327). All raw H&E images are publicly available through A*STAR’s ImmunoAtlas (Lee et al, 2021) (https://immunoatlas.org/CCPA/251202-1 and https://immunoatlas.org/CCPA/251202-2). All raw RNA-seq datasets are available through the European Genome-phenome Archive (accession numbers EGAD00001008648; and EGAD00001009042). The source code and processed data for generating all the results and graphs can be downloaded from https://github.com/ccpagroup/sgme-hcc. The source data of this paper are collected in the following database record: biostudies:S-SCDT-10_1038-S44320-026-00205-w.
