# Supplementary figures and images for: Spatially-guided metabolomics profiling of metabolic regions in human tumor tissues
Source: Mol Syst Biol. 2026 Apr 1;22(7):1132–60. doi: 10.1038/s44320-026-00205-w (PMC13328631; doi:10.1038/s44320-026-00205-w)

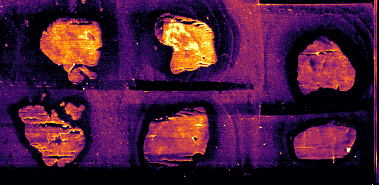

Supplement: Supplementary file 2 — Source data Fig. 2 [file 44320_2026_205_MOESM2_ESM.zip › Figure 2/2E/tic_A-01-01_1_neg.tif]

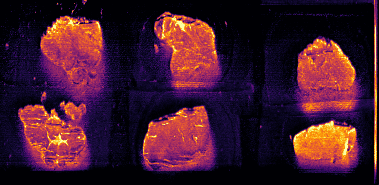

Supplement: Supplementary file 2 — Source data Fig. 2 [file 44320_2026_205_MOESM2_ESM.zip › Figure 2/2E/tic_A-01-01_1_pos.tif]

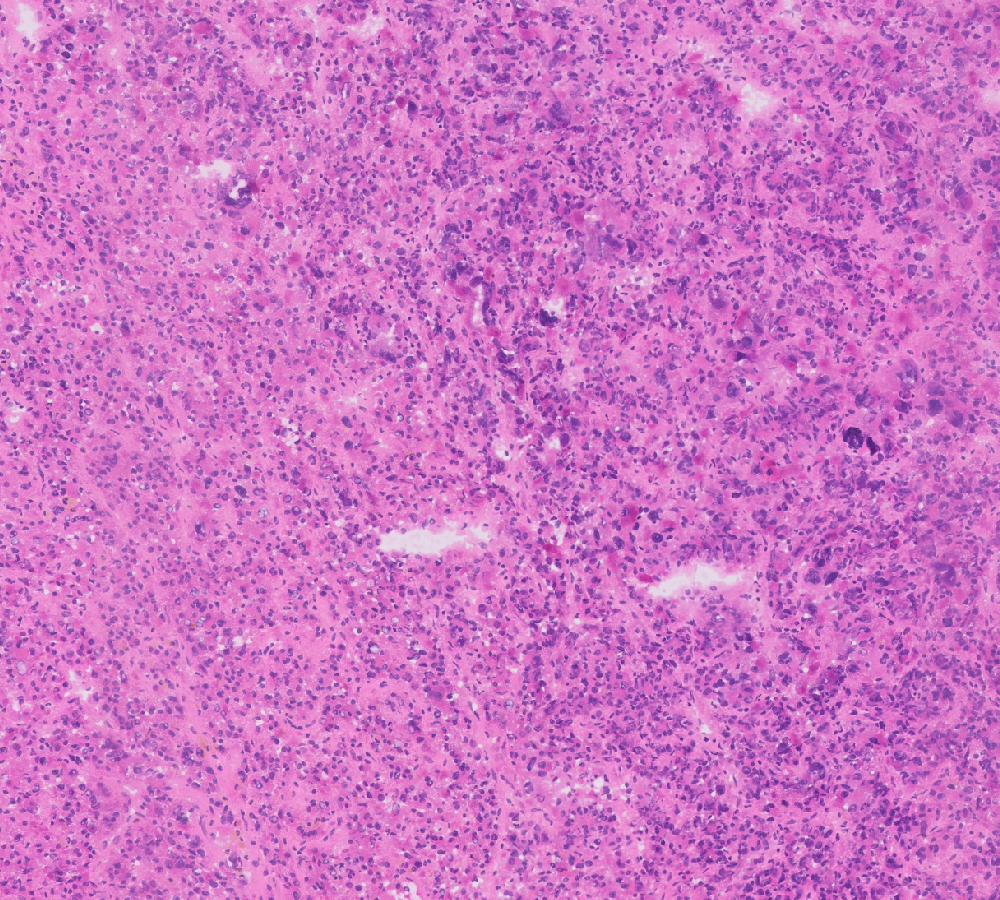

Supplement: Supplementary file 3 — Source data Fig. 3 [file 44320_2026_205_MOESM3_ESM.zip › Figure 3/3B/HEP0152.png]

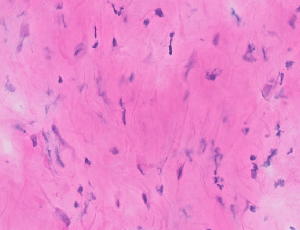

Supplement: Supplementary file 3 — Source data Fig. 3 [file 44320_2026_205_MOESM3_ESM.zip › Figure 3/3C/Fibrosis.png]

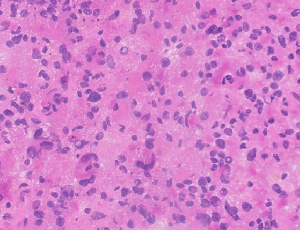

Supplement: Supplementary file 3 — Source data Fig. 3 [file 44320_2026_205_MOESM3_ESM.zip › Figure 3/3C/Moderately_differentiated.png]

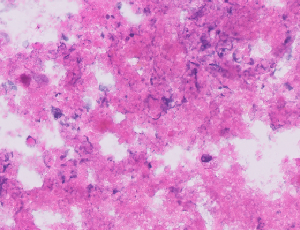

Supplement: Supplementary file 3 — Source data Fig. 3 [file 44320_2026_205_MOESM3_ESM.zip › Figure 3/3C/Necrosis.png]

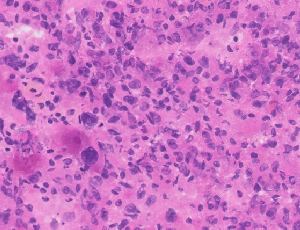

Supplement: Supplementary file 3 — Source data Fig. 3 [file 44320_2026_205_MOESM3_ESM.zip › Figure 3/3C/Poorly_differentiated.png]

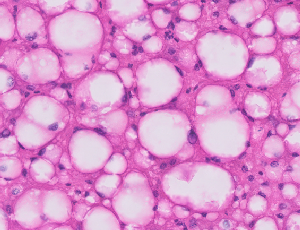

Supplement: Supplementary file 3 — Source data Fig. 3 [file 44320_2026_205_MOESM3_ESM.zip › Figure 3/3C/Steatosis.png]

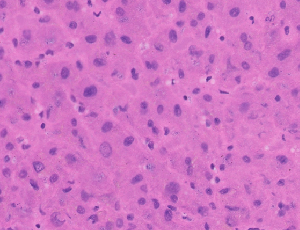

Supplement: Supplementary file 3 — Source data Fig. 3 [file 44320_2026_205_MOESM3_ESM.zip › Figure 3/3C/Well_differentiated.png]

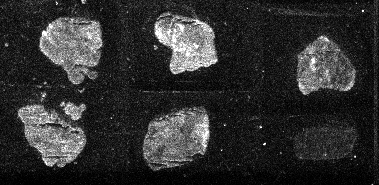

Supplement: Supplementary file 4 — Source data Fig. 4 [file 44320_2026_205_MOESM4_ESM.zip › Figure 4/4G/A-01-01_1_neg_N14.tif]

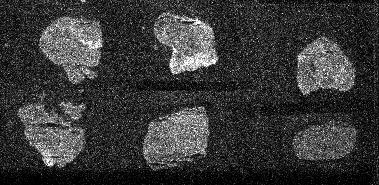

Supplement: Supplementary file 4 — Source data Fig. 4 [file 44320_2026_205_MOESM4_ESM.zip › Figure 4/4G/A-01-01_1_neg_N71.tif]

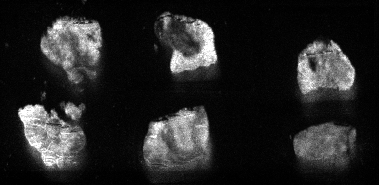

Supplement: Supplementary file 4 — Source data Fig. 4 [file 44320_2026_205_MOESM4_ESM.zip › Figure 4/4G/A-01-01_1_pos_P120.tif]

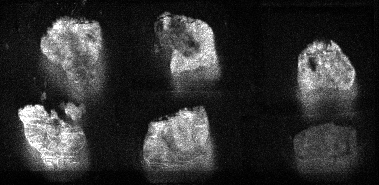

Supplement: Supplementary file 4 — Source data Fig. 4 [file 44320_2026_205_MOESM4_ESM.zip › Figure 4/4G/A-01-01_1_pos_P135.tif]

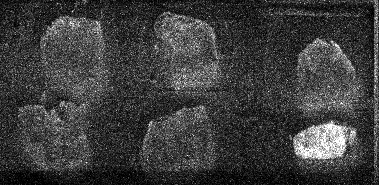

Supplement: Supplementary file 4 — Source data Fig. 4 [file 44320_2026_205_MOESM4_ESM.zip › Figure 4/4G/A-01-01_1_pos_P58.tif]

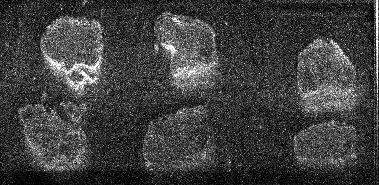

Supplement: Supplementary file 4 — Source data Fig. 4 [file 44320_2026_205_MOESM4_ESM.zip › Figure 4/4G/A-01-01_1_pos_P73.tif]

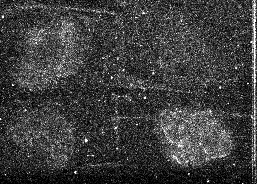

Supplement: Supplementary file 4 — Source data Fig. 4 [file 44320_2026_205_MOESM4_ESM.zip › Figure 4/4G/A-10-01_1_neg_N14.tif]

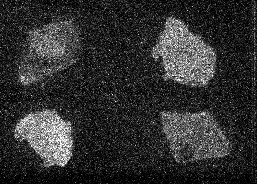

Supplement: Supplementary file 4 — Source data Fig. 4 [file 44320_2026_205_MOESM4_ESM.zip › Figure 4/4G/A-10-01_1_neg_N71.tif]

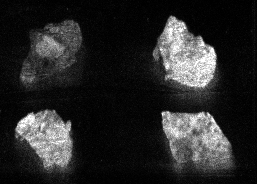

Supplement: Supplementary file 4 — Source data Fig. 4 [file 44320_2026_205_MOESM4_ESM.zip › Figure 4/4G/A-10-01_1_pos_P120.tif]

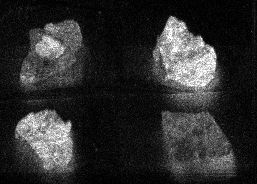

Supplement: Supplementary file 4 — Source data Fig. 4 [file 44320_2026_205_MOESM4_ESM.zip › Figure 4/4G/A-10-01_1_pos_P135.tif]

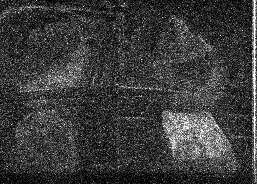

Supplement: Supplementary file 4 — Source data Fig. 4 [file 44320_2026_205_MOESM4_ESM.zip › Figure 4/4G/A-10-01_1_pos_P58.tif]

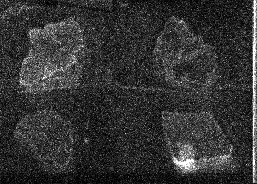

Supplement: Supplementary file 4 — Source data Fig. 4 [file 44320_2026_205_MOESM4_ESM.zip › Figure 4/4G/A-10-01_1_pos_P73.tif]

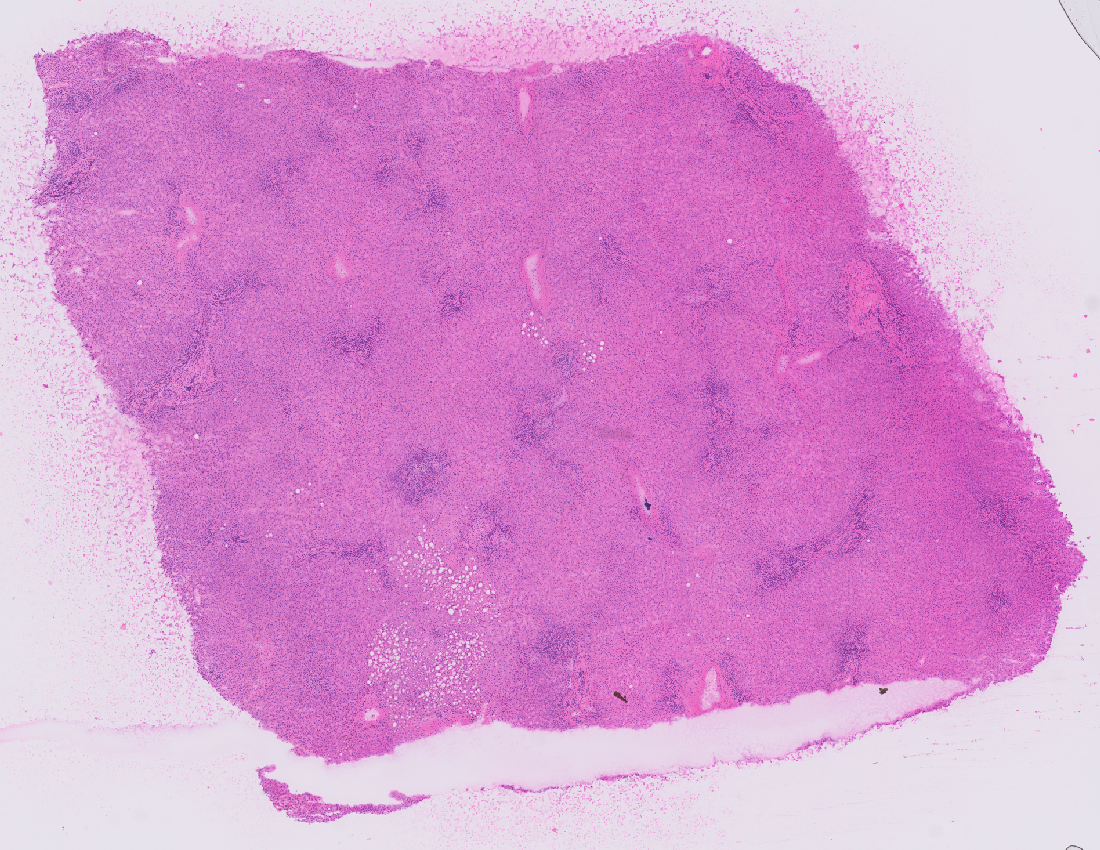

Supplement: Supplementary file 6 — Source data Fig. 6 [file 44320_2026_205_MOESM6_ESM.zip › Figure 6/6B/B0003_N.png]

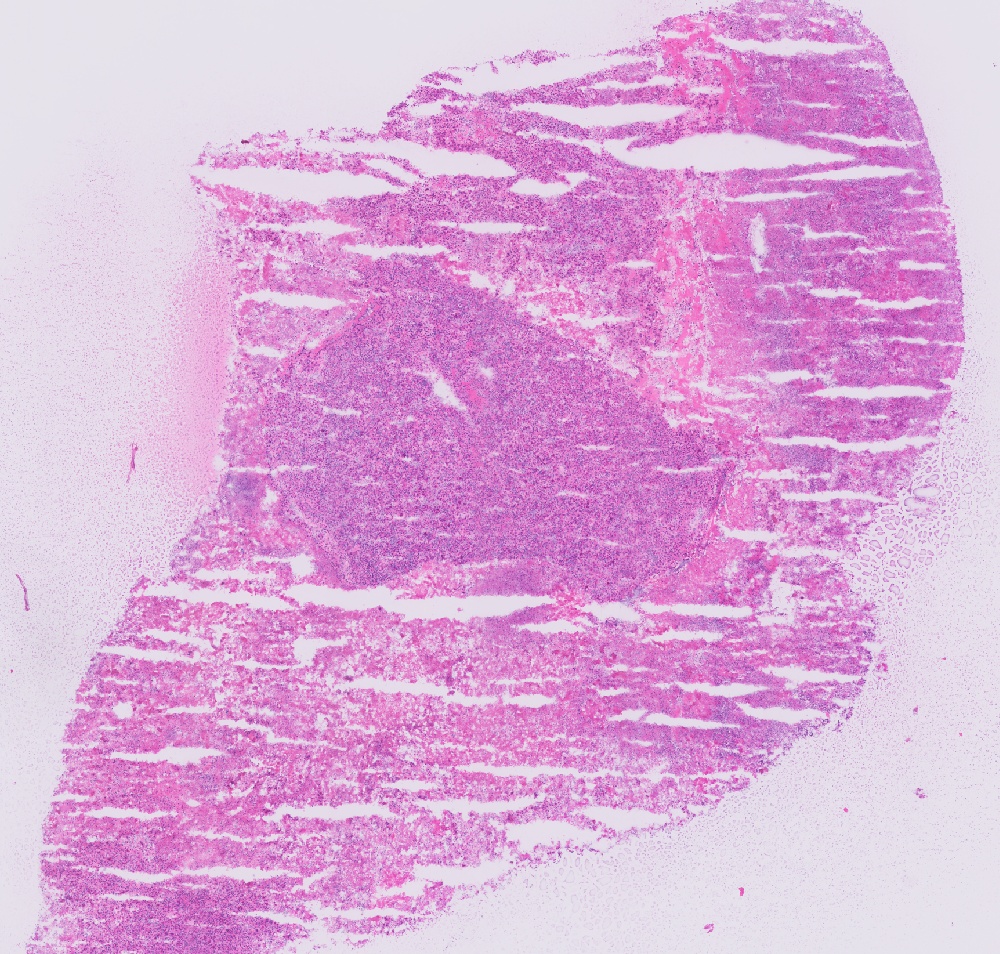

Supplement: Supplementary file 6 — Source data Fig. 6 [file 44320_2026_205_MOESM6_ESM.zip › Figure 6/6B/B0003_T5.png]
