# Supplementary material for: Spatially-guided metabolomics profiling of metabolic regions in human tumor tissues
Source: Mol Syst Biol. 2026 Apr 1;22(7):1132–60. doi: 10.1038/s44320-026-00205-w (PMC13328631; doi:10.1038/s44320-026-00205-w)
Supplement: Supplementary file 8 — Appendix [file 44320_2026_205_MOESM8_ESM.pdf]

Appendix to Spatially-guided metabolomics profiling of metabolic regions in human tumor tissues

Contents

1	Appendix Figure S1. Liver samples used in our study	2
2	Appendix Figure S2. LC-MS mass features detected on HCC tissue samples	3
3	Appendix Figure S3. Global metabolomics profiles of adjacent normal, HCC, and iCCA tumors	3
4	Appendix Figure S4. Distributions of changed transcriptomic or metabolomic profiles across the three HCC stages	4
5	Appendix Figure S5. Biological pathways and processes enriched in genes that are differentially expressed at different HCC stages	5
6	Appendix Figure S6. Additional exemplary H&E image showing heterogeneous histological features within the same HCC tumor	6
7	Appendix Figure S7. MER classification performances of the support vector machines	6
8	Appendix Figure S8. MS/MS spectra and fragmentation patterns of the 11 manually annotated PMs	7
9	Appendix Figure S9. Biological pathways and processes enriched in RNAs correlated to the discriminative PMs for different MERs	10
10	Appendix Figure S10. H&E images and SgME maps for the tissue sections collected from nine HCC patients	11
11	Appendix Figure S11. Significantly changed PMs from the six PM clusters	12
12	Appendix Figure S12. Performances of all the tested regression models for SgMERdeconv	13
13	Appendix Table S1: Clinical information for all the 26 patients studied	14
14	Appendix Table S2: Number of tissue sections analyzed using different spatial and/or non-spatial omics technologies in our study	16
15	Appendix Table S3: List of highly-abundant putative metabolites found and their information	18
16	Appendix Table S4: List of manually-annotated putative metabolites and their information	24

Appendix Figures S1 to 12

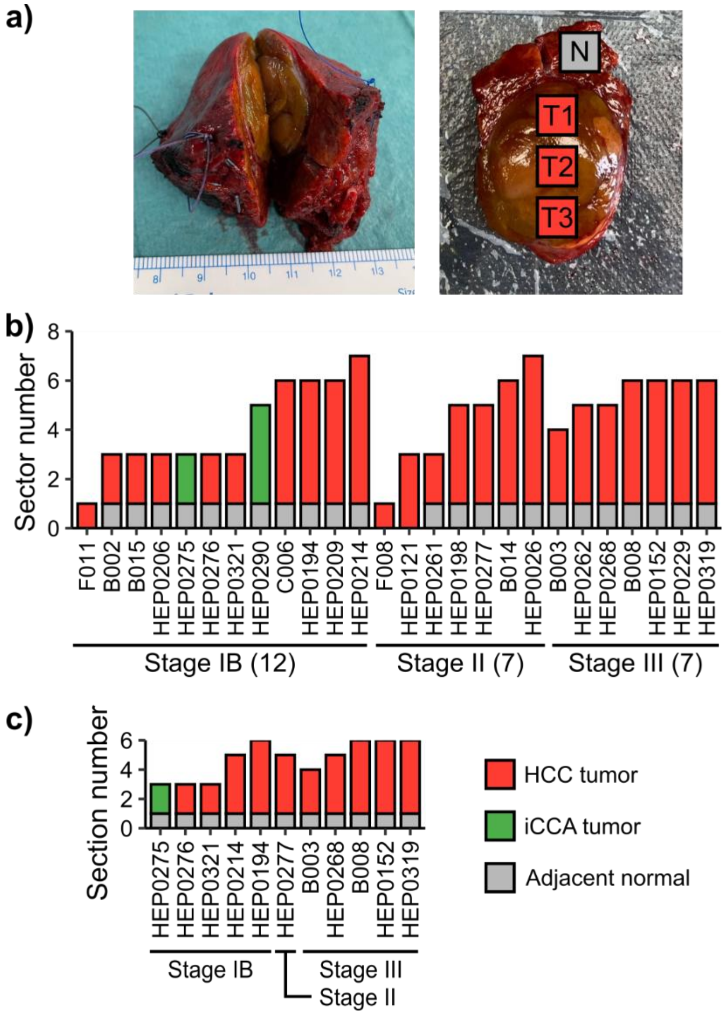

Appendix Fig. S1. Liver samples used in our study. a) Photos showing an exemplary primary HCC tumor (left) and the locations of the tissue sectors, N, T1, T2, and T3, collected from the tumor (right). Bar charts showing the numbers of liver tissue sectors **b)** collected per patient and **c)** subjected to DESI-MSI analysis (Stages IB, II, and III = AJCC TNM Stages IB, II, and III HCC tumors).

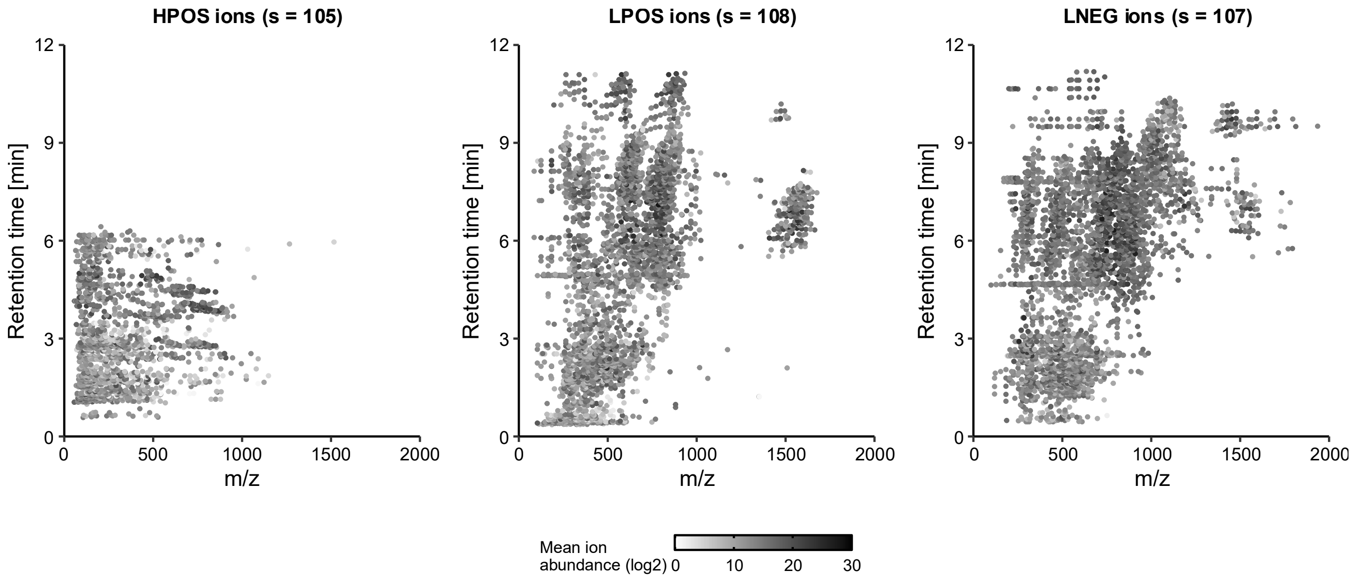

Appendix Fig. S2. LC-MS mass features detected on HCC tissue samples. Scatter plots showing the mean abundance levels of all the 8742 MFs detected using LC-MS from the positive aqueous (HPOS), positive organic (LPOS) and negative organic (LNEG) extracts of all the HCC tissue samples. Each MF is characterized by retention time (min) and mass-to-charge ratio (m/z).

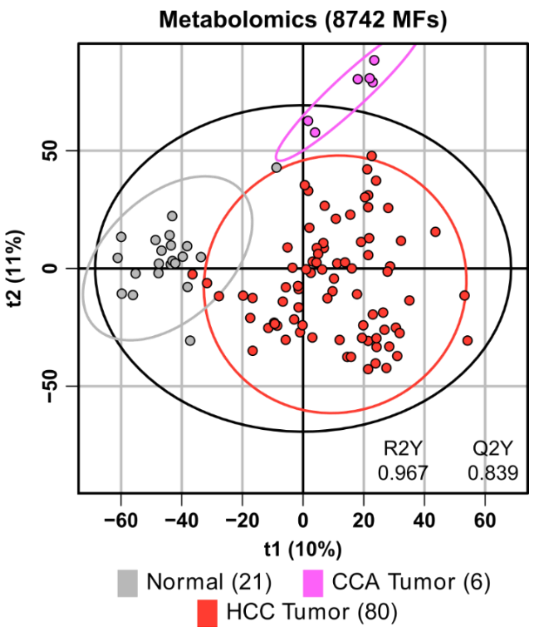

Appendix Fig. S3. Global metabolomics profiles of adjacent normal, HCC, and iCCA tumors. PLS-DA score plot showing the metabolomics profiles of all the tissue sections based on 8742 mass features mapped to the two most important components (t1 and t2) of a PLS-DA classifier trained to classify the tissue sections into the three indicated classes. (Ellipses = Mahalanobis distances, R2Y = explained variances, Q2Y = prediction accuracies of the PLS-DA classifier, numbers after the class labels = tissue section numbers).

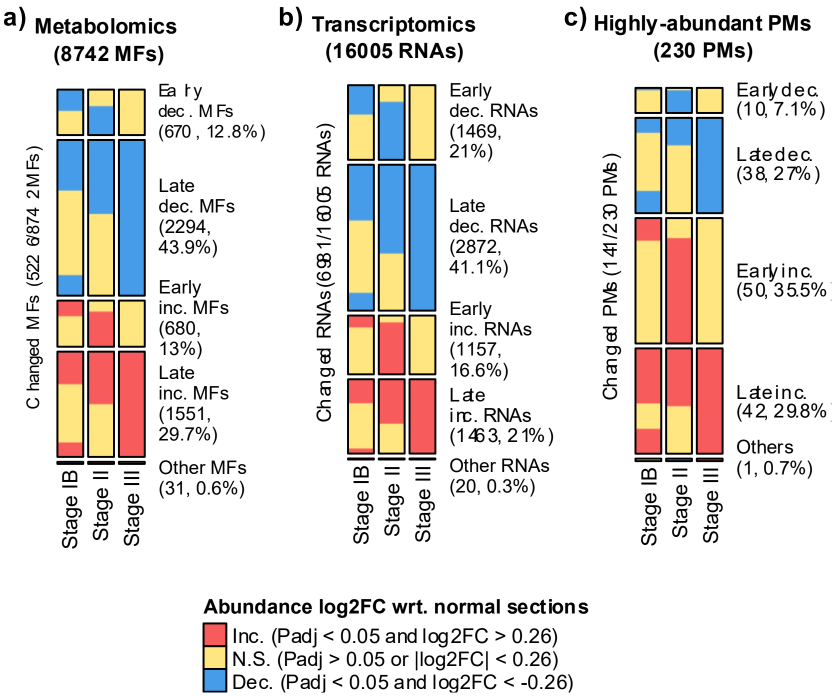

Appendix Fig. S4. Distributions of changed transcriptomic or metabolomic profiles across the three HCC stages. Stacked bar charts showing the percentages of **a)** mass features (MFs), **b)** RNAs, or **c)** highly abundant putative metabolites (PMs) with mean abundance levels in tumor sections significantly different from the mean abundance levels in adjacent-normal sections in at least one of the three HCC stages. (Two-side student t's or Wald's tests were used for LC-MS or RNA-seq/PM profiles, respectively; Padj = BH-adjusted P-values; Inc. = significantly increased, Padj < 0.05 and log2FC > 0.26; Dec. = significantly decreased, Padj < 0.05 and log2FC < -0.26; N.S. = not significantly changed, Padj > 0.05 or |log2FC| < 0.26).

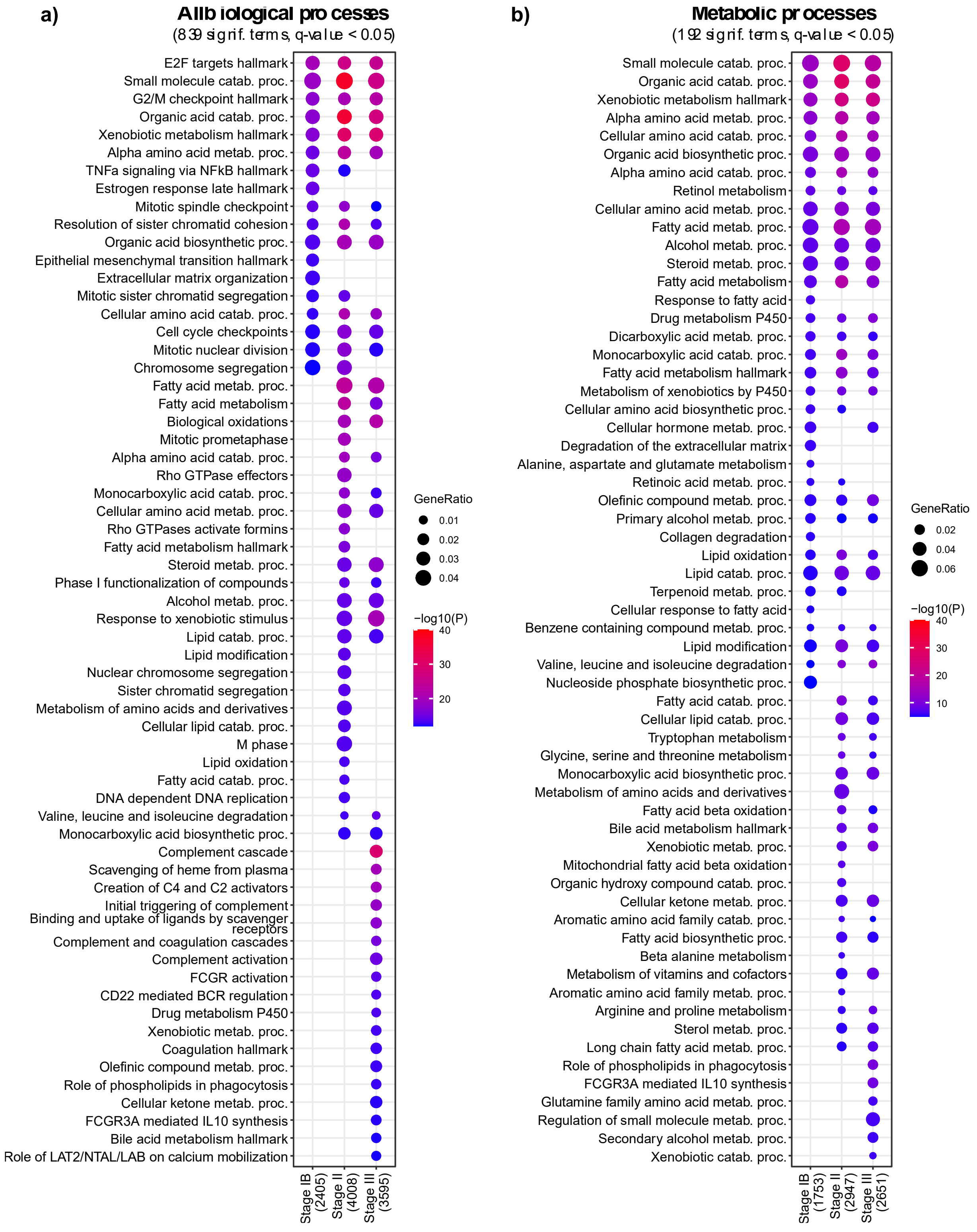

Appendix Fig. S5. Biological pathways and processes enriched in genes that are differentially expressed at different HCC stages. Dot plots showing the top significantly enriched or de-enriched (a) biological processes or (b) metabolic processes in the DEGs between adjacent-normal tissues and Stage 1B, II, or III tumor sections. (Two-sided hypergeometric tests; q-value = expected positive false discovery rate obtained from the Storey's procedure; numbers after the stage labels = DEG numbers).

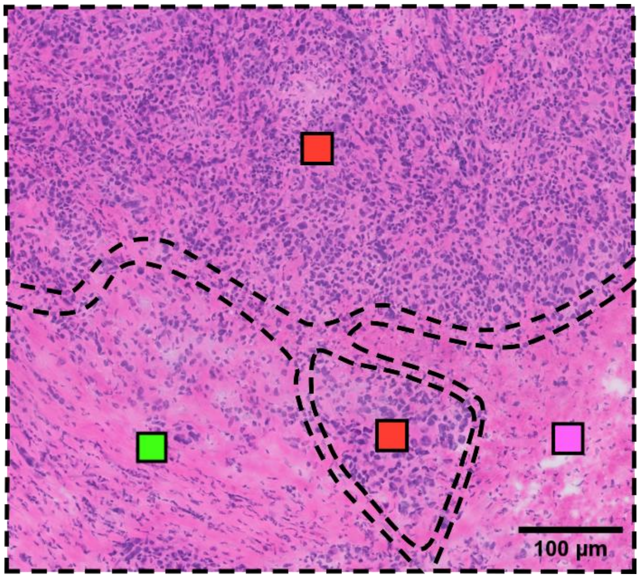

Appendix Fig. S6. Additional exemplary H&E image showing heterogeneous histological features within the same HCC tumor. The image was from patient HEP277's tumor (dashed lines = annotated histological regions; red regions = poorly differentiated hepatocytes; green regions = fibrosis; magenta regions = necrosis, respectively).

a) Support vector machine (linear kernel)

ME Region	Training average (%)				Test average (%)			
	AUC	Sensi.	Speci.	BAC	AUC	Sensi.	Speci.	BAC
Normal	100.0	99.3	99.6	99.5	99.5	95.8	96.1	96.0
G2	100.0	99.4	79.2	89.3	86.2	71.5	74.8	73.2
G3	99.7	56.6	99.8	78.2	95.4	50.2	95.5	72.8
Necrotic	99.8	93.7	99.8	96.7	96.0	78.5	97.9	88.2
Steatotic	98.9	78.0	98.2	88.1	92.6	60.0	97.6	78.8
Fibrotic	92.1	48.6	99.1	73.9	83.4	35.0	95.4	65.2

b) Support vector machine (radial-basis-function kernel)

ME Region	Training average (%)				Test average (%)			
	AUC	Sensi.	Speci.	BAC	AUC	Sensi.	Speci.	BAC
Normal	100.0	100.0	100.0	100.0	99.8	97.0	97.7	97.4
G2	100.0	100.0	95.8	97.9	88.7	81.0	83.2	82.1
G3	100.0	91.2	100.0	95.6	97.7	70.3	97.4	83.9
Necrotic	100.0	99.4	100.0	99.7	96.3	84.5	99.5	92.0
Steatotic	100.0	100.0	100.0	100.0	95.4	84.5	97.4	90.9
Fibrotic	99.6	82.8	99.1	91.0	86.2	46.0	97.7	71.8

Appendix Fig. S7. MER classification performances of the support vector machines. Tables showing the mean training and test performances of three MER classifiers based on support vector machines with **(a)** linear or **(b)** radial-basis-function kernels (AUC = area under the ROC curves; BAC = balanced accuracy). The values were estimated using a 10x10-fold cross validation procedure.

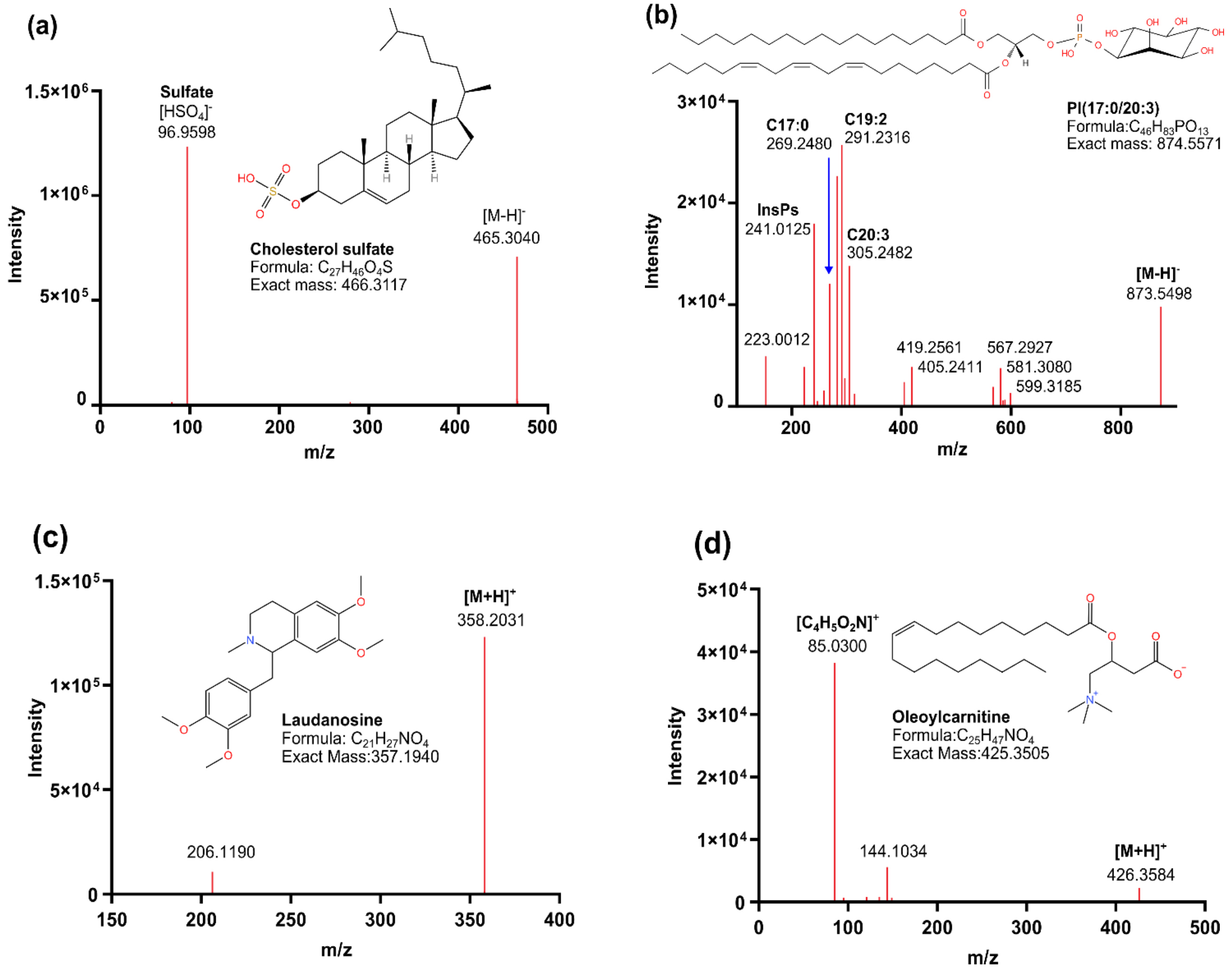

Appendix Fig. S8. MS/MS spectra and fragmentation patterns of the 11 manually annotated PMs. (a) MS/MS spectrum of N14 with high collision energy at 35 V. The mass feature 2.55_465.3040m/z $[M\text{-}H]^-$ was identified and annotated as cholesterol sulfate. The m/z 96.9598 corresponds to the sulfate ions. **(b)** MS/MS spectrum of N71 with high collision energy at 45 V. The mass feature 5.74_873.5498m/z $[M\text{-}H]^-$ was identified and annotated as PI(17:0/20:3). The m/z 223.0012, 241.0125 and 259.0227 correspond to inositol phosphate (InsPs) ions, which are characteristic for the polar head group. The m/z 297.0375 corresponds to InsPs with the glycerol backbone attached, which is characteristic of phosphatidylinositol. The m/z 269.2480 and 305.2482 correspond to the carboxylates of fatty acids (FA)17:0 and FA20:3, respectively. The m/z 567.2927 and 405.2411 represent the neutral losses of FA20:3 and followed by an additional loss of inositol from the hexose head fragment of 162.05. **(c)** MS/MS spectrum of P29 with medium collision energy at 20 V. The mass feature 2.88_358.2031m/z $[M+H]^+$ was identified and annotated as laudanosine, which is identically matched with the theoretical MS/MS spectra of HMDB0030213 in Drugbank. The m/z 206.1 is the protonated tetrahydroisoquinoline (isoquinolinium) core of laudanosine after benzylic cleavage. **(d)** MS/MS spectrum of P37 with medium collision energy at 30 V. The mass feature 1.94_426.3584m/z $[M+H]^+$ was identified and annotated as oleoylcarnitine. The m/z 85.0300 is diagnostic acylcarnitine fragment. This is the most widely used confirmatory fragment for acylcarnitines in positive mode.

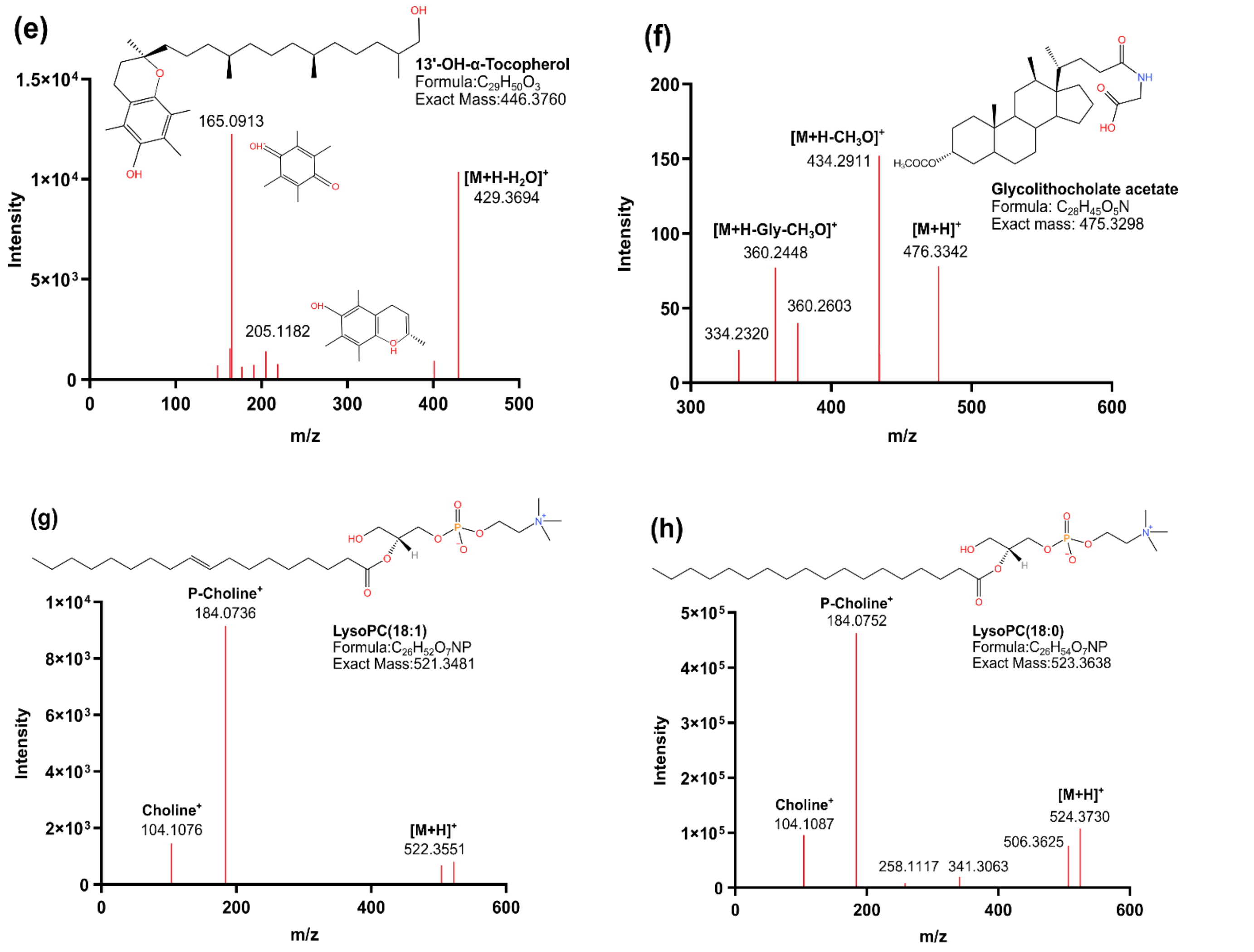

Appendix Fig. S8 (cont.) (e) MS/MS spectrum of P38 with medium collision energy at 30 V. The mass feature 5.12_429.3694m/z $[M+H-H_2O]^+$ was identified and annotated as 13'-OH-α-tocopherol. The m/z 165 due to a retro-Diels−Alder reaction and a minor product ion at m/z 205 due to an α-cleavage at site 2–1' **(f)** MS/MS spectrum of P42 with high collision energy at 35 V. The mass feature 3.30_476.3342 m/z $[M+H]^+$ was identified and annotated as glycolithocholate acetate. The m/z 434.2911 corresponds to the loss of an acetyl group while m/z 360.2448 corresponds to the subsequent loss of a glycine moiety. **(g)** MS/MS spectrum of P58 with medium collision energy at 20 V. The mass feature 4.93_522.3551 m/z $[M+H]^+$ was identified and annotated as LysoPC(18:1). The m/z 184.0736 and 104.1076 correspond to the phosphocholine ions (P-Choline) and choline ions respectively. **(h)** MS/MS spectrum of P60 with medium collision energy at 25 V. The mass feature 2.43_524.3730 m/z $[M+H]^+$ was identified and annotated as LysoPC(18:0). The m/z 184.0752 and 104.1087 correspond to the phosphocholine ions and choline ions respectively.

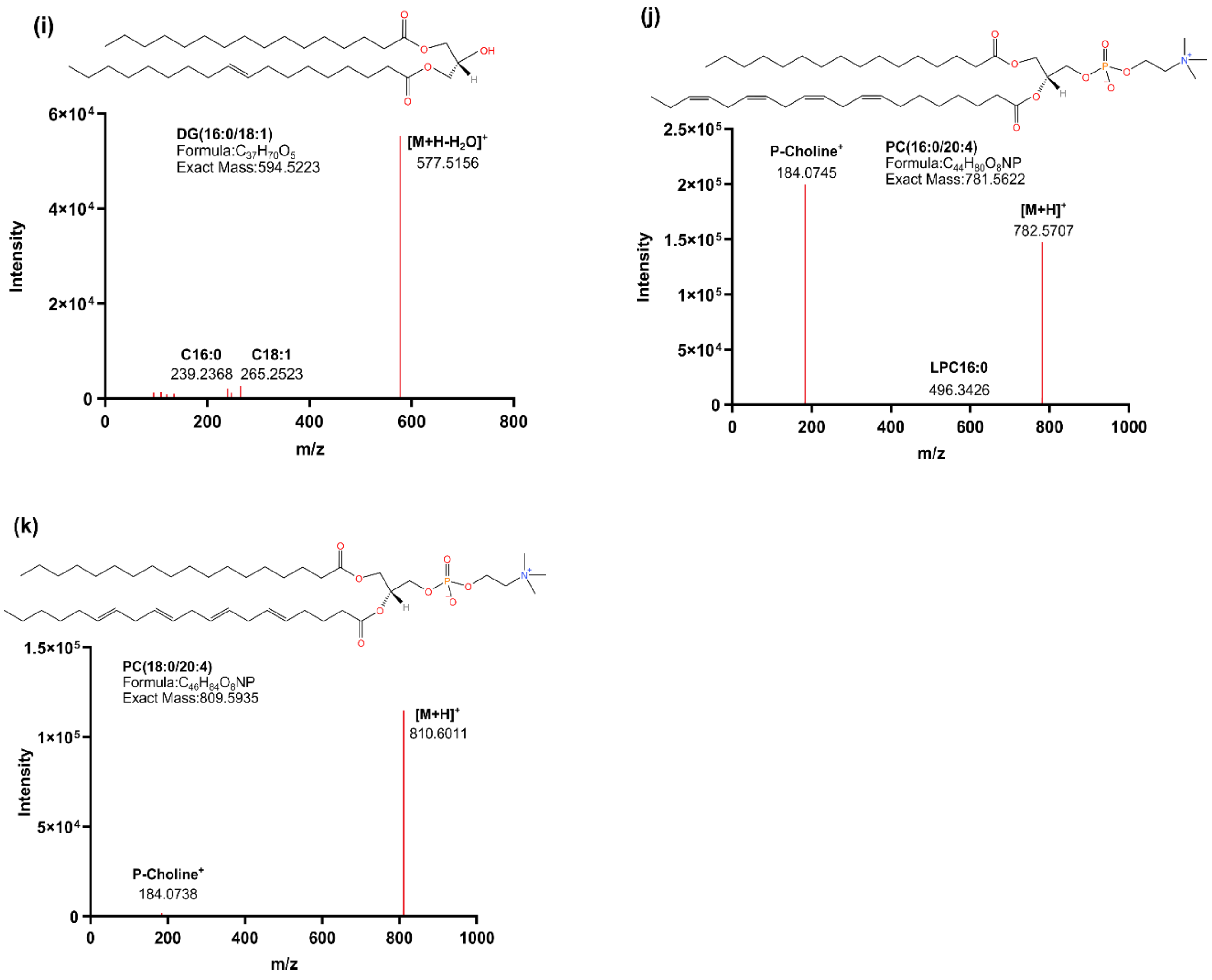

Appendix Fig. S8 (cont.) (i) MS/MS spectrum of P73 with medium collision energy at 25 V. The mass feature 10.92_577.5156 m/z $[M+H-H_2O]^+$ was identified and annotated as DG(16:0/18:1). **(j)** MS/MS spectrum of P120 with medium collision energy at 25 V. The mass feature 4.00_782.5707 m/z $[M+H]^+$ was identified and annotated as PC(16:0/20:4). The m/z 184.0745 corresponds to the phosphocholine ions. The m/z 496.3426 corresponds to LPC 16:0 $[M+H_2O+H]^+$ after a neutral loss of FA 20:4. **(k)** MS/MS spectrum of P135 with medium collision energy at 30 V. The mass feature 3.99_810.6011m/z $[M+H]^+$ was identified and annotated as PC(18:0/20:4). The m/z 184.0738 corresponds to the phosphocholine ions.

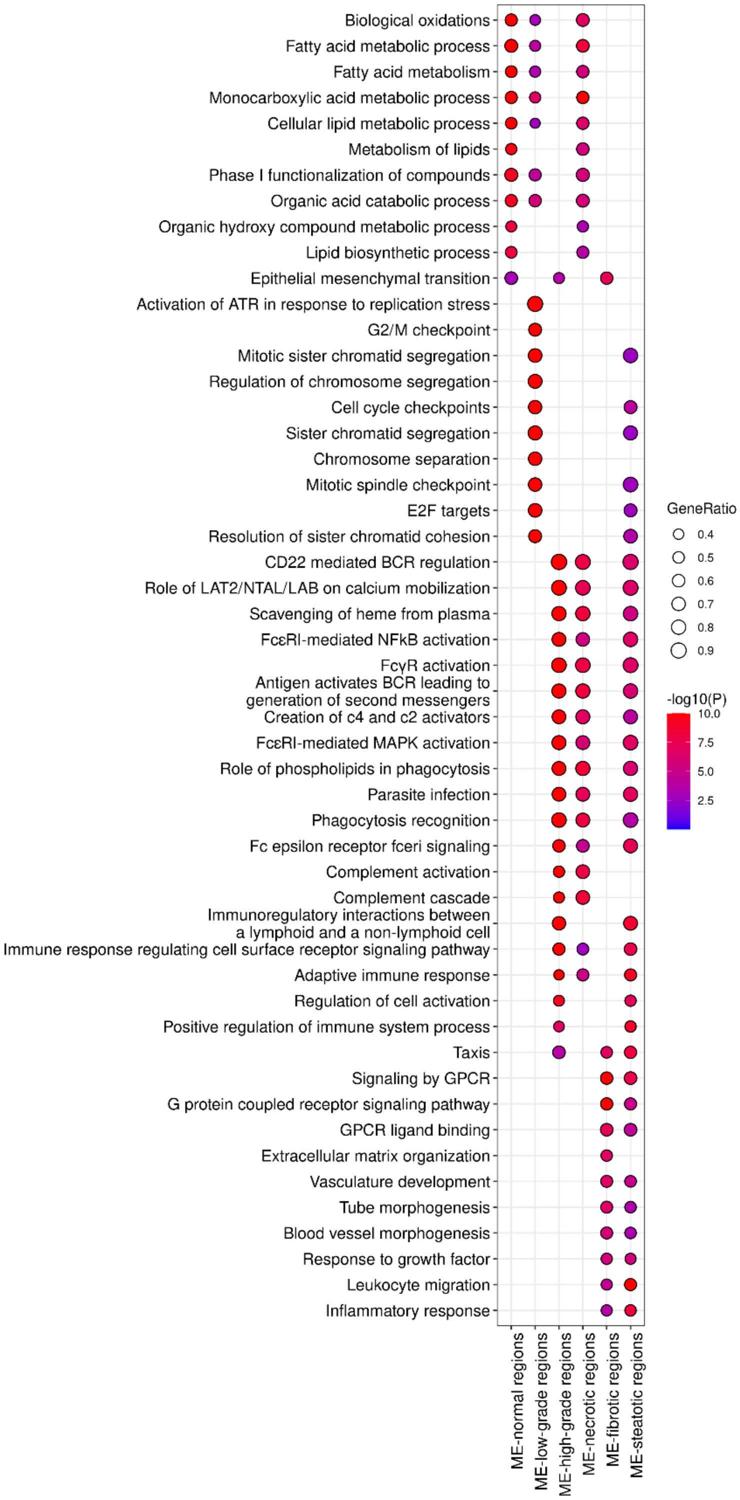

Appendix Fig. S9. Biological pathways and processes enriched in RNAs correlated to the discriminative PMs for different MERs. Dot plots showing significantly enriched biological pathways and processes found using Gene Set Enrichment Analysis (GSEA) in RNAs ranked according to their absolute rank correlations to the discriminative PMs for different MERs (Padj < 0.05, BH-adjusted P-values).

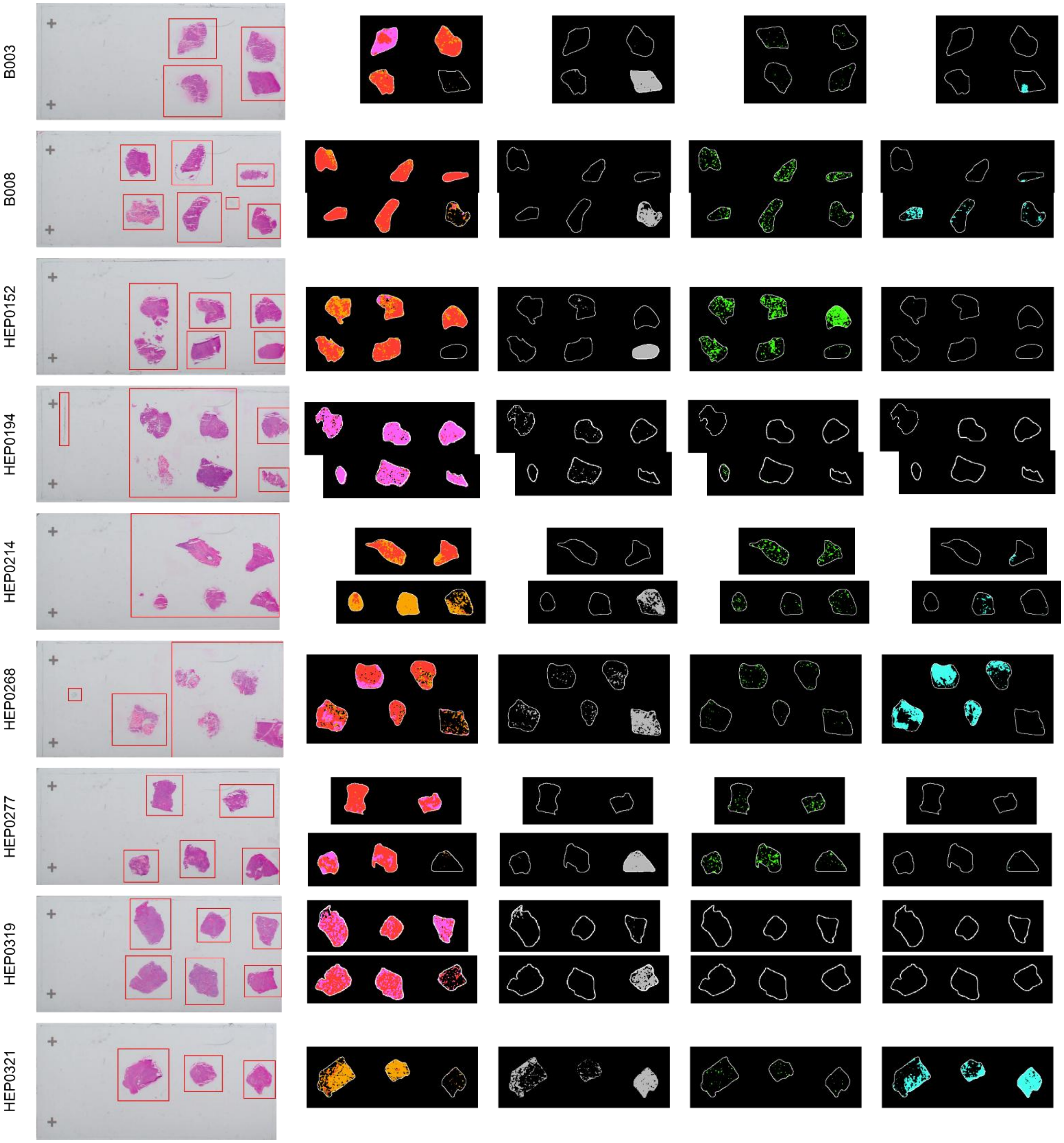

Appendix Fig. S10. H&E images and SgME maps for the tissue sections collected from nine HCC patients. Each pixel of the SgME map was assigned to one of the ME-transformed regions (normal, low-grade, high-grade, or necrotic), ME-fibrotic regions (non-fibrotic, or fibrotic), and ME-steatotic regions (non-steatotic or steatotic). The H&E image of HEP0152 is the same H&E image in Fig. 3d and the SgME maps for HEP0152 and B003 are the same SgME maps in Fig. 6a. (White lines = annotated tissue boundaries; Red = high-grade, orange = low-grade, grey = normal, magenta = necrotic, green = fibrotic and cyan = steatotic regions, respectively).

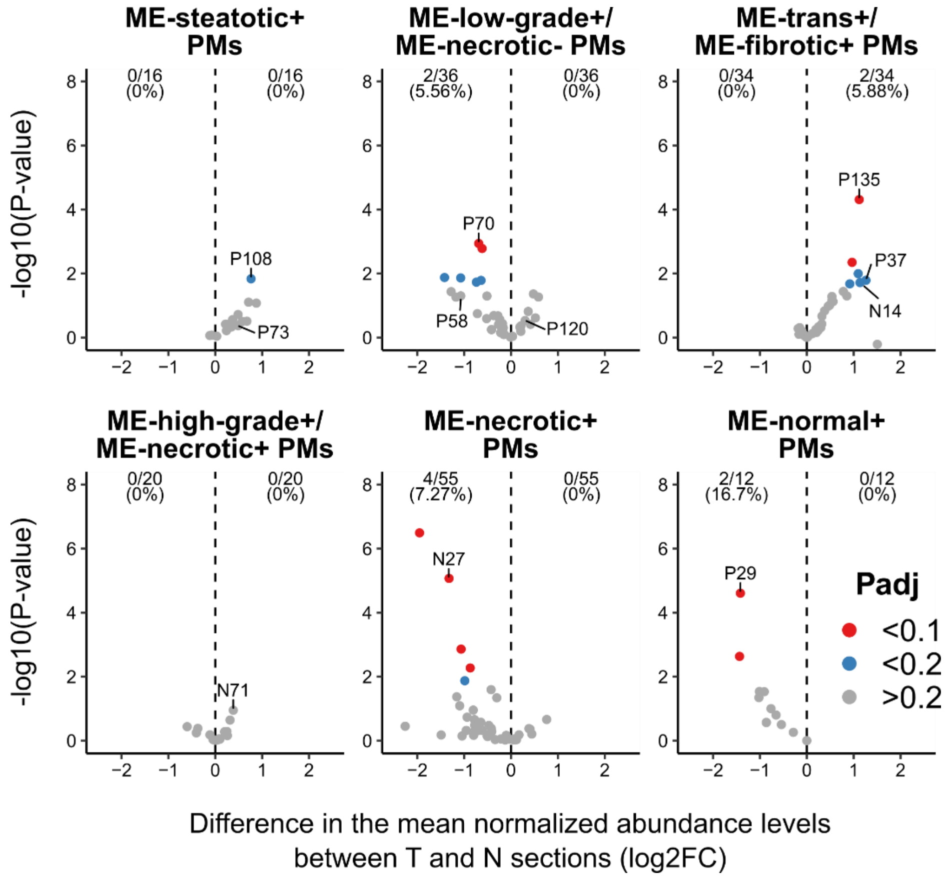

Appendix Fig. S11. Significantly changed PMs from the six PM clusters. Volcano plot showing highly abundant PMs categorized according to the six PM clusters and with significantly changed averaged tissue abundance levels between the adjacent normal tissue and tumor sections (two-sided t-test, Padj = BH-adjusted P values).

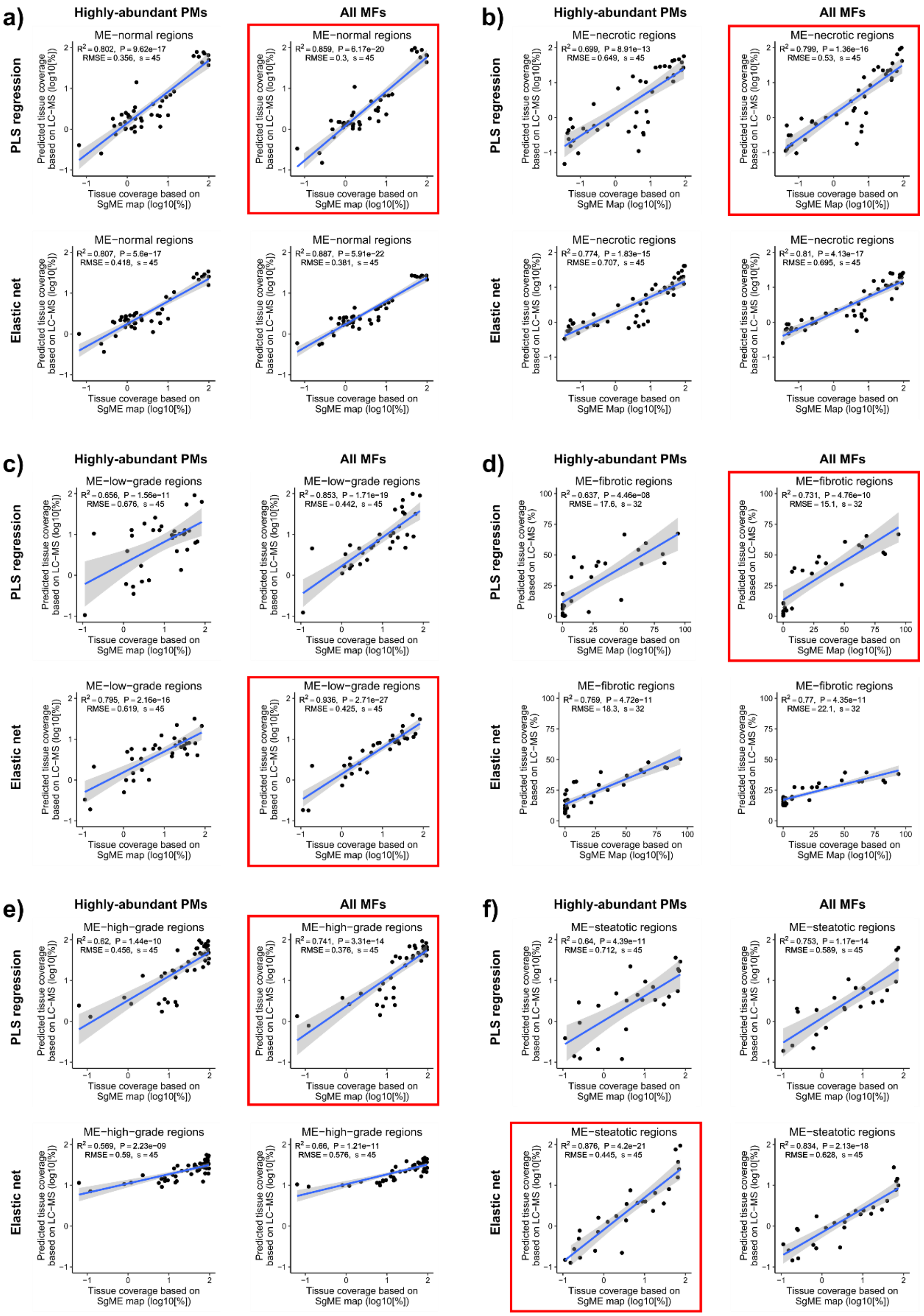

Appendix Fig. S12. Performances of all the tested regression models for SgMERdeconv. Scatter plots showing the predicted MER tissue coverages based on LC-MS profiles versus the measured MER tissue coverages based on the SgME maps. The optimum regression model for each MER is highlighted by a red box (R^2 = coefficients of determination, RMSE = root mean square errors of the regression models).

Appendix Table S1: Clinical information for all the 26 patients studied

Patient ID	Gender	Ethnicity	Histo diagnosis	TNM stage (AJCC V8)	Max diameter of tumor (cm)	Multifocality based on histo	Child's Pugh Score	Micro-vascular invasion	R0 or R1	Edmondson-Steiner Grading	Fibro-sis score	Steato-sis in %#	Necro-sis in %	Total no. of sectors	No. of adj normal sectors	No. of tumor sectors	LC-MS	DESI-MSI	RNA-Seq	STx
B002	Male	Chinese	HCC	Stage IB	2.5	Solitary	A	Yes	R0	2	0	10	5	3	1	2	Y	N	Y	N
B003	Male	Chinese	HCC	Stage IIIB	11	Solitary	A	Yes	R0	3	3	<1	35	4	1	3	Y	Y	Y	N
B008	Male	Chinese	HCC	Stage IIIB	7	Solitary	A	No	R0	3	3	<1	20	6	1	5	Y	Y	Y	N
B014	Male	Chinese	HCC	Stage II	14	Solitary	A	No	R0	2	3	0	40	6	1	5	Y	N	Y	N
B015	Male	Chinese	HCC	Stage IB	2.2	Solitary	A	No	R0	2	4	15	5	3	1	2	Y	N	Y	N
C006	Male	Chinese	HCC	Stage IB	24	Solitary	A	No	R0	2	0	0	NA	6	1	5	Y	N	Y	N
F008	Male	Filipino	HCC	Stage II	13	Solitary	NA	No	R0	4	3	20	0	1	0	1	N	N	N	Y
F011	Male	Filipino	HCC	Stage IB	7	Solitary	A	No	R0	1	1	20	0	1	0	1	N	N	N	Y
HEP0026	Male	Indones-ian Chinese	HCC	Stage II	14	Solitary	A	No	R0	3	0	0	5	7	1	6	Y	N	Y	N
HEP0121	Male	Chinese	HCC	Stage II	3.2	Solitary	A	No	R1	3	3	0	5	3	0	3	Y	N	N	N
HEP0152	Male	Chinese	HCC	Stage IIIA	14, 0.2	Multifocal	A	Yes	R0	3	0	0	10	6	1	5	Y	Y	Y	N
HEP0194	Female	Chinese	HCC	Stage IB	7	Solitary	A	No	R0	3	4	<5	10	6	1	5	Y	Y	Y	N
HEP0198	Female	Chinese	HCC	Stage II	4	Solitary	A	Yes	R0	2	NA	NA	0	5	1	4	Y	N	N	N
HEP0206	Female	Chinese	HCC	Stage IB	7	Solitary	A	Yes	R0	3	3	<1	10	3	1	2	Y	N	Y	N
HEP0209	Female	Chinese	HCC	Stage IB	4.8	Solitary	A	Yes	R0	2	0	0	0	6	1	5	Y	N	Y	N
HEP0214	Male	Malay	HCC	Stage IB	6.5	Solitary	A	Yes	R0	2	4	NA	40	7	1	6	Y	Y	N	N
HEP0229	Male	Chinese	HCC	Stage IIIB	8.5, 1.0, 0.6, 0.6	Multifocal	A	Yes	R0	3	0	<5	5 (Seg 4B & 6)<br>0 (Seg 4B)<br>5 (Seg 6, 1 cm)<br>0 (Seg 6, 0.6 cm)	6	1	5	Y	N	Y	N
HEP0261	Male	Malay	HCC	Stage II	3	Multifocal	A	Yes	R0	3	4	10	<1	3	1	2	Y	N	Y	N
HEP0262	Male	Chinese	HCC	Stage IIIA	15, 5	Multifocal	A	No	R0	3	3	5	10 (Left)<br>0 (Seg 5)	5	1	4	Y	N	Y	N
HEP0268	Female	Chinese	HCC	Stage IIIB	14	Solitary	A	Yes	R1	4	1	0	90	5	1	4	Y	Y	N	N
HEP0275	Female	Chinese	CCA	Stage IB	3	Solitary	A	Yes	R0	2	0	NA	0	3	1	2	Y	Y	N	N
HEP0276	Male	Chinese	HCC	Stage IB	5.5	Solitary	A	Yes	R0	3	3	20	20	3	1	2	Y	Y	Y	N

HEP0277	Male	Chinese	HCC	Stage II	7	Solitary	A	No	R0	3	2	<1	40	5	1	4	Y	Y	Y	N
HEP0290	Female	Chinese	CCA	Stage IB	5.2	Solitary	A	Yes	R0	2	0	NA	10	5	1	4	Y	N	N	N
HEP0319	Male	Indonesian	HCC	Stage IIIB	5.6	Solitary	A	No	R0	3	1	10	<1	6	1	5	Y	Y	Y	N
HEP0321	Male	Chinese	HCC	Stage IB	2.5	Solitary	A	No	R1	2	4	15	0	3	1	2	Y	Y	Y	N

*As of Aug 2022
#<5%=0; 5-33%=1; >33-66%=2; >66%=3

Appendix Table S2: Number of tissue sections analyzed using different spatial and/or non-spatial omics technologies in our study

Case ID	Section ID	LC-MS	DESI-MSI	RNA-seq	STx
B002	N	Y		Y	
B002	T1	Y		Y	
B002	T2	Y		Y	
B003	N	Y	Y	Y	
B003	T1	Y	Y	Y	
B003	T2	Y	Y	Y	
B003	T5	Y	Y	Y	
B008	N	Y	Y	Y	
B008	T1	Y	Y	Y	
B008	T2	Y	Y	Y	
B008	T3	N*	Y	Y	
B008	T4	Y	Y	Y	
B008	T5	Y	Y	Y	
B014	N	Y		Y	
B014	T1	Y		Y	
B014	T2	Y		Y	
B014	T3	N*		Y	
B014	T4	Y		Y	
B014	T5+	Y			
B015	N	Y		Y	
B015	T1	Y		Y	
B015	T2	Y		Y	
C006	N	Y		Y	
C006	T1	Y		Y	
C006	T2	Y		Y	
C006	T3	Y		Y	
C006	T4	Y		Y	
C006	T5	Y		Y	
F008	T1				Y
F011	T1				Y
HEP0026	N			Y	
HEP0026	T1	Y		Y	
HEP0026	T2	Y			
HEP0026	T3	Y		Y	
HEP0026	T5			Y	
HEP0026	T6			Y	
HEP0026	T7			Y	
HEP0121	T1	Y			
HEP0121	T2	Y			
HEP0121	T3	Y			
HEP0152	N	Y	Y		
HEP0152	T1	Y	Y	Y	
HEP0152	T10	Y	Y	Y	
HEP0152	T13	Y	Y	Y	
HEP0152	T4	Y	Y	Y	
HEP0152	T7	Y	Y	Y	
HEP0194	N	Y	Y	Y	
HEP0194	T1	Y	Y	Y	
HEP0194	T2	Y	Y	Y	
HEP0194	T3	N*	Y	Y	
HEP0194	T4	Y	Y	Y	
HEP0194	T5	Y	Y		
HEP0198	N	Y			
HEP0198	T2	Y			
HEP0198	T3	Y			
HEP0198	T4	Y			
HEP0198	T5+	Y			
HEP0206	N	Y			
HEP0206	T3	N*		Y	
HEP0206	T4	Y		Y	
HEP0209	N	Y		Y	
HEP0209	T1	Y			
HEP0209	T2	Y			
HEP0209	T3			Y	
HEP0209	T4			Y	
HEP0209	T5+	Y		Y	
HEP0214	N	Y	Y		
HEP0214	T1	Y			
HEP0214	T2	Y	Y		
HEP0214	T3	Y	Y		
HEP0214	T4	Y			
HEP0214	T5	Y	Y		
HEP0214	T6	Y	Y		

HEP0229	N	Y		Y	
HEP0229	T1	Y		Y	
HEP0229	T2	Y		Y	
HEP0229	T3	Y		Y	
HEP0229	T4	Y		Y	
HEP0229	T5	Y		Y	
HEP0261	N	Y		Y	
HEP0261	T1	Y		Y	
HEP0261	T2	Y		Y	
HEP0262	N	Y		Y	
HEP0262	T1	Y		Y	
HEP0262	T2	Y		Y	
HEP0262	T3	Y		Y	
HEP0262	T4	Y		Y	
HEP0268	N	Y	Y		
HEP0268	T1	Y	Y		
HEP0268	T3	Y	Y		
HEP0268	T4	Y	Y		
HEP0268	T5	Y	Y		
HEP0275	N	Y	N*		
HEP0275	T1	Y	N*	Y	
HEP0275	T2	Y	N*		
HEP0276	N	Y	N*	Y	
HEP0276	T2	Y	N*	Y	
HEP0276	T3	Y	N*	Y	
HEP0277	N	Y	Y	Y	
HEP0277	T1	Y	Y	Y	
HEP0277	T2	Y	Y	Y	
HEP0277	T3	Y	Y	Y	
HEP0277	T4	Y	Y	Y	
HEP0290	N	Y			
HEP0290	T1	Y			
HEP0290	T2	Y			
HEP0290	T3	Y			
HEP0290	T5+	Y			
HEP0319	N	Y	Y	Y	
HEP0319	T1	Y	Y	Y	
HEP0319	T2	Y	Y	Y	
HEP0319	T3	N*	Y	Y	
HEP0319	T4	Y	Y	Y	
HEP0319	T5	Y	Y	Y	
HEP0321	N	N*	Y	Y	
HEP0321	T1	Y	Y	Y	
HEP0321	T2	Y	Y	Y	
**Total**	**117**	**109**	**52**	**81**	**2**
**Analyzed**		**103**	**46**	**81**	**2**

Reasons for exclusion from DESI-MSI analysis (N*)
HEP0275 CCA, non-HCC
HEP0276 CPP abundance levels too low

Reasons for exclusion from LCMS analysis (N*)
B014 - T3 Only had usable mass spectra for one of the three ionization modes
B008 - T3 Only had usable mass spectra for two of the three ionization modes
HEP0319 - T3 Only had usable mass spectra for two of the three ionization modes
HEP0194 - T3 Only had usable mass spectra for two of the three ionization modes
HEP0206 - T3 Only had usable mass spectra for two of the three ionization modes
HEP0321 - N Only had usable mass spectra for two of the three ionization modes

Appendix Table S3: List of highly-abundant putative metabolites found and their information

peak_id	mz_id	lcms_mode	ion_type	iqr	mean	med	pct90	MSMS_annotation	mz	exact_mz
N1	2.91_256.2407n	LNEG	neg	0.84060	24.81403	24.72631	25.40843	Palmitic acid	255.23	256.24
N10	2.47_328.2404n	LNEG	neg	1.69214	22.25109	22.16122	23.80283	Docosahexaenoic acid	327.23	328.24
N11	6.12_327.2332m/z	LNEG	neg	1.45523	23.21906	23.30260	24.39702	Retinol acetate	327.23	328.24
N12	1.79_339.2003m/z	LNEG	neg	0.65524	23.82551	23.76020	24.48638	13,14-Dihydro PGF-1a	339.20	358.27
N13	1.99_452.2786m/z	LNEG	neg	1.43330	23.68487	23.77055	24.86241	LysoPE(0:0/16:0)	452.28	453.29
N14	2.67_465.3048m/z	LNEG	neg	2.59211	25.82590	25.55268	27.96367	Cholesterol sulfate	465.30	466.31
N15	2.14_478.2942m/z	LNEG	neg	2.11380	22.88375	22.49516	24.85979	LysoPE(18:1/0:0)	478.29	479.30
N16	2.56_480.3097m/z	LNEG	neg	1.15955	24.63163	24.59184	25.45542	LysoPE(0:0/18:0)	480.31	481.32
N17	5.79_687.5444m/z	LNEG	neg	0.77224	24.87065	24.78109	25.52861	Unannotated	687.54	NA
N18	6.16_667.5176n	LNEG	neg	1.45136	24.40327	24.26243	25.88330	PC(14:1/15:0)	688.49	689.50
N19	7.21_700.5269m/z	LNEG	neg	1.13683	24.03179	23.99428	25.53816	PE(16:0/18:0)	700.53	719.55
N2	2.62_279.2332m/z	LNEG	neg	1.25870	23.35709	23.18973	25.18241	Linoleic acid	279.24	280.24
N20	6.31_701.5124m/z	LNEG	neg	0.97296	25.08115	25.04880	25.91117	CerP(d18:1/22:0)	701.51	701.57
N21	6.31_714.5068m/z	LNEG	neg	1.36174	26.09644	26.14119	27.33239	PE(16:0/18:2)	714.51	715.52
N22	5.97_716.5228m/z	LNEG	neg	1.13842	24.94058	24.94942	25.99474	PE(16:0/18:1)	716.52	717.53
N23	6.84_716.5221m/z	LNEG	neg	0.97997	26.56031	26.62550	27.36778	PE(16:0/18:1)	716.52	717.53
N24	6.67_722.5115m/z	LNEG	neg	0.68141	26.76838	26.86640	27.42180	PE(20:4/P-16:0)	722.51	723.52
N25	6.66_728.5243m/z	LNEG	neg	1.50630	23.58862	23.31882	25.64709	PC(16:1/16:1)	728.52	729.53
N26	7.83_728.5589m/z	LNEG	neg	1.02371	24.61410	24.54743	25.69044	PE(20:0/16:0)	728.56	747.58
N27	6.29_738.5067m/z	LNEG	neg	0.60114	26.83581	26.93941	27.44129	PE(18:1/18:3)	738.51	739.52
N28	6.15_742.5378m/z	LNEG	neg	0.90858	26.54734	26.69186	27.22757	PE(18:0/18:2)	742.54	743.55
N29	7.01_742.5373m/z	LNEG	neg	1.03340	27.38090	27.39228	28.32308	PE(18:0/18:2)	742.54	743.55
N3	3.06_282.2561n	LNEG	neg	2.09359	25.14574	25.08589	27.49385	Oleic acid	281.25	282.26
N30	7.47_744.5530m/z	LNEG	neg	0.54579	26.37008	26.42561	26.79854	PE(20:0/16:1)	744.56	745.56
N31	6.66_744.5534m/z	LNEG	neg	0.81284	26.45314	26.43373	27.23825	PE(20:0/16:1)	744.56	745.56
N32	6.46_746.5109m/z	LNEG	neg	1.37668	24.31487	24.42114	25.66824	PE(22:6/P-16:0)	746.54	747.52
N33	5.36_747.5135m/z	LNEG	neg	1.49550	23.88528	23.82385	25.45276	PG(16:0/18:1)	747.51	748.53
N34	5.69_747.5110m/z	LNEG	neg	0.52921	26.63510	26.63657	27.16909	PG(16:0/18:1)	747.52	748.53
N35	7.33_750.5423m/z	LNEG	neg	0.79563	26.21391	26.24500	27.06507	PE(18:0/20:3)	750.54	769.56
N36	7.10_750.5428m/z	LNEG	neg	0.98829	24.56646	24.64349	25.37666	PE(18:0/20:3)	750.54	769.56
N37	6.64_752.5260m/z	LNEG	neg	1.23206	23.99842	23.88715	25.18302	PE(22:4/15:0)	752.52	753.53
N38	7.54_752.5584m/z	LNEG	neg	1.20900	23.05398	22.93735	23.98047	PC(15:0/20:2)	752.56	771.58
N39	6.13_762.5070m/z	LNEG	neg	1.97925	27.80014	27.66576	29.19473	PE(16:0/22:6)	762.51	763.52
N4	3.64_284.2717n	LNEG	neg	0.57184	24.77314	24.63788	25.52354	Stearic acid	283.27	284.27
N40	6.99_766.5369m/z	LNEG	neg	0.73803	28.52471	28.64471	29.23322	PE(18:0/20:4)	766.54	767.55
N41	6.14_766.5363m/z	LNEG	neg	0.79586	25.42666	25.41599	26.21382	PE(18:0/20:4)	766.54	767.55
N42	7.42_768.5531m/z	LNEG	neg	2.64103	23.93247	23.84012	25.67179	PC(15:0/20:3)	768.55	769.56
N43	7.17_768.5530m/z	LNEG	neg	0.81766	25.23525	25.36287	26.03401	PC(15:0/20:3)	768.55	769.56

N44	6.83_770.5693m/z	LNEG	neg	0.70334	25.82401	25.84252	26.43103	PE(18:0/20:2)	770.57	771.58
N45	7.31_772.5845m/z	LNEG	neg	0.97516	24.46932	24.28330	25.37756	CerP(d18:1/24:1)	772.59	727.59
N46	5.51_773.5321m/z	LNEG	neg	1.14959	27.27574	27.34267	28.32109	PG(18:0/18:2)	773.53	774.54
N47	5.93_752.5586n	LNEG	neg	0.72196	25.40824	25.31019	26.08649	PG(18:0/18:2)	773.53	774.54
N48	7.12_774.5427m/z	LNEG	neg	0.83062	25.06086	25.00152	26.19338	PE(22:6/P-18:0)	774.54	775.55
N49	6.36_775.5481m/z	LNEG	neg	1.08968	24.87295	24.76662	25.84881	PG(18:0/18:1)	775.55	776.56
N5	6.99_304.2393n	LNEG	neg	0.76864	23.84442	23.96387	24.58126	Arachidonic acid	303.23	304.24
N50	7.94_778.5745m/z	LNEG	neg	1.11726	23.69521	23.71935	24.96315	PE(20:0/20:3)	778.57	797.59
N51	7.72_778.5736m/z	LNEG	neg	1.14846	24.99964	24.96519	25.86319	PE(20:0/20:3)	778.58	797.59
N52	6.32_789.5444n	LNEG	neg	0.49217	27.10424	27.17726	27.59478	PS(18:0/18:1)	788.54	789.55
N53	6.80_790.5378m/z	LNEG	neg	1.61211	27.13144	27.27632	28.26745	PE(18:0/22:6)	790.54	791.55
N54	5.95_791.5409m/z	LNEG	neg	1.29907	23.40623	23.32743	24.59327	d-Tocotrienol	791.54	396.30
N55	7.22_792.5532m/z	LNEG	neg	1.66135	24.21833	24.20720	25.64468	PE(18:0/22:5)	792.56	793.56
N56	6.82_794.5677m/z	LNEG	neg	0.75579	24.22704	24.23479	25.19949	PE(18:0/22:4)	794.57	795.58
N57	7.87_797.6529m/z	LNEG	neg	0.69224	24.89908	24.87160	25.58654	TG(14:0/18:4/16:0)	797.65	798.67
N58	7.93_759.5416n	LNEG	neg	1.66245	22.75712	22.54661	24.45025	PE(24:0/18:4)	804.59	823.61
N59	5.85_810.5283m/z	LNEG	neg	0.73497	26.02286	26.06747	26.76565	PS(20:3/18:1)	810.53	811.54
N6	2.58_303.2332m/z	LNEG	neg	1.41578	22.54979	22.43715	24.36111	8-HETrE	303.23	322.25
N60	6.03_812.5444m/z	LNEG	neg	0.96942	24.58869	24.50297	25.40806	PS(18:0/20:3)	812.54	813.55
N61	4.87_819.5173m/z	LNEG	neg	2.04718	25.30592	25.49060	26.91471	PG(18:1/22:6)	819.52	820.53
N62	8.47_832.6209m/z	LNEG	neg	1.69418	22.79093	22.62869	24.64564	PE(24:0/20:4)	832.62	851.64
N63	5.09_833.5182m/z	LNEG	neg	1.35264	25.56889	25.78560	27.09916	PI(16:0/18:2)	833.52	834.53
N64	5.65_834.5287m/z	LNEG	neg	0.72610	27.24542	27.36851	28.09262	PE(22:6/22:6)	834.53	835.52
N65	6.09_836.5450m/z	LNEG	neg	1.07451	24.03150	23.90797	25.32689	PE(22:5/18:1)	836.54	791.55
N66	5.11_857.5181m/z	LNEG	neg	1.14563	26.22695	26.23979	27.38122	PI(18:1/18:3)	857.52	858.53
N67	5.24_859.5345m/z	LNEG	neg	1.31699	25.25951	25.22477	26.78057	PI(18:3/18:0)	859.54	860.54
N68	5.74_861.5499m/z	LNEG	neg	1.06490	26.56009	26.50962	28.31520	PI(18:2/18:0)	861.55	862.56
N69	6.27_842.5903n	LNEG	neg	1.00930	27.33903	27.39581	28.35572	Latanoprost	863.56	432.29
N7	2.82_306.2583n	LNEG	neg	1.39362	20.25477	20.09634	21.61337	8,11,14-Eicosatrienoic acid	305.25	306.26
N70	4.26_865.5027m/z	LNEG	neg	2.76310	23.22897	23.30766	25.44019	Unannotated	865.50	NA
N71	5.93_874.5575n	LNEG	neg	3.52292	21.49867	21.44980	23.90803	PI(17:0/20:3)	873.55	873.55
N72	4.95_881.5191m/z	LNEG	neg	1.75639	24.30516	24.37415	25.78529	Amitenone	881.52	836.52
N73	5.78_885.5490m/z	LNEG	neg	0.61055	28.59860	28.67760	29.19601	PI(18:0/20:4)	885.55	886.56
N74	6.00_888.5775n	LNEG	neg	0.74037	27.12605	27.14134	27.76790	PI(16:0/22:3)	887.56	888.57
N75	7.06_888.6213m/z	LNEG	neg	1.10923	18.50797	17.95426	19.64710	3-O-Sulfogalactosylceramide (d18:1/24:1)	888.57	889.63
N76	6.90_888.6217m/z	LNEG	neg	1.89536	20.59400	20.44647	22.95310	3-O-Sulfogalactosylceramide (d18:1/24:1)	888.62	889.63
N77	6.59_889.5806m/z	LNEG	neg	3.94185	21.89679	21.88184	24.75350	DHAP(O-18:0)	889.58	422.28
N78	6.47_889.5810m/z	LNEG	neg	1.95869	22.72097	22.69304	25.17768	DHAP(O-18:0)	889.58	422.28
N79	5.61_909.5498m/z	LNEG	neg	0.98190	26.43835	26.67635	27.35000	PI(18:0/22:6)	909.55	910.56
N8	1.29_311.1689m/z	LNEG	neg	1.04211	23.78625	23.83433	24.71730	THPGG	311.17	330.13
N80	6.06_911.5662m/z	LNEG	neg	0.90659	24.52523	24.63911	25.59158	TG(16:0/20:0/20:3)	911.57	912.81
N81	6.20_913.5813m/z	LNEG	neg	1.21161	24.53568	24.64779	25.48546	PI(18:0/22:4)	913.58	914.59
N82	7.06_1186.7442n	LNEG	neg	1.07853	23.50230	23.40334	24.34768	CL(8:0/10:0/18:2/18:2)	1185.74	1204.75

N83	7.43_1188.7706n	LNEG	neg	0.87575	22.83201	22.67829	23.65474	CL(8:0/10:0/18:1/18:2)	1187.75	1188.77
N9	1.54_325.1846m/z	LNEG	neg	0.85337	24.38316	24.35736	25.19654	Hydroxyhexamide	325.18	326.13
P1	5.00_85.0299m/z	HPOS	pos	1.38187	20.70840	21.65543	22.82661	Unannotated	85.03	NA
P10	8.13_183.0690n	LPOS	pos	0.31729	23.45550	23.40000	23.89231	Unannotated	184.07	NA
P100	2.80_696.4374n	LPOS	pos	0.89794	23.29231	23.33907	24.01792	Unannotated	719.43	NA
P101	4.57_702.5620n	HPOS	pos	1.11265	22.28660	23.17977	24.41995	SM(d18:0/16:1)	725.56	702.57
P102	4.08_705.5307n	HPOS	pos	2.59617	20.56104	21.69752	23.40866	PC(19:0/11:0)	728.52	705.53
P103	4.03_732.5549m/z	HPOS	pos	2.27265	22.71822	23.94148	25.18864	PC(14:0/18:1)	732.55	731.55
P104	6.26_731.5477n	LPOS	pos	1.15181	27.10226	27.12670	27.96154	PC(15:0/17:1)	732.56	731.55
P105	6.58_745.5665n	LPOS	pos	1.52706	24.24590	24.08522	25.97125	PE(18:1/18:0)	746.57	745.57
P106	4.02_745.5764n	HPOS	pos	2.95535	19.48832	20.61098	22.43358	PC(O-17:0/20:4)	746.58	781.60
P107	7.30_745.6002n	LPOS	pos	1.56866	23.57499	23.47740	24.90473	PC(16:0/P-18:0)	746.61	745.60
P108	6.99_748.5876m/z	LPOS	pos	2.03460	21.03706	21.10700	22.84692	PC(15:0/18:0)	748.59	747.58
P109	5.69_729.5338n	LPOS	pos	2.22683	20.44029	20.22086	22.17492	Unannotated	752.52	NA
P11	4.00_184.0745m/z	HPOS	pos	1.78797	23.54512	24.73071	25.70362	Phosphocholine	184.07	184.07
P110	2.90_737.4970n	HPOS	pos	3.22462	19.71232	21.32106	22.90659	PE(14:0/22:5)	760.49	737.50
P111	3.99_759.5764n	HPOS	pos	0.99188	24.48313	25.67087	26.31139	PC(22:1/12:0)	760.58	759.58
P112	2.83_761.4975n	HPOS	pos	3.43046	19.52151	21.11112	22.57936	PE(16:1/22:6)	762.50	761.50
P113	7.42_761.5948n	LPOS	pos	0.55228	24.41450	24.49721	25.08818	PC(16:0/18:0)	762.60	761.59
P114	3.87_766.5751m/z	HPOS	pos	1.95117	18.88569	19.57236	21.96914	Eicosapentaenoyl PAF C-16	766.58	765.57
P115	6.80_767.5835n	LPOS	pos	0.84191	23.09477	23.04873	24.28575	PC(O-16:1/18:0)	768.59	745.60
P116	6.73_771.5816n	LPOS	pos	1.02362	23.88949	23.74588	25.14503	PE(18:2/20:0)	772.59	771.58
P117	7.25_773.5978n	LPOS	pos	1.35995	24.23701	24.06872	25.74246	PE(16:1/22:0)	774.60	773.60
P118	7.96_773.6307n	LPOS	pos	1.65225	20.52136	20.04455	22.78901	PE(16:1/22:0)	774.64	773.63
P119	5.91_779.5524n	LPOS	pos	0.64321	26.33841	26.27589	27.04283	PC(20:5/16:0)	780.56	779.55
P12	4.92_184.0740m/z	HPOS	pos	1.97071	22.39217	23.27482	24.71036	Unannotated	184.07	NA
P120	3.92_782.5693m/z	HPOS	pos	2.80715	22.60970	24.17054	25.51375	PC(16:0/20:4)	782.57	764.43
P121	2.81_785.4975n	HPOS	pos	1.86046	21.10703	22.42626	23.35417	PE(18:3/22:6)	786.51	785.50
P122	7.10_785.5919n	LPOS	pos	0.58277	27.75586	27.66548	28.44215	PC(18:1/18:1)	786.60	785.59
P123	3.96_785.5934n	HPOS	pos	1.37441	23.70445	24.57715	26.01699	1,2-Dioleoyl PC	786.60	786.60
P124	3.87_750.5188n	HPOS	pos	1.55934	20.65590	21.55741	22.74503	PI(16:0/16:2)	789.48	806.49
P125	7.23_789.5311n	LPOS	pos	0.87163	23.57124	23.76451	24.34293	PC(15:0/20:4)	790.53	767.55
P126	6.72_796.5892m/z	LPOS	pos	1.20449	22.13017	22.22676	23.28062	PE(22:6/20:3)	796.59	813.53
P127	3.90_796.6005m/z	HPOS	pos	1.62046	19.37824	20.36207	21.85722	PC(O-18:1/O-18:1)	796.60	785.59
P128	7.33_800.6164m/z	LPOS	pos	1.87069	21.84736	21.52887	23.58930	PE(18:2/22:0)	800.62	799.61
P129	7.78_763.6281n	LPOS	pos	3.56481	20.36957	20.04268	23.52541	PE(16:1/24:0)	802.63	801.62
P13	6.09_184.0768m/z	LPOS	pos	0.45308	24.59390	24.55604	25.06763	Unannotated	184.08	NA
P130	6.20_805.5633n	LPOS	pos	1.15949	26.94618	27.00819	27.81247	PE(19:0/22:6)	806.57	805.56
P131	3.89_805.5623n	HPOS	pos	1.48991	23.62270	24.81904	25.68896	PE(18:4/22:6)	806.57	783.48
P132	5.99_805.5651n	LPOS	pos	0.38595	24.34711	24.37446	24.77033	PC(22:6/16:0)	806.57	805.57
P133	6.52_807.5826n	LPOS	pos	0.74158	25.22673	25.32281	25.98053	PC(22:5/16:0)	808.59	807.58
P134	6.66_807.5816n	LPOS	pos	0.57170	25.31586	25.35862	26.01070	PC(22:5/16:0)	808.59	807.58
P135	3.88_810.6001m/z	HPOS	pos	1.88479	22.92910	23.95799	25.71039	PC(18:0/20:4)	810.60	809.59
P136	7.06_809.5978n	LPOS	pos	0.73912	25.70329	25.77645	26.44891	PC(22:4/16:0)	810.61	809.60
P137	2.80_789.5293n	HPOS	pos	1.75514	21.57149	22.82417	23.93507	DG(18:0/20:4/0:0)	812.52	668.54

P138	7.28_812.6180m/z	LPOS	pos	0.76200	25.36413	25.40835	26.24927	PC(18:3/20:0)	812.62	811.61
P139	8.12_812.6791n	LPOS	pos	0.76885	24.34988	24.26683	24.99642	TG(15:0/16:1/16:0)	813.69	790.71
P14	2.84_205.1106n	HPOS	pos	1.18697	22.70555	23.65915	24.70160	Cerulenin	206.12	223.12
P140	7.62_813.6258n	LPOS	pos	1.17702	23.23803	23.18807	24.22093	PC(18:0/20:2)	814.63	813.63
P141	3.87_809.5934n	HPOS	pos	1.31268	23.18411	24.34597	25.24176	PC(18:1/22:6)	832.58	831.58
P142	6.87_833.5979n	LPOS	pos	1.06116	24.39619	24.53626	25.35121	PC(22:6/18:0)	834.61	833.60
P143	7.32_835.6101n	LPOS	pos	1.20812	22.60595	22.83313	23.77606	PC(22:5/18:0)	836.61	835.61
P144	10.80_830.7406n	LPOS	pos	0.86697	22.20689	22.14383	23.29711	TG(18:0/16:1/16:1)	853.73	830.74
P145	3.85_856.5817m/z	HPOS	pos	1.29529	21.30722	22.43016	23.25253	PC(20:3/22:6)	856.58	855.58
P146	3.85_871.5690n	HPOS	pos	1.13571	21.58823	22.59880	23.40151	Unannotated	872.56	NA
P147	11.09_842.7453n	LPOS	pos	0.61330	22.57659	22.64538	23.17992	Sulfatide (d18:1/22:0)	881.75	863.62
P15	6.03_221.0195m/z	HPOS	pos	1.27419	20.36211	21.17003	22.29912	3-Dechloroethylifosfamine	221.02	198.03
P16	5.82_241.1554m/z	HPOS	pos	1.82910	19.94669	21.01924	22.21459	Pirbuterol	241.16	240.15
P17	3.67_252.0263m/z	HPOS	pos	1.02226	20.38004	21.15703	22.21830	N-Acetyl-glutamyl-5-phosphate	252.03	269.03
P18	4.63_251.0707n	HPOS	pos	3.78912	18.12252	19.48801	20.87808	Unannotated	252.08	NA
P19	8.44_269.2289m/z	LPOS	pos	9.25898	14.19626	12.59262	22.17674	Vitamin A	269.23	286.23
P2	4.15_94.0664m/z	HPOS	pos	1.04395	20.02714	20.90415	21.83233	N-Acetyl-2,3-dihydro-1H-pyrrole	94.07	111.07
P20	6.02_257.1039n	HPOS	pos	2.87840	20.22650	21.75636	22.96917	Glycerophosphocholine	280.09	257.10
P21	2.57_287.2828n	HPOS	pos	12.29959	16.78090	21.08718	24.17214	Heptadecanoic acid	288.29	270.26
P22	1.24_288.2928m/z	LPOS	pos	11.51586	18.63279	22.10743	23.77030	Heptadecanoic acid	288.29	270.26
P23	2.51_316.3216m/z	HPOS	pos	12.59977	15.53254	19.40303	22.74747	Pristanic acid	316.32	298.29
P24	1.75_316.3239m/z	LPOS	pos	9.83148	18.38748	21.21541	22.97873	Nonadecanoic acid	316.32	298.29
P25	1.82_315.3154n	LPOS	pos	6.70582	19.41668	21.41817	22.96982	Nonadecanoic acid	316.32	298.29
P26	2.84_309.1419n	HPOS	pos	1.25552	20.55665	21.37027	22.54822	Gingerenone C	327.16	326.15
P27	1.85_317.0580n	HPOS	pos	1.94904	17.31314	18.13255	20.16662	Serotinose	335.10	312.11
P28	2.80_341.3058m/z	HPOS	pos	2.22854	19.11117	19.91147	21.76641	MG(18:0/0:0/0:0)	341.31	358.31
P29	2.84_358.2024m/z	HPOS	pos	1.27431	21.52715	22.40990	23.53460	Laudanosine	358.20	340.32
P3	6.02_103.1012n	HPOS	pos	1.80799	20.95249	21.89735	23.15252	Unannotated	104.11	NA
P30	2.45_357.3601n	HPOS	pos	5.90401	16.98303	17.26933	21.78147	Behenic acid	358.37	340.33
P31	2.71_358.3707m/z	LPOS	pos	6.90629	19.64799	19.79440	23.90891	Adrenoyl ethanolamide	358.37	375.31
P32	1.44_372.3136m/z	LPOS	pos	1.97011	20.13027	20.23779	21.92730	MG(0:0/18:2/0:0)	372.31	354.28
P33	1.60_398.3290m/z	LPOS	pos	2.76217	19.84870	19.87173	22.54527	MG(0:0/20:3/0:0)	398.33	380.29
P34	1.64_398.3291m/z	LPOS	pos	1.88393	20.20040	20.01461	22.35325	MG(0:0/20:3/0:0)	398.33	380.29
P35	1.97_400.3450m/z	LPOS	pos	1.22967	23.34747	23.33366	24.58163	L-Palmitoylcarnitine	400.34	400.35
P36	4.67_825.5862n	LPOS	pos	2.68475	18.80841	18.50910	21.32363	PC(18:0/20:4)	413.80	783.54
P37	2.13_426.3607m/z	LPOS	pos	1.82214	23.39994	23.45513	25.02475	Oleoylcarnitine	426.36	426.36
P38	5.23_429.3748m/z	LPOS	pos	1.98452	20.75701	20.86013	22.78952	13'-OH-a-Tocopherol	429.38	447.38
P39	0.79_440.1513n	LPOS	pos	7.19456	13.25161	12.91242	20.49870	Propylene glycol alginate	441.16	440.15
P4	4.92_104.1083m/z	HPOS	pos	1.21539	21.64241	22.58281	23.69344	Unannotated	104.11	NA
P40	4.93_436.2593n	HPOS	pos	1.31600	21.92736	23.00246	23.91075	LysoPA(0:0/18:1)	459.25	436.26
P41	1.66_463.2763n	HPOS	pos	3.28825	17.48160	18.78838	21.93949	Unannotated	464.28	NA
P42	3.28_437.3528n	HPOS	pos	11.67204	8.96800	8.51058	20.98107	Glycolithocholate acetate	476.34	458.30
P43	5.07_481.3521n	HPOS	pos	3.55225	15.19970	16.04836	19.07547	Unannotated	482.36	NA
P44	4.86_464.2894n	HPOS	pos	1.83632	19.61710	20.54475	21.91399	PA(O-20:4/2:0)	487.28	486.27

P45	2.73_487.2923n	HPOS	pos	6.85161	18.61474	18.88583	24.19034	Sodium glycocholate	488.30	487.29
P46	1.85_471.2965n	HPOS	pos	5.30398	17.37799	18.11258	22.48965	PS(17:0/0:0)	494.29	511.29
P47	1.75_471.2970n	HPOS	pos	4.82838	19.81625	21.29418	23.61801	PS(17:0/0:0)	494.29	511.29
P48	1.71_493.3193n	LPOS	pos	1.70048	20.28320	20.29466	21.67788	Homodolichosterone	494.33	476.35
P49	2.10_495.3363n	LPOS	pos	0.58077	25.38730	25.38667	25.96276	LysoPC(16:0/0:0)	496.34	495.34
P5	1.17_118.0290n	HPOS	pos	2.30602	21.02259	22.17585	23.90490	Adenine	136.06	135.05
P50	4.92_495.3318n	HPOS	pos	2.37218	22.07363	23.35927	24.54005	Unannotated	496.34	NA
P51	2.74_481.3197n	LPOS	pos	1.33229	21.11253	21.12611	21.99695	LysoPE(0:0/18:0)	504.31	481.32
P52	4.87_516.3060m/z	HPOS	pos	2.54640	18.64153	19.20131	21.87564	PE(19:1/0:0)	516.31	493.32
P53	4.93_518.3219m/z	HPOS	pos	1.20990	21.85903	22.91868	23.79401	LysoPC(16:0/0:0)	518.32	495.33
P54	4.92_519.3325n	HPOS	pos	1.54783	20.26207	21.09126	22.52551	Unannotated	520.34	NA
P55	1.78_519.3357n	LPOS	pos	1.00380	23.03626	22.98629	24.16100	LysoPC(18:2/0:0)	520.34	519.34
P56	1.87_519.3352n	LPOS	pos	1.06834	21.70685	21.46494	23.17028	LysoPC(18:2/0:0)	520.34	519.34
P57	10.50_521.4591m/z	LPOS	pos	1.93863	20.33299	20.09710	22.06859	DG(14:0/0:0/16:1)	521.46	538.46
P58	4.88_521.3474n	HPOS	pos	2.70254	20.09299	20.98878	23.33839	LysoPC(18:1)	522.36	521.35
P59	2.15_521.3507n	LPOS	pos	1.23473	22.82931	22.82759	24.16861	LysoPC(18:1/0:0)	522.36	521.35
P6	1.28_118.0290n	HPOS	pos	2.90816	21.51452	22.82741	24.66590	Adenine	136.06	135.05
P60	2.67_523.3667n	LPOS	pos	0.62351	24.94762	24.89435	25.63724	LysoPC(18:0)	524.37	523.37
P61	5.53_528.3899n	HPOS	pos	12.82061	9.46425	8.53695	23.60420	Panaxynol linoleate	529.40	506.41
P62	2.99_530.2752m/z	HPOS	pos	4.03237	19.42196	21.12928	22.64432	Glycochenodeoxychol ate-3-sulfate	530.28	529.27
P63	4.82_542.3222m/z	HPOS	pos	1.24795	21.24840	22.15155	23.22809	Unannotated	542.32	NA
P64	4.85_543.3317n	HPOS	pos	2.29735	21.19545	22.28005	23.68819	PC(20:4/0:0)	544.34	543.33
P65	1.79_543.3343n	LPOS	pos	1.14984	21.99367	22.14186	23.12500	LysoPC(20:4/0:0)	544.34	543.33
P66	4.85_523.3631n	HPOS	pos	1.81084	21.34121	22.50872	23.89037	PAF C-16	546.35	523.36
P67	10.80_549.4912m/z	LPOS	pos	1.19491	22.66458	22.54448	24.09952	DG(16:0/16:1/0:0)	549.49	566.49
P68	10.50_549.4900m/z	LPOS	pos	1.59009	21.59781	21.39191	23.26611	DG(16:0/16:1/0:0)	549.49	566.49
P69	2.37_537.2743n	HPOS	pos	7.44008	18.10525	19.92303	23.81962	Unannotated	560.26	NA
P7	4.15_136.0643n	HPOS	pos	1.04108	20.54710	21.45196	22.37193	2-Acetyl-3-methylpyrazine	137.07	136.06
P70	6.00_573.4907m/z	LPOS	pos	2.43962	20.56959	20.69277	22.88674	DG(16:0/0:0/18:3)	573.49	590.49
P71	2.90_575.5037m/z	HPOS	pos	1.82410	20.40204	21.47240	23.03094	DG(14:0/20:2/0:0)	575.50	592.51
P72	6.58_575.5072m/z	LPOS	pos	1.16840	24.35833	24.19689	25.64245	DG(16:1/0:0/18:1)	575.51	592.51
P73	11.09_577.5223m/z	LPOS	pos	0.61414	23.72852	23.82634	24.34476	DG(16:0/18:1)	577.52	594.52
P74	7.12_577.5227m/z	LPOS	pos	0.99567	22.74475	22.53870	24.29669	DG(16:0/0:0/18:1)	577.52	594.52
P75	2.07_286.0935n	HPOS	pos	6.00252	17.69084	19.10362	22.62242	Flavosativaside	595.18	594.16
P76	2.83_288.2676n	HPOS	pos	2.73801	19.68331	20.78996	22.29995	Unannotated	599.50	NA
P77	2.87_601.5189m/z	HPOS	pos	2.88568	19.94427	21.26342	23.22639	DG(14:0/22:3/0:0)	601.52	618.52
P78	6.70_601.5207m/z	LPOS	pos	1.53510	25.05966	24.79375	27.03091	DG(18:0/0:0/18:3)	601.52	618.52
P79	10.83_618.5257n	LPOS	pos	1.09992	23.34676	23.45896	24.32875	DG(16:0/20:3/0:0)	601.52	618.53
P8	1.55_136.0396n	HPOS	pos	0.48868	23.25833	24.63217	25.13480	Ascobate	159.03	176.03
P80	2.87_290.2602n	HPOS	pos	1.88798	20.85716	21.86045	23.25233	DG(14:0/22:2/0:0)	603.53	620.54
P81	11.11_603.5373m/z	LPOS	pos	0.89644	23.50940	23.64841	24.37468	DG(14:0/22:2/0:0)	603.54	620.54
P82	6.15_603.5381m/z	LPOS	pos	1.13684	22.97254	22.89858	24.22274	DG(18:1/0:0/18:1)	603.54	620.54
P83	2.81_622.4972n	HPOS	pos	2.02358	21.64512	22.76994	24.17466	Unannotated	623.50	NA
P84	6.35_623.5070m/z	LPOS	pos	1.07780	24.80978	24.93910	25.73105	CL(1'-[20:0/20:0],3'-[18:2/16:0])	623.51	1489.10
P85	2.80_627.5354m/z	HPOS	pos	2.00743	21.55799	22.35423	24.11903	DG(20:3/18:1/0:0)	627.54	644.54

P86	7.23_627.5343m/z	LPOS	pos	0.69757	26.37040	26.45836	27.04865	DG(20:3/18:1/0:0)	627.54	644.54
P87	6.14_644.5413n	LPOS	pos	0.61751	24.40685	24.52429	25.15900	DG(20:3/18:1/0:0)	627.54	644.54
P88	7.42_314.2625n	LPOS	pos	1.53939	23.27584	23.31368	24.52226	DG(16:1/22:2/0:0)	629.55	646.55
P89	8.77_629.6122n	LPOS	pos	0.61392	23.32947	23.29445	23.76922	Unannotated	630.62	NA
P9	3.13_160.0998n	HPOS	pos	4.44704	17.78733	18.75939	21.54508	3-Methylthio-heptanal	178.13	160.09
P90	8.84_647.4592m/z	LPOS	pos	5.00331	19.69777	19.13391	23.23794	GOTP	647.46	646.46
P91	2.78_650.5276n	HPOS	pos	2.02965	20.56342	21.58181	23.31033	DG(18:0/20:4/0:0)	651.53	668.54
P92	7.03_651.5369m/z	LPOS	pos	1.99696	24.06993	24.07281	25.84153	DG(20:5/20:1/0:0)	651.54	668.54
P93	5.98_651.5376m/z	LPOS	pos	1.24038	23.76652	23.90417	24.78946	Unannotated	651.54	NA
P94	4.38_649.4313n	HPOS	pos	2.06730	20.50137	21.82311	23.10656	Unannotated	672.42	NA
P95	3.33_698.2762n	HPOS	pos	7.35909	15.44431	17.14062	21.27642	Unannotated	699.28	NA
P96	0.59_676.2976n	LPOS	pos	9.59619	12.62312	13.88014	21.01726	Mulberrofuran T	699.29	716.30
P97	5.54_703.5176n	LPOS	pos	2.29809	22.57263	22.36144	25.09801	PC(16:1/14:0)	704.53	703.52
P98	6.07_705.5352n	LPOS	pos	0.94528	23.56598	23.63789	24.76184	PE(12:0/21:0)	706.54	705.54
P99	5.89_717.5338n	LPOS	pos	2.32827	21.65352	21.67124	23.49187	PE(16:0/18:1)	718.54	717.53

Appendix Table S4. List of manually-annotated putative metabolites and their information

Peak ID	Mass features	m/z	Retention time (min)	Peak width (min)	MS/MS identified	Assignment	Adduct	Molecular Formula	Theoret-ical m/z	Mass error (ppm)	LoA *	Database ID	Description	ME-region	Known relation to ME-regions, HCC or liver diseases	References (DOI)
N14	2.67_465.3048m/z	465.3040	2.667	0.64	2.55_465.3040	Cholesterol sulfate	M-H	C27H46O4S	465.3039	0.75	2	HMDB0000653	Endogenous steroids that involves in steroid synthesis, influences lipid metabolism, stabilizes cell membranes, regulates the activity of functional proteins, etc	ME-Fibrotic	Higher levels of plasma cholesterol sulfate in patients with liver cirrhosis; Tumor cells that produce cholesterol sulfate exhibit resistance to cancer-specific T-cell transfer and blocking immune checkpoint	10.1194/jlr.R300005-JLR200 10.1007/BF02536238 10.1093/intimm/dxac002
N71	5.93_874.5575n	873.55	5.928	0.24	5.74_873.5498	PI(17:0/20:3)	M-H	C46H83O13P	873.5493	0.41	3	LMGP06010209	Glycerophosphoinositols that contain heptadecenoic acid and eicosadienoic acid side chains. They are bioproducts of phospholipase A2 and lysolipase activities that involves in regulation of cell proliferation, actin cytoskeleton organisation and dependent processes, etc	ME-High grade		10.1007/s00018-009-0113-4
P29	2.84_358.2024m/z	358.203	2.838	0.10	2.88_358.2031	Laudanosine	M+H	C21H27NO4	358.2013	3.23	3	HMDB0030213	Principal metabolites of muscle relaxant atracurium used in anesthesia	ME-Normal	In hepatic failure, its elimination halflife is prolonged but only moderate accumulation occurs in adults	10.1017/s0265021502000777
P37	2.13_426.3607m/z	426.358	2.134	0.16	1.94_426.3584	Oleoylcarnitine	M+H	C25H47NO4	426.3583	6.92	3	HMDB0005065	Oleic acid esters of carnitine that involve in beta-oxidation where fatty/organic acids are transported from the cytoplasm into the mitochondria to be broken down to produce energy	ME-Necrotic	Accumulates through suppression of fatty acid β-oxidation, can enhance hepatocarcinogenesis via STAT3 activation	10.1136/gutjnl-2017-315193
P38	5.23_429.3748m/z	429.369	5.231	0.60	5.12_429.3694	13'-OH-α-Tocopherol	M+H-H_2O	C29H50O3	447.3838	4.78	2	HMDB0012559	Intermediates in the α-Tocopherol (Vitamin E) metabolism pathway	ME-Necrotic		

P42	3.28_437.3528n	476.334	3.275	0.17	3.30_476.3342	Glycolithocholate acetate	M+H	C28H45O5N	476.3370	2.29	2		Conjugate base of glycolithocholic acids which are glycine-conjugated form of the secondary bile acid lithocholic acid	ME-Normal	Glycolithocholic acids showed a potential ability to differentiate metabolic dysfunction-associated steatohepatitis (MASH)	10.1128/msystems.00805-21<br>10.1152/physrev.00010.2008<br>10.3390/biom13091356
P58	4.88_521.3474n	522.3551	4.877	0.30	4.93_522.3551	LysoPC(18:1)	M+H	C26H52NO7P	522.3560	-1.48	3	HMDB10385	Lysophosphatidylcholines (LPC) that contain vaccenic acid side chain and are derived by the cleaving of phosphatidylcholine via the action of phospholipase A2 and/or by the transfer of fatty acids to free cholesterol via lecithin-cholesterol acyl transferase. LPCs activate multiple signaling pathways that are involved in oxidative stress and inflammatory responses	ME-Normal	In liver, LPCs upregulate genes involved in cholesterol biosynthesis and downregulate genes involved in hepatic fatty acid oxidation; Downregulated in HCC plasma relative to healthy volunteer plasma	10.1158/0008-5472.CAN-11-0885<br>10.3390/ijms20051149
P60	2.67_523.3667n	524.3730	2.667	0.41	2.43_524.3730	LysoPC(18:0)	M+H	C26H54NO7P	524.3716	5.53	3	HMDB0010384	Lysophosphatidylcholines (LPC) that contain stearic acid side chain and are derived by the cleaving of phosphatidylcholine via the action of phospholipase A2 and/or by the transfer of fatty acids to free cholesterol via lecithin-cholesterol acyl transferase. LPCs activate multiple signaling pathways that are involved in oxidative stress and inflammatory responses	ME-Low grade	In liver, LPCs upregulate genes involved in cholesterol biosynthesis and downregulate genes involved in hepatic fatty acid oxidation; Downregulated in HCC plasma relative to healthy volunteer plasma	10.1158/0008-5472.CAN-11-0885<br>10.3390/ijms20051149
P73	11.09_577.5223m/z	577.516	11.086	0.14	10.92_577.5156	DG(16:0/18:1)	M+H-H_2O	C37H70O5	595.5302	5.42	3	HMDB0007101	Diacylglycerols (DAG) which contain palmitic acid and oleic acid and are precursors to triacylglycerols (triglyceride). DAGs function as a second messenger signaling lipid	ME-Steatotic	Increased DAGs characterize hepatic lipid changes in progression of human non-alcoholic fatty liver disease	10.1371/journal.pone.0022775

P120	3.92_782.5693m/z	782.571	3.915	0.15	4.00_782.5707	PC(16:0/20:4)	M+H	C44H80NO8P	782.5700	-0.23	2	HMDB0007983	Phosphatidylcholines (PC) that contain eicsoatetraenoic acid and palmitic acid side chains and are major component of biological membranes	ME-Transformed/ Low grade	Metabolic alterations of PLs play important roles in pathogenesis and progression of HCC	10.1007/s12072-020-10056-8
P135	3.88_810.6001m/z	810.601	3.879	0.25	3.99_810.6011	PC(18:0/20:4)	M+H	C46H84NO8P	875.5650	-0.82	2	HMDB0008049	Phosphatidylcholines (PC) that contain stearic acid and arachidonic acid and are major component of biological membranes	ME-Transformed	Metabolic alterations of PLs play important roles in pathogenesis and progression of HCC	10.1007/s12072-020-10056-8

***LoA: Level of Assignment:**
1: Accurate mass matched to database - Tentative assignment;
2: Accurate mass matched to database and tandem MS spectrum matched to in silico fragmentation pattern;
3: Tandem MS spectrum matched to database or literature;
4: RT matched to standard compound; and
5: MS/MS spectrum matched to standard compound.

Abbreviations:
m/z: Mass charge ratio;
PC: Phosphatidylcholine;
PI: Phosphatidylinositol;
DG: Diacylglycerol
