# Supplementary material for: Spatially-guided metabolomics profiling of metabolic regions in human tumor tissues
Source: Mol Syst Biol. 2026 Apr 1;22(7):1132–60. doi: 10.1038/s44320-026-00205-w (PMC13328631; doi:10.1038/s44320-026-00205-w)
Supplement: Supplementary file 9 — Expanded View Figures [file 44320_2026_205_MOESM9_ESM.pdf]

Expanded View Figures

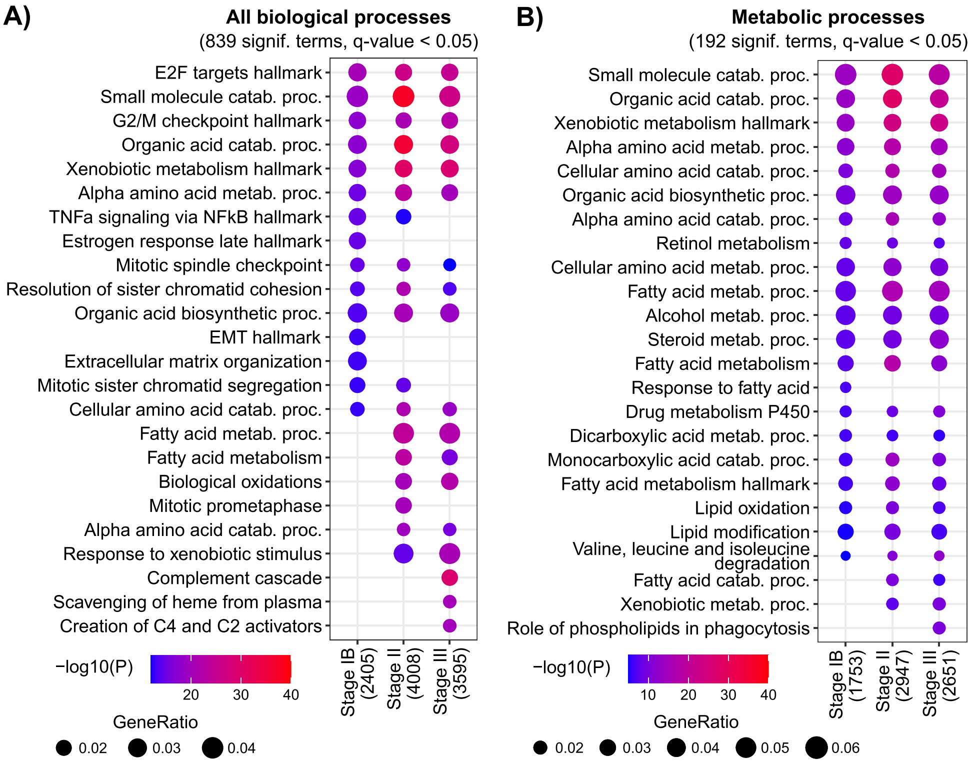

Figure EV1. Biological and metabolic processes that changed at different HCC stages.

Dot plots showing the top significantly enriched or de-enriched (**A**) biological processes or (**B**) metabolic processes in the differentially expressed genes between adjacent-normal tissues and Stage IB, II, or III tumor sections (two-sided hypergeometric tests; q value = expected positive false discovery rate obtained from the Storey's procedure (Storey, 2002); numbers after the x axis labels = the numbers of found differentially expressed genes).

© The Author(s)

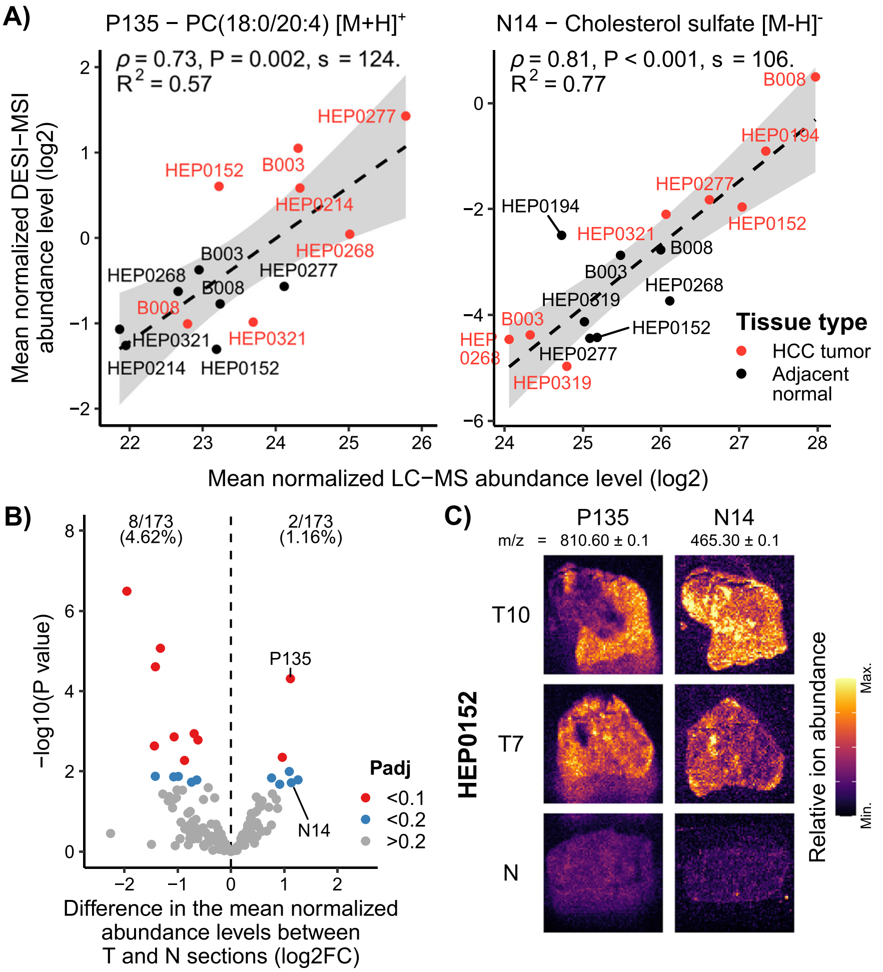

Figure EV2. Highly abundant putative metabolites that changed significantly between matched tumor and adjacent-normal tissue sections.

(**A**) Scatter plots showing the correlations between mean normalized DESI-MSI versus LC-MS abundance levels for PC(18:0/20:4) (left) or cholesterol sulfate (right) (ρ = Spearman's rank correlation coefficients, P = P values of the Spearman's rank correlation coefficient values). (**B**) Volcano plot showing highly abundant putative metabolites with significantly changed averaged tissue abundance levels between the adjacent normal tissue and tumor sections (two-sided t test, Padj = BH-adjusted P values). (**C**) Exemplary DESI-MSI images of PC(18:0/20:4) (left) and cholesterol sulfate (right) ions on three selected tissue sections from patient HEP0152 (N = adjacent normal tissue sections, T7 and T10 = HCC tumor sections).

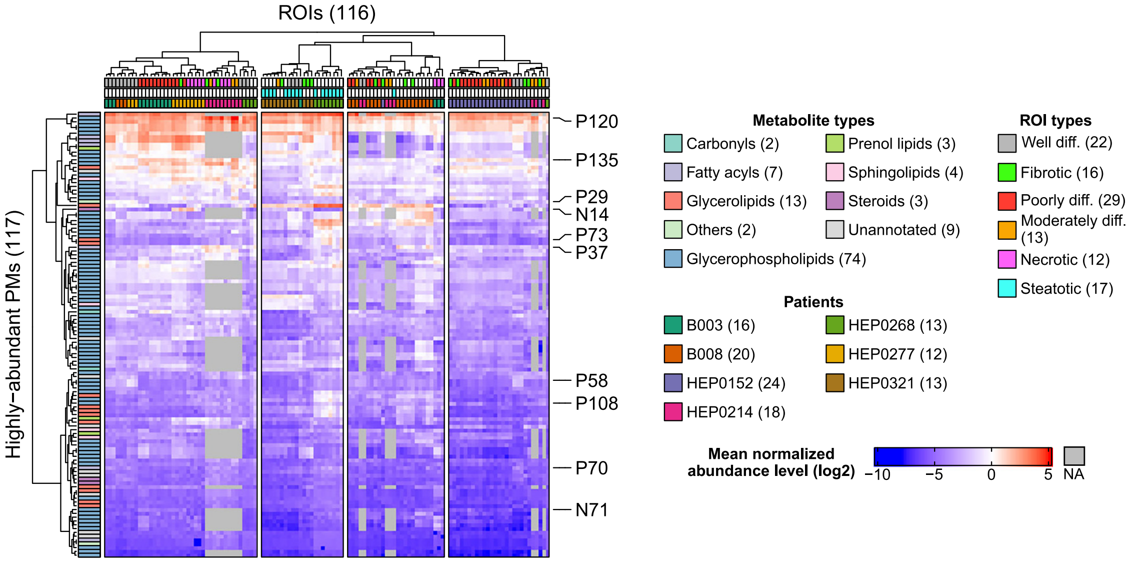

Figure EV3. The abundance levels of highly abundant putative metabolites on DESI-MSI ROIs.

Heatmap showing the mean normalized abundance levels of the 117 highly abundant putative metabolites that could be detected in more than 50% of these ROIs on the DESI-MSI images. The rows and columns were clustered using hierarchical clustering and Ward's linkage. Selected putative metabolites of interest were highlighted [P29 = laudanosine, P37 = oleoylcarnitine, P58 = lysoPC(18:1), P70 = DG(16:0/18:3), P73 = DG(16:0/18:1), P108 = PC(15:0/18:0), P120 = PC(16:0/20:4), P135 = PC(18:0/20:4), N14 = cholesterol sulfate, and N71 = PI(17:0/20:3)] (NA = data not available because negative DESI-MSI ions were not collected for HEP0214).

© The Author(s)

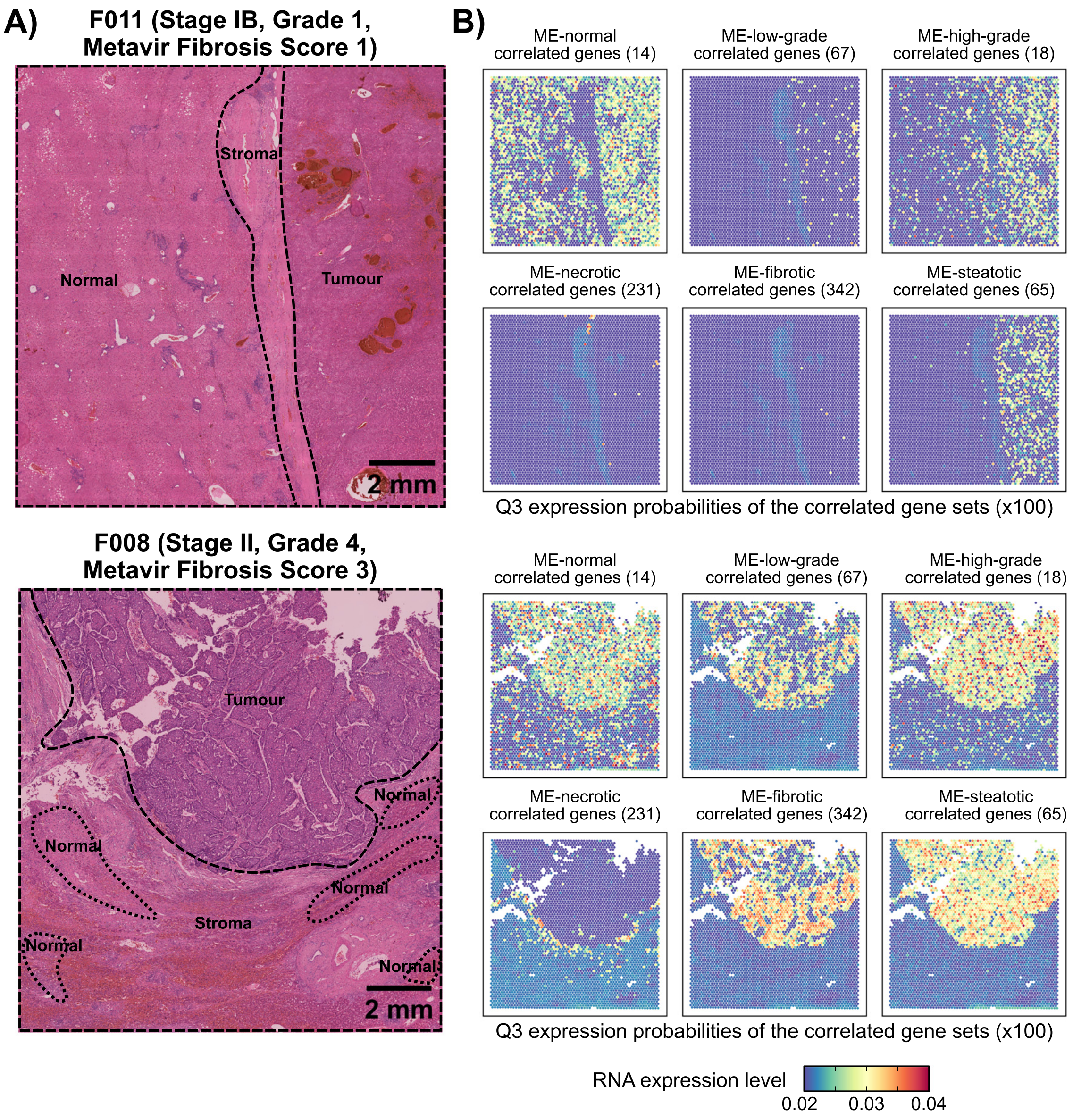

Figure EV4. Spatial RNA expression patterns of gene sets significantly correlated with discriminative metabolites from each MER.

(**A**) H&E images showing the tissue areas captured by the spatial transcriptomics slides for HCC tumors from patients F008 and F011. (**B**) Heatmaps showing the 75th percentiles (Q3) of the expression probabilities of all the positively correlated gene sets (numbers after the correlated gene-set labels = the numbers of genes from the correlated gene sets also detected by the spatial transcriptomics assays).
