# Supplementary material for: Spatially-guided metabolomics profiling of metabolic regions in human tumor tissues
Source: Mol Syst Biol. 2026 Apr 1;22(7):1132–60. doi: 10.1038/s44320-026-00205-w (PMC13328631; doi:10.1038/s44320-026-00205-w)
Supplement: Supplementary file 1 — Peer Review File [file 44320_2026_205_MOESM1_ESM.pdf]

molecular systems biology

Spatially-guided metabolomics profiling of metabolic regions in human tumor tissues

Jia-Ying Lee, Jingtao Zhang, Sin-Chi Chew, Shihleone Loong, Liang Xu, Jia-Wen Kong, Fedor Grigoryev, Alexander CHUNG, Jin-Yao Teo, Peng CHEOW, Glenn Bonney, Brian GOH, Wei-Qiang Leow, Yulan Wang, Lit-Hsin Loo, and Pierce CHOW

Corresponding author(s): Lit-Hsin Loo (loolh@bii.a-star.edu.sg) , Pierce CHOW (pierce.chow@duke-nus.edu.sg), Yulan Wang (yulan.wang@ntu.edu.sg)

Review Timeline:

Submission Date:	15th Jul 25
Editorial Decision:	15th Sep 25
Revision Received:	4th Dec 25
Editorial Decision:	5th Feb 26
Revision Received:	7th Mar 26
Accepted:	12th Mar 26

Editor: Jingyi Hou

Transaction Report:

(Note: With the exception of the correction of typographical or spelling errors that could be a source of ambiguity, letters and reports are not edited. Depending on transfer agreements, referee reports obtained elsewhere may or may not be included in this compilation. Referee reports are anonymous unless the Referee chooses to sign their reports.)

15th Sep 2025

Manuscript Number: MSB-2025-13233
Title: Spatially-guided metabolomics profiling of heterogeneous human tumor tissues
Author: Jia-Ying Lee
Jingtao Zhang
Sin-Chi Chew
Shihleone Loong
Liang Xu
Jia-Wen Kong
Fedor Grigoryev
Alexander CHUNG
Jin-Yao Teo
Peng CHEOW
Glenn Bonney
Brian GOH
Wei-Qiang Leow
Yulan Wang
Lit-Hsin Loo
Pierce CHOW

Dear Dr Loo,

Thank you for submitting your work to Molecular Systems Biology. First of all, I would like to apologise for the slow process. We have now heard back from two of the three reviewers who agreed to evaluate your manuscript. Unfortunately, after a series of reminders we did not manage to obtain a report from Reviewer #1. In the interest of time, and since the recommendations of the other two reviewers are quite similar, I prefer to make a decision now rather than further delaying the process. If we receive the comments from Reviewer #1, we will send them to you, and you can address the issues raised by Reviewer #1 together with those raised by the other two reviewers. You will see from the comments below that Reviewers #2 and #3 find the manuscript to be of potential interest. They raise, however, several important points, which should be convincingly addressed in a revision of this work.

I think that the recommendations of the reviewers are rather clear so there is no need to repeat the points listed below. In particular, both reviewers had concerns regarding the novel biological insights and the overall clinical relevance provided, which need to be strengthened and better demonstrated. All other issues raised by the reviewers need to be satisfactorily addressed as well. As you may already know, our editorial policy allows in principle a single round of major revision so it is essential to provide responses to the reviewers' comments that are as complete as possible. Please feel free to contact me in case you would like to discuss in further detail any of the issues raised by the reviewers.

On a more editorial level, we would ask you to address the following issues:

- Please provide a .docx formatted version of the manuscript text (including legends for main figures, EV figures and tables). Please make sure that the changes are highlighted to be clearly visible.

- Please provide individual production quality figure files as .eps, .tif, .jpg (one file per figure).

-Please provide a .docx formatted letter INCLUDING the reviewers' reports and your detailed point-by-point responses to their comments. As part of the EMBO Press transparent editorial process, the point-by-point response is part of the Review Process File (RPF), which will be published alongside your paper.

-Please note that all corresponding authors are required to supply an ORCID ID for their name upon submission of a revised manuscript.

-We replaced Supplementary Information with Expanded View (EV) Figures and Tables that are collapsible/expandable online (see examples in http://msb.embopress.org/content/11/6/812). A maximum of 5 EV Figures can be typeset. EV Figures should be cited as 'Figure EV1, Figure EV2" etc... in the text and their respective legends should be included in the main text after the legends of regular figures.

Additional Tables/Datasets should be labeled and referred to as Table EV1, Dataset EV1, etc. Legends have to be provided in a separate tab in case of .xls files. Alternatively, the legend can be supplied as a separate text file (README) and zipped together with the Table/Dataset file.

For the figures and tables that you do NOT wish to display as Expanded View figures, they should be bundled together with their legends in a single PDF file called *Appendix*, which should start with a short Table of Content. Each legend should be below the corresponding Figure/Table in the Appendix. Appendix figures and tables should be referred to in the main text as: "Appendix Figure S1, Appendix Figure S2, Appendix Table S1" etc. See detailed instructions regarding expanded view here: https://www.embopress.org/page/journal/17444292/authorguide#expandedview.

-Before submitting your revision, primary datasets (and computer code, where appropriate) produced in this study need to be deposited in an appropriate public database (seehttp://msb.embopress.org/authorguide - dataavailability https://www.embopress.org/page/journal/17444292/authorguide#dataavailability).

Please remember to provide a reviewer password if the datasets are not yet public.
The accession numbers and database should be listed in a formal "Data Availability" section (placed after Materials & Method) that follows the model below (see also https://www.embopress.org/page/journal/17444292/authorguide#dataavailability). Please note that the Data Availability Section is restricted to new primary data that are part of this study.
Data availability
The datasets (and computer code) produced in this study are available in the following databases:
- RNA-Seq data: Gene Expression Omnibus GSE46843 (https://www.ncbi.nlm.nih.gov/geo/query/acc.cgi?acc=GSE46843)
- [data type]: [name of the resource] [accession number/identifier/doi] ([URL or identifiers.org/DATABASE:ACCESSION])
*** Note - All links should resolve to a page where the data can be accessed. ***

- At EMBO Press we ask authors to provide source data for the main manuscript figures. You will receive a separate email with instructions for providing source data with your revised manuscript, including how to upload and organize the files.
Additional information on source data and instruction on how to label the files are available

- Our journal encourages inclusion of *data citations in the reference list* to directly cite datasets that were re-used and obtained from public databases. Data citations in the article text are distinct from normal bibliographical citations and should directly link to the database records from which the data can be accessed. In the main text, data citations are formatted as follows: "Data ref: Smith et al, 2001". In the Reference list, data citations must be labeled with "[DATASET]". A data reference must provide the database name, accession number/identifiers and a resolvable link to the landing page from which the data can be accessed at the end of the reference. Further instructions are available at .

- We updated our journal's competing interests policy in January 2022 and request authors to consider both actual and perceived competing interests. Please review the policy https://www.embopress.org/competing-interests and update your competing interests if necessary.
Please use the heading "Disclosure statement and competing interests".

- All Materials and Methods need to be described in the main text using our 'Structured Methods' format. According to this format, the Methods section includes a Reagents and Tools Table (listing key reagents, experimental models, software and relevant equipment and including their sources and relevant identifiers) followed by a Methods and Protocols section describing the methods, ideally using a step-by-step protocol format. The aim is to facilitate adoption of the methodologies across labs.

Please download and fill our Reagents and Tools Table template (.docx), which you can find in our author guidelines: https://www.embopress.org/page/journal/17444292/authorguide#structuredmethods.

When submitting your revised manuscript, please do not include the Reagents and Tools Table in the Methods section of the manuscript but upload it as a separate file choosing the file type "Reagent Table".

An example of a Method paper with Structured Methods can be found here:
https://www.embopress.org/doi/10.15252/msb.20178071.

-Regarding data quantification:
Please ensure to specify the name of the statistical test used to generate error bars and P values, the number (n) of independent experiments (please specify technical or biological replicates) underlying each data point and the test used to calculate p-values in each figure legend. Discussion of statistical methodology can be reported in the materials and methods section, but figure legends should contain a basic description of n, P and the test applied.
Graphs must include a description of the bars and the error bars (s.d., s.e.m.).
Please also include scale bars in all microscopy images.

- Please provide a "standfirst text" summarizing the study in one or two sentences (approximately 250 characters, including space), three to four "bullet points" highlighting the main findings and a "synopsis image" (550px width and 400-600 px height, PNG format) to highlight the paper on our homepage.
Here are a couple of examples:
https://www.embopress.org/doi/10.15252/msb.20199356

https://www.embopress.org/doi/10.15252/msb.20209475
https://www.embopress.org/doi/10.15252/msb.209495

When you resubmit your manuscript, please download our CHECKLIST (https://www.embopress.org/pb-assets/embo-site/EMBO%20Press%20Author%20Checklist-1642513524327.xlsx) and include the completed form in your submission. *Please note* that the Author Checklist will be published alongside the paper as part of the transparent process (https://www.embopress.org/page/journal/17444292/authorguide#transparentprocess).

If you feel you can satisfactorily deal with these points and those listed by the referees, you may wish to submit a revised version of your manuscript. Please attach a covering letter giving details of the way in which you have handled each of the points raised by the referees. A revised manuscript will be once again subject to review and you probably understand that we can give you no guarantee at this stage that the eventual outcome will be favorable.

I look forward to receiving the revised manuscript soon.

Kind regards,
Jingyi

Jingyi Hou, PhD
Senior Editor
Molecular Systems Biology

We realize that it is difficult to revise to a specific deadline. In the interest of protecting the conceptual advance provided by the work, we recommend a revision within 3 months (14th Dec 2025). Please discuss the revision progress ahead of this time with the editor if you require more time to complete the revisions. Use the link below to submit your revision:

*** PLEASE NOTE *** As part of the EMBO Press transparent editorial process initiative (see our Editorial at https://dx.doi.org/10.1038/msb.2010.72), Molecular Systems Biology publishes online a Review Process File with each accepted manuscripts. This file will be published in conjunction with your paper and will include the anonymous referee reports, your point-by-point response and all pertinent correspondence relating to the manuscript. If you do NOT want this File to be published, please inform the editorial office at contact@molsystbiol.org within 14 days upon receipt of the present letter.

Reviewer #2:

The submitted manuscript presents a multi-omics analysis of the tumor microenvironment in primary human liver cancer focusing on metabolomic and transcriptomic alterations across clinical stages of hepatocellular carcinoma (HCC). By using a combination of bulk and spatial metabolomics as well as bulk and spatial transcriptomics, the authors characterize metabolic and transcriptional patterns associated with tumor progression and other pathological processes such as steatosis, fibrosis and necrosis. Metabolic features associated with different histopathological alterations are identified and used to create spatially segmented metabolite maps and to build regression models for estimating tumor composition from bulk LC-MS/MS data. The presented workflow may encourage future investigations of tumor heterogeneity and metabolic biomarker discovery in other cancer types.

This work contributes to the growing body of research on tumor heterogeneity by profiling metabolic and transcriptomic changes in HCC. In its current state, the manuscript displays and discusses a huge amount of data and reads in larger parts convoluted and complex making it hard for the reader to focus on the scientific findings of the paper.
The study is largely descriptive and does not identify novel mechanistic insights into HCC tumorigenesis which limits its impact. The developed workflow and bioinformatics pipeline is well documented and transparent which can serve as an example for future studies on cancer metabolism and tissue heterogeneity. The manuscript is of interest to groups focused on integrating omics data to understand tumor microenvironments.

Major points:

• The manuscript needs major revisions in view of presentation and argumentation both for the figures and text. In its current state, it is very hard for the reader to focus on scientific findings or the concept of the developed workflow because both figures and text are overloaded with details that do not contribute to the story line of the paper.
• Figure presentation:
To enhance clarity and maintain focus on the main message, it would be helpful to streamline the figures by moving secondary information to the Supplementary. For example in Figure 1, we suggest enlarging panel (a) to emphasize the workflow while relocating panels (b) and (c) to the Supplementary. The icon representing the liver tumor currently in (b) could be incorporated directly into (a) as the starting point for the LC-MS/MS analysis. Similarly, we recommend placing greater emphasis on panels (d) and (f) while moving (e) and (g) to the Supplementary.
Figure 2 would also profit from reducing the number of individual data elements and focusing on the main findings from the metabolomics side. The images of the H&E are hardly visible in g), maybe one section could be presented in more detailed instead of presenting six sections the same time. Information in h) is somewhat repetitive to 1c). Elements i), j) and k) do not add any elemental information and can be summarized briefly in the text or moved to the Supplementary. The other figures, especially Figure 6, would also benefit from a more focused presentation by reducing the number of individual data elements.

The manuscript is at present largely organized by figure. It would be essential to the success of the manuscript to improve the story line of the manuscript by reducing the amount of descriptive details and instead discuss all main elements of the figures in detail (after revising the figures and distilling to main findings).
Right now, the text is descriptive and does not succeed in coming to broader scientific conclusions. For example, in the paragraph titled "LC-MS and DESI-MSI abundance levels of highly abundant metabolites are positively correlated" each sentence refers to a single panel from Figure 2 creating a somewhat repetitive and list-like structure. We recommend to discuss main findings in more detail and move background information that does not add to the key understanding of clinical findings or the established workflow to the Supplementary.
• Nomenclature of metabolites:
The manuscript would benefit from using a consistent and standardized nomenclature for metabolites. For instance, in Figure 2 panel (b) uses metabolite names, panels (m) and (n) use both names and internal abbreviations while panel (p) uses abbreviations only. It would be beneficial to refer to the metabolites in a uniform fashion ideally following established conventions e.g. LipidMaps. Currently, glycerophospholipids are not named coherently throughout the manuscript. Sometimes they are referred to by abbreviation (PC(20:4/16:0)) and sometimes with full name (1-stearoyl-2-arachidonoyl-sn-glycero-3-phosphocholine, SAPC) for example in Figure 4 and throughout the text. Similar considerations apply to other lipid annotations throughout the manuscript. Publications on best practices in MS-based lipidomics may provide further guidance on this point.
We understood the manuscript that only 11 metabolites in total are annotated manually and in full ID, this is a very low number from 3 different runs per sample. How is an interpretation possible together with transcriptomics data if the coverage of metabolism is that different?

• Meaningfulness of the developed workflow:
The presented workflow enables to predict composition of bulk samples after training regression models on spatial metabolomics and histological data. We acknowledge this as an achievement of the authors but question the clinical relevance for diagnostics in a clinical setting. The manuscript offer no insights in how this may benefit HCC diagnostics / research. Its not clear at the moment how a clinical application is implemented and is limiting the potential of this study to inspire other researchers.

Minor Points:
• Metabolomics: Little information is provided on the exact parameters of LC-MS/MS analysis. More details and acquisitions settings should be included in a revised form of the manuscript.
• Abbreviations: Many abbreviations are used throughout the manuscript (both text and figures) and limit the flow of the text. Consider reducing the number of abbreviations both for the established workflow and identified metabolites/biomarkers.
• Technical details: We appreciate the thorough documentation of the workflow and analyses but they somewhat interrupt the flow of the text. For example in paragraph 2 ("Different liver cancer..."), the exact mass range for LC-MS/MS analysis and the number of individual sections analyzed may be presented in Methods/Supplementary. Similar recommendations apply to other parts of the text.
• P3, L11-14 sentence L12 makes little sense
L14 irreproducibility is like a technical bias - but the papers state different outcomes of studies
L18 intact tissue- biopsy?

• P4 - L1 identification of lipids with MS1 only is anyways not correct based on standard reporting procedures, cite also those and the bottleneck of MS2 missing in large extend from MSI

• No LCMS data in data repository found after locking in - this needs to change

Reviewer #3:

This manuscript presents Spatially-guided MEtabolomics (SgME) profiling, an integrative framework combining LC-MS/MS, DESI-MSI, histopathology, and RNA-seq to delineate intra-tumoral heterogeneity (IntraTH) in primary liver cancers. The authors apply their approach to 117 samples from 26 patients, generating "metabolic region (MER)" maps and training regression models to deconvolve bulk metabolomics into spatial components.

The topic is timely and important: the reconciliation of bulk metabolomics with spatially resolved MSI is a major methodological gap in cancer systems biology. The scale of the dataset (multi-region, multi-modal) is commendable, as is the effort to link metabolites with histological features and transcriptomic pathways.

However, while the approach is innovative, several conceptual, methodological, and validation issues limit the current impact. Clarification and strengthening are required before this work can be considered for publication in Molecular Systems Biology.

Major Comments

1. Markers of metabolic regions (MERs)

Markers of metabolic regions represent a key information allowing the validate the data, the data analysis pipeline, and the results. Some additional evidence would be appreciated to address the following concerns.

First, some of the markers are not common lipids and include fatty acyl chains with odd carbons: PC(19:0/11:0), PI(17:1/20:2), TG(15:0/16:1/16:0) etc. Such lipids are not common in mammalian cells and additional evidence is needed to validate the correctness of their structural identities. The presented MS/MS spectrum for PI(17:1/20:2) in FIg S6 is not convincing as it shows C19:2 as the highest fragment. MS/MS spectra for other uncommon lipids are not shown.

Second, there is a number of non-endogenous metabolites identified, including laudonosine, tocopherol, and tocotrienol. Please demonstrate LC-MS/MS results for their identities. The presented MS/MS spectra in Fig S6 for laudanosine and tocopherol are not convincing as their fragments are not annotated and there are no spectra of authentic standards.

Moreover, the authors should explicitly state MSI annotation confidence levels and discuss risks of mis-annotation.

2. Definition and novelty of MERs

The proposed "metabolic regions (MERs)" are defined by integrating LC-MS-identified metabolites with MSI and histopathology.

It remains unclear whether MERs represent new biological entities or simply reflect re-labeled histopathological categories. The claim that MERs cannot be identified by clustering alone should be better substantiated.

3. Integration with transcriptomics (COMET's Path analysis)

The correlation-based integration of metabolite abundance with bulk RNA-seq is interesting, but risks spurious associations given the small sample size per modality and high dimensionality.

No multiple-testing correction across metabolite-gene correlations is described.

Padj < 0.1 seems permissive and goes against the state of the art. The authors should present the results with more statistically stringent selection (pvalue<0.05, multiple-testing correction) and discuss the differences.

Spatial transcriptomics validation is limited to two tumors only. The authors should discuss the limitations and biases of this approach.

4. Machine learning models and generalization

Regression models predicting MER composition from bulk LC-MS are promising, but performance metrics (R^2 up to 0.93) seem unrealistically high. Overfitting risk should be explicitly addressed.

5. Biological insights and clinical relevance

The manuscript identifies clusters of metabolites (e.g., low-grade+/necrotic-) that would be invisible in bulk profiling. This is a strong result. However, their biological significance (drivers of tumor progression, therapeutic vulnerability) is not fully developed.

6. Positioning relative to existing literature

The authors should more clearly contrast SgME with other integrative frameworks and methods. The degree of conceptual novelty is somewhat overstated.

Specific Responses to Reviewer #2's Comments:

The submitted manuscript presents a multi-omics analysis of the tumor microenvironment in primary human liver cancer focusing on metabolomic and transcriptomic alterations across clinical stages of hepatocellular carcinoma (HCC). By using a combination of bulk and spatial metabolomics as well as bulk and spatial transcriptomics, the authors characterize metabolic and transcriptional patterns associated with tumor progression and other pathological processes such as steatosis, fibrosis and necrosis. Metabolic features associated with different histopathological alterations are identified and used to create spatially segmented metabolite maps and to build regression models for estimating tumor composition from bulk LC-MS/MS data. The presented workflow may encourage future investigations of tumor heterogeneity and metabolic biomarker discovery in other cancer types.

This work contributes to the growing body of research on tumor heterogeneity by profiling metabolic and transcriptomic changes in HCC. In its current state, the manuscript displays and discusses a huge amount of data and reads in larger parts convoluted and complex making it hard for the reader to focus on the scientific findings of the paper.

The study is largely descriptive and does not identify novel mechanistic insights into HCC tumorigenesis which limits its impact. The developed workflow and bioinformatics pipeline is well documented and transparent which can serve as an example for future studies on cancer metabolism and tissue heterogeneity. The manuscript is of interest to groups focused on integrating omics data to understand tumor microenvironments.

Major points:

The manuscript needs major revisions in view of presentation and argumentation both for the figures and text. In its current state, it is very hard for the reader to focus on scientific findings or the concept of the developed workflow because both figures and text are overloaded with details that do not contribute to the story line of the paper.

- *Figure presentation:*

To enhance clarity and maintain focus on the main message, it would be helpful to streamline the figures by moving secondary information to the Supplementary. For example in Figure 1, we suggest enlarging panel (a) to emphasize the workflow while relocating panels (b) and (c) to the Supplementary. The icon representing the liver tumor currently in (b) could be incorporated directly into (a) as the starting point for the LC-MS/MS analysis. Similarly, we recommend placing greater emphasis on panels (d) and (f) while moving (e) and (g) to the Supplementary.

1a. To make Figure 1 more focused and legible, we have

- Enlarged the workflow overview panel in **Fig 1a** and relocated the liver tumor icons to the beginning of the panel, and
- Enlarged the metabolomics and transcriptomics profile plots in **Fig. 1b** and **c** (previously Fig. 1d and f).
- Moved the tumor photos and sample distribution graphs to **Appendix Fig. S1,**
- Moved the dot plots showing RNA-seq biological and metabolic process enrichments to **Expanded View Fig. EV1**, and
- Moved the bar charts showing distributions of changed transcriptomic or metabolomic profiles across HCC stages to **Appendix Fig. S4**.

Figure 2 would also profit from reducing the number of individual data elements and focusing on the main findings from the metabolomics side. The images of the H&E are hardly visible in g), maybe one section could be presented in more detailed instead of presenting six sections the same time. Information in h) is somewhat repetitive to 1c). Elements i), j) and k) do not add any elemental information and can be summarized briefly in the text or moved to the Supplementary.

1b. To make Figure 2 more focused and legible, we have

- Removed the H&E image (previously Fig. 2g) because the zoom-in views of the ROIs have already been shown in **Fig 3**,
- Moved the scatter plots showing the correlations between DESI and LC-MS for the two selected metabolites (previously Fig. 2m and n), the differentially expressed metabolites between tumor and normal sections (previously Fig. 2o), and example DESI images for the two selected metabolites (previously Fig. 2p) to **Expanded View Fig. EV2**,
- Moved the bar chart showing the distribution of tumor samples subjected to DESI-MSI (previously Fig. 2h) to **Appendix Fig. 1c**,
- Removed the process diagrams (previously Fig. 2i and j) because they have been described in the text already (**Methods**), and
- Enlarged all the figure panels.

The other figures, especially Figure 6, would also benefit from a more focused presentation by reducing the number of individual data elements.

1c. To simplify and re-organize other figures, we have

- Moved the heatmap and clustergram showing the abundance levels of highly-abundant putative metabolites at the ROIs of DESI images (previously Fig. 3e) to **Expanded View Fig. EV3**,
- Removed the SgME maps for HEP0321 and HEP0277 (in Fig. 6a) because they are redundant and already shown in **Appendix Fig. S10**,
- Moved the scatter plots showing the changes in the abundance levels of the metabolites from the six metabolite clusters between tumor and normal tissue sections (previously Fig. 6g) to **Appendix Fig. S11**.

The manuscript is at present largely organized by figure. It would be essential to the success of the manuscript to improve the story line of the manuscript by reducing the amount of descriptive details and instead discuss all main elements of the figures in detail (after revising the figures and distilling to main findings). Right now, the text is descriptive and does not succeed in coming to broader scientific conclusions. For example, in the paragraph titled "LC-MS and DESI-MSI abundance levels of highly abundant metabolites are positively correlated" each sentence refers to a single panel from Figure 2 creating a somewhat repetitive and list-like structure. We recommend to discuss main findings in more detail and move background information that does not add to the key understanding of clinical findings or the established workflow to the Supplementary.

1d. We have revised the Results section by moving the more descriptive sentences to the Methods section (including **lines 22-25, pg. 27**; **lines 9-14, pg. 30**; and **lines 2-4, pg. 34**).

1e. To make the motivations of the correlation analyses clearer, we have added the following new sentence to Results (**lines 20-23, pg. 10**):

> "If these matched peaks correspond to the same metabolites, we expect their bulk LC-MS and averaged DESI-MSI abundance levels to be positively correlated to each other across all the tissue sections".

1f. To provide more biological information about the found highly-abundant metabolites, we have added the following new sentences to Results (**lines 9 – 18, pg. 11**):

> "PC(18:0/20:4) contains stearic acid (18:0) and arachidonic acid (20:4) at the sn-1 and sn-2 positions. PC and phosphatidylethanolamine (PE) are the two most abundant phospholipids in the plasma membranes of all mammalian cell types, and changes in the molar ratio between PC and PE has been linked to metabolic dysfunction-associated steatotic liver disease (MASLD) in animal models and humans (van der Veen *et al*, 2017). Cholesterol sulfate is synthesized via the sulfonation of cholesterol by the cholesterol sulfotransferase SULT2B1b and can be found in many types of biofluids and tissues, including liver (Strott & Higashi, 2003). Elevated levels of plasma cholesterol sulfate was found in patients with liver cirrhosis and hypercholesterolemia (Tamasawa *et al*, 1993)."

1g. We have significantly revised the COMET Path analysis procedures and are able to identify more enriched biological processes and also had clearer interpretations of the biology of the six MERs. For more detailed information, please refer to our **Responses 2g and j** to Reviewer #3.

- *<u>Nomenclature of metabolites:</u>*

The manuscript would benefit from using a consistent and standardized nomenclature for metabolites. For instance, in Figure 2 panel (b) uses metabolite names, panels (m) and (n) use both names and internal abbreviations while panel (p) uses abbreviations only. It would be beneficial to refer to the metabolites in a uniform fashion ideally following established conventions e.g. LipidMaps. Currently, glycerophospholipids are not named coherently throughout the manuscript. Sometimes they are referred to by abbreviation (PC(20:4/16:0)) and sometimes with full name (1-stearoyl-2-arachidonoyl-sn-glycero-3-phosphocholine, SAPC) for example in Figure 4 and throughout the text. Similar considerations apply to other lipid annotations throughout the manuscript. Publications on best practices in MS-based lipidomics may provide further guidance on this point.

1h. We have changed the names of the lipids according to standard abbreviation throughout the text and figures. For example, SAPC is now consistently being called as PC(18:0/20:4) in our manuscript.

We understood the manuscript that only 11 metabolites in total are annotated manually and in full ID, this is a very low number from 3 different runs per sample. How is an interpretation possible together with transcriptomics data if the coverage of metabolism is that different?

1i. There are 230 metabolites putative annotation using MS^2 (**Appendix Table S2**). The manual assignment was only checked for eleven of the metabolites. The reason for this is that only these eleven metabolites were highlighted and discussed in the manuscript.

- *Meaningfulness of the developed workflow:*

The presented workflow enables to predict composition of bulk samples after training regression models on spatial metabolomics and histological data. We acknowledge this as an achievement of the authors but question the clinical relevance for diagnostics in a clinical setting. The manuscript offer no insights in how this may benefit HCC diagnostics / research. Its not clear at the moment how a clinical application is implemented and is limiting the potential of this study to inspire other researchers.

1j. We thank the reviewer for this valuable comment highlighting the importance of clinical relevance. While the primary objective of this study was to establish a proof-of-concept methodological framework for integrating spatial metabolomics and histology to predict bulk tissue composition, we recognize that outlining its translational potential, particularly in biomarker discovery, would strengthen the work. We have added the following sentences near the end of Discussion (**lines 24-27, pg. 25, and lines 1-2, pg. 26**):

> "Our framework could eventually enable systems-level (transcriptomics-proteomics-metabolomics) understandings of the roles of local cellular niches in cancer metabolism and tumorigenesis. This holds the promise of biomarker discovery and subsequent patient stratification for disease treatments and managements in HCC and potentially other cancer types. In future studies, we plan to evaluate the predictive performance of this workflow on clinically annotated HCC therapeutic cohorts and assess its potential to unravel predictive biomarkers."

1k. After revising the COMET's Path procedures and results, we also found that most top correlated genes are potential HCC markers. We have added new disease-free survival curves for two top correlated genes. These new results also demonstrate potential clinical relevance or applications of our framework. For more detailed information, please refer to our **Response 2g** to Reviewer 3's comments.

Minor Points:

- *Metabolomics: Little information is provided on the exact parameters of LC-MS/MS analysis. More details and acquisitions settings should be included in a revised form of the manuscript.*

1l. We have added additional details particularly regarding LC–MS/MS acquisition settings (**pg. 29**), which includes

- Column specifications
- Autosampler conditions
- ESI source parameters such as spray voltages, desolvation temperatures and gas flow settings
- Complete gradient programs with runtime, wash, and equilibration/post-run equilibration steps

- *Abbreviations: Many abbreviations are used throughout the manuscript (both text and figures) and limit the flow of the text. Consider reducing the number of abbreviations both for the established workflow and identified metabolites/biomarkers.*

1m. We have reduced many abbreviations and used their full words to make the manuscript easier to read and follow. Specifically,

- "SgMERDeconv" has been removed from the whole manuscript and figures,
- "IntraTH" and "InterTH" have been replaced with "intra-tumor heterogeneity" and "inter-tumor heterogeneity", respectively,
- ""PM" has been replace with "putative metabolites" in the main text,
- "ML" has been replaced with "machine learning", and
- "STx" has been replaced with "spatial transcriptomics".

- *Technical details: We appreciate the thorough documentation of the workflow and analyses but they somewhat interrupt the flow of the text. For example in paragraph 2 ("Different liver cancer..."), the exact mass range for LC-MS/MS analysis and the number of individual sections analyzed may be presented in Methods/Supplementary. Similar recommendations apply to other parts of the text.*

1n. To make the section more readable, we have

- Moved the sentences on the number of tissue sections used for different omics analyses to Methods (**lines 22-25, pg. 27**),
- Moved the sentences on the exact mass ranges to Methods (**lines 9-14, pg. 30**), and

- Moved the sentence on the omission of an outlier sample to Methods (**lines 2-4, pg. 34**)

- *P3, L11-14 sentence L12 makes little sense L14 irreproducibility is like a technical bias - but the papers state different outcomes of studies L18 intact tissue- biopsy?*

1o. We have provided more examples to explain the point clearer and removed the word "irreproducibility" because we are not referring to technical bias or irreproducibility. The revised sentences now become (**lines 12 – 18, pg. 3**):

> "Thus, metabolites found in bulk tumors may originate not only from cancer cells but also other non-cancerous cells, such as normal epithelial, stromal, or immune cells. Furthermore, these metabolites may indicate not only tumorigenesis but also non-tumorigenesis processes, such as steatosis, fibrosis, necrosis, xenobiotic metabolism, or other processes. These intra- and inter-tumoral heterogeneities may lead to the identification of potential metabolic markers that are highly discriminative in specific cohorts, but not generalizable to other cohorts or larger populations (Struys et al, 2010; Jentzmik et al, 2010; Sreekumar et al, 2009)."

- *P4 - L1 identification of lipids with MS1 only is anyways not correct based on standard reporting procedures, cite also those and the bottleneck of MS2 missing in large extend from MSI*

1p. We have revised the sentences to become (**lines 4-8, pg. 4**):

> "Furthermore, MSI data alone is insufficient and MS/MS data is required to fully annotate the detected mass features (Köfeler *et al*, 2021; Alseekh *et al*, 2021; Sumner *et al*, 2007). Many current MSI-based studies do not directly generate MS/MS data from MSI, and instead use LC-MS/MS on separate tissue sections to annotate metabolites (Hendriks *et al*, 2024; Sun *et al*, 2023; Zhu *et al*, 2022)."

The following three new references were cited:

- Sumner LW, Amberg A, Barrett D, Beale MH, Beger R, Daykin CA, Fan TW-M, Fiehn O, Goodacre R, Griffin JL, *et al* (2007) Proposed minimum reporting standards for chemical analysis. *Metabolomics* 3: 211–221

- Köfeler HC, Eichmann TO, Ahrends R, Bowden JA, Danne-Rasche N, Dennis EA, Fedorova M, Griffiths WJ, Han X, Hartler J, *et al* (2021) Quality control requirements for the correct annotation of lipidomics data. *Nat Commun* 12: 4771
- Alseekh S, Aharoni A, Brotman Y, Contrepois K, D'Auria J, Ewald J, C. Ewald J, Fraser PD, Giavalisco P, Hall RD, *et al* (2021) Mass spectrometry-based metabolomics: a guide for annotation, quantification and best reporting practices. *Nat Methods* 18: 747–756

- *No LCMS data in data repository found after locking in - this needs to change*

1q. We have uploaded the following raw data to respective public data repositories:

**Data**	**Data repository**	**Accession number / Links**
LC-MS and DESI-MSI	EMBL MetaboLights	MTBLS13432<br>*Private reviewer link:*<br>https://www.ebi.ac.uk/metabolights/reviewer73acd5d8-bee2-4adf-92d7-f95d38d53d4d
10X Visium	NCBI Gene Expression Omnibus	GSE312327<br>https://www.ncbi.nlm.nih.gov/geo/query/acc.cgi?acc=GSE312327<br>*Private token for reviewer access:* efsviuokxfknbsx
H&E images	ImmunoAtlas	https://immunoatlas.org/CCPA/251202-1<br>https://immunoatlas.org/CCPA/251202-2

Specific Responses to Reviewer #3's comments:

This manuscript presents Spatially-guided MEtabolomics (SgME) profiling, an integrative framework combining LC-MS/MS, DESI-MSI, histopathology, and RNA-seq to delineate intra-tumoral heterogeneity (IntraTH) in primary liver cancers. The authors apply their approach to 117 samples from 26 patients, generating "metabolic region (MER)" maps and training regression models to deconvolve bulk metabolomics into spatial components.

The topic is timely and important: the reconciliation of bulk metabolomics with spatially resolved MSI is a major methodological gap in cancer systems biology. The scale of the dataset (multi-region, multi-modal) is commendable, as is the effort to link metabolites with histological features and transcriptomic pathways. However, while the approach is innovative, several conceptual, methodological, and validation issues limit the current impact. Clarification and strengthening are required before this work can be considered for publication in Molecular Systems Biology.

Major Comments

1. *Markers of metabolic regions (MERs).*

Markers of metabolic regions represent a key information allowing the validate the data, the data analysis pipeline, and the results. Some additional evidence would be appreciated to address the following concerns. First, some of the markers are not common lipids and include fatty acyl chains with odd carbons: PC(19:0/11:0), PI(17:1/20:2), TG(15:0/16:1/16:0) etc. Such lipids are not common in mammalian cells and additional evidence is needed to validate the correctness of their structural identities. The presented MS/MS spectrum for PI(17:1/20:2) in FIg S6 is not convincing as it shows C19:2 as the highest fragment. MS/MS spectra for other uncommon lipids are not shown.

2a. We thank the reviewer for checking our assignments so carefully. We have re-checked the MS2 spectra by an independent person and noted there were three mistakes:

- PI(17:1/20:2) should be PI(17:0/20:3),

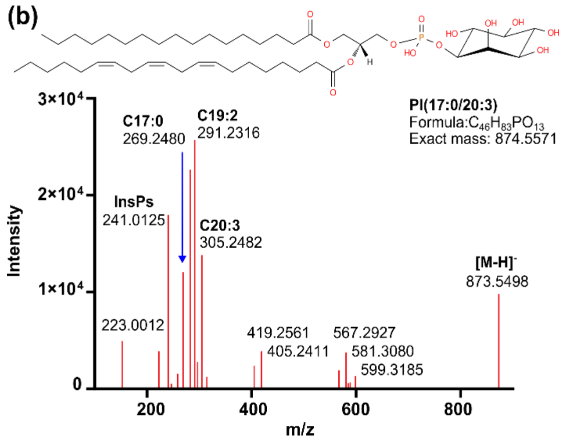

- Laudanosine's formula should be C21H27NO4 and theoretical m/z should be 358.2013, and

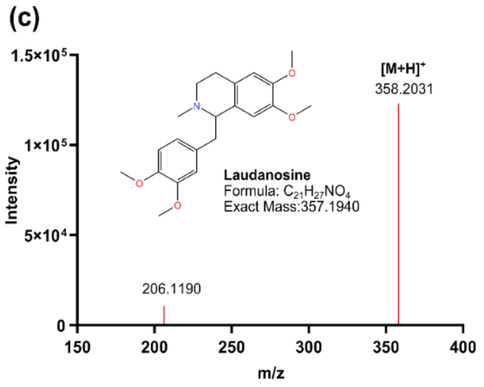

- P120 should be PC(16:0/20:4) rather than PC(20:4/16:0).

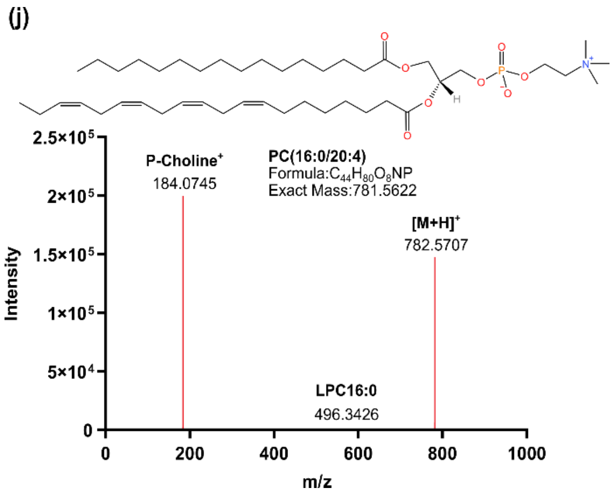

They are all corrected in the revised **Appendix Fig. S8** and **Table S3**.

Second, there is a number of non-endogenous metabolites identified, including laudonosine, tocopherol, and tocotrienol. Please demonstrate LC-MS/MS results for their identities. The presented MS/MS spectra in Fig S6 for laudanosine and tocopherol are not convincing as their fragments are not annotated and there are no spectra of authentic standards.

2b. Laudanosine is not a standard compound, but a metabolite of the neuromuscular-blocking drugs atracurium and cisatracurium. In surgery, we had used atracurium as an anaesthesia, thus it is not surprising to find the compound in the samples. We have revised the MS/MS plot for the compound in Appendix Fig. S8 (please find the plot at **Response 2a**).

We also added the following sentences to the Results section (**lines 20-27, pg. 13**).

> "Surprisingly, we also found some non-endogenous metabolites, such as laudanosine, a metabolite of the neuromuscular-blocking drugs, atracurium and cisatracurium (Fodale & Santamaria, 2002), which were typically used as muscle relaxants during the liver surgeries of our patients. These non-endogenous metabolites are "discriminative" in our study maybe because tumor tissues have different xenobiotic metabolism capabilities than normal tissues (Fig. EV1). However, these metabolites are not related to tumorigenesis and thus should not be used as HCC biomarkers. The results show the high sensitivity of mass spectrometry imaging and also the importance of accurate metabolite annotations during spatial metabolomics profiling of human tumor tissues."

2c. We understand the reviewer had doubt on the assignment of tocopherol, but we had matched the fragments. A revised plot with fragment annotations is included in **Appendix Fig. S8**.

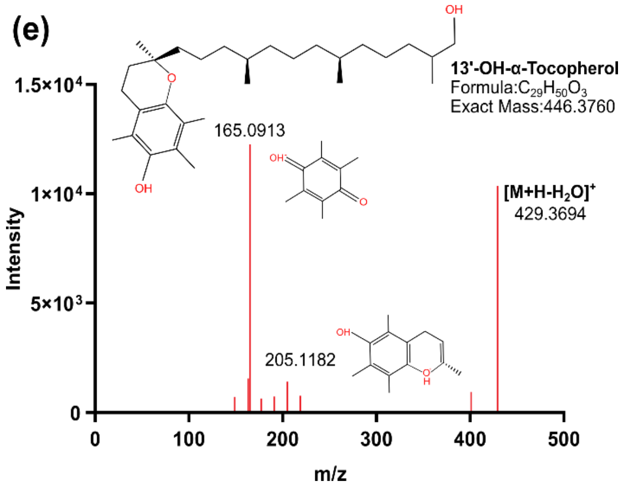

Moreover, the authors should explicitly state MSI annotation confidence levels and discuss risks of mis-annotation.

2d. There is a four-tier confidence levels rule for metabolite or compound identification, as defined by the Metabolomics Standard Initiative (Sumner *et al*, 2007). The assignments we made in the manuscript were all at level II, which is based on M/Z ratios and matched MS/MS from external databases or publications.

We have included the following sentence about our metabolite identification confidence level and cited the Metabolomics Standard Initiative reference in the Discussion section (**lines 19-21, pg. 25**):

> "Also, our metabolite annotations were based on m/z and MS^2, which is confidence level II (Sumner *et al*, 2007)."

2. Definition and novelty of MERs

The proposed "metabolic regions (MERs)" are defined by integrating LC-MS-identified metabolites with MSI and histopathology. It remains unclear whether MERs represent new biological entities or simply reflect re-labeled histopathological categories. The claim that MERs cannot be identified by clustering alone should be better substantiated.

2e. Metabolic regions (MERs) are defined by the discriminative putative metabolites that we identified through building supervised classifiers based on mass spectrometry images (MSI). Thus, MERs are defined molecularly by the mass features and not the histological features of the regions. We have added a sentence to clarify this (**lines 7-9, pg. 7**).

> "The histological images are used only to provide the labels for training, and MERs are defined molecularly by the mass features from MSI images and not the histological features from H&E images."

We already have an example to show that MERs may overlap in the original manuscript (sample B0003-N in **Fig 6b**). We have added the following paragraph to **pg. 19:**

> "Furthermore, the full SgME maps confirmed our hypothesis that MERs may overlap on top of each other. For example, we found a ME-steatotic region that overlapped

with a ME-normal region in tumor B0003 (lower panels, Fig. 6). Both regions were automatically assigned by our trained PLS-DA classifiers. The DESI images of selected discriminative metabolites, DG(16:0/18:1) and lysoPC(18:1), also show the same trends (tumor B0003, last two rows, Fig. 4g). From the corresponding H&E image, we confirmed that the predicted SgME maps were accurate because lipid droplets could be observed in the predicted ME-steatotic region, while normal hepatocytes could be observed in the predicted (white triangles, lower panels, Fig. 6). Many such instances of overlapping MERs can be found from the full SgME maps for all samples (Appendix Fig. S10). Importantly, ME-steatotic regions do not always overlap with ME-normal regions (for example, sample HEP0152, last two rows, Fig. 4g). If pure spatial clustering of the DESI images were used to identify MERs, many fragmented clusters (e.g., ME-steatotic-only, ME-normal-only, ME-steatotic-plus-normal, and possibly many other combinatorial clusters) would have been found, thereby making downstream consolidations and interpretations of these regions more challenging."

3. *Integration with transcriptomics (COMET's Path analysis)*

The correlation-based integration of metabolite abundance with bulk RNA-seq is interesting, but risks spurious associations given the small sample size per modality and high dimensionality. No multiple-testing correction across metabolite-gene correlations is described.

2f. All of our analyses were subjected to rigorous multiple-hypothesis testing correction following the standard Benjamini-Hochberg (BH) procedure. "Padj" refers to adjusted P-values after BH's correction. This is mentioned multiple times in the Methods section and the figure legends (such as **Figs. 1, 2, 5,** and **7**).

To avoid ambiguity, we have added an citation to Benjamini-Hochberg's original paper (Benjamini & Hochberg, 1995) under Methods in **lines 6-9, pg. 37**. The sentence now becomes:

"Only those genes with significantly positive correlations with at least 50% of the discriminative metabolites were retained (Spearman's test; Padj < 0.05; all P-values were adjusted using the BH procedure) (Benjamini & Hochberg, 1995)."

Padj < 0.1 seems permissive and goes against the state of the art. The authors should present the results with more statistically stringent selection (pvalue<0.05, multiple-testing correction) and discuss the differences.

2g. We are really grateful for this important feedback from the Reviewer and agree that a threshold of 0.10 was too lenient. In the revised manuscript, we have reduced the Padj threshold to 0.05. As a result of using the new threshold, the numbers of significantly correlated genes had dropped by ~26% to 79% (new **Fig. 5d**). However, we found that <u>most of the revised significant top-correlated genes have become potential HCC biomarkers</u>, such as *LSM4*, *RBM24*, *PLAUR*, *AP1M2*, *TERT*, *BCL9*, *HSD17B4*, and other genes (new **Fig. 5d**).

MERs	Exemplerary PMs	Corr. RNAs (Padj < 0.05)	Five most correlated RNAs (in decreasing order)
Low-grade	PC(18:0/20:4)	67	***LSM4****, *AURKB**, *SAMMSON**, *ZDHHC12-DT**, *P2RX4**
High-grade	PG(18:0/18:2)	18	***RBM24****, *TXNRD1**, *IRAK2**, *TTC9*, *LGALS4**
Necrotic	13-OH-alpha-Tocopherol	231	*IGHV1-46*, *IGKV4-1**, *PLAUR**, *SLC16A9**, *TMEM51**
Fibrotic	Cholesterol sulfate	342	*AP1M2**, ***TERT****, *DIAPH3**, *DUXAP9**, *CENPU**
Steatotic	PC(18:0/20:2)	65	*BCL9**, *LINC02607*, *LOC102723544*, *MBNL3**, *GAGE1**
Normal	PE(16:0/18:2)	14	***HSD17B4****, *HNF4A-AS1**, *NCOA4**, *MPDZ**, *SALL1**

* = Known potential HCC markers or associations, **Bold** = STx visualization

We have revised the whole sections (**pg. 15 and 17**) and they now become the following:

"To further investigate and interpret the biological processes that may drive the discriminative metabolites in different MERs, we determined the significantly correlated genes for each of the six MERs (P_{adj} < 0.05, Spearman's rank correlation test; **Fig. 5d**). Interestingly, we found that almost all of the top-most significantly correlated genes have been previously found to be potential HCC markers or associated with HCC (**Fig. 5e**). Many of them are differentially expressed between tumor and adjacent-normal regions in our cohort (**Fig. 5f**). For some of these potential HCC markers, their elucidated functions or mechanisms agree with COMET's Path's assignment of metabolic regions. For example, telomerase reverse transcriptase (*TERT*) promoter mutation is an early somatic genetic alteration associated with the transformation of cirrhosis (advanced stage of liver fibrosis) to HCC (Nault *et al*, 2014; Müller *et al*, 2020). COMET's Path

correctly assigned the gene to the ME-fibrotic region. Another example is B-cell CLL/lymphoma 9 (BCL9), which activates Wnt/β-catenin signaling pathways (Gay *et al*, 2019) that modulate lipid formation in MASLD and HCC (Wang *et al*, 2023). COMET's Path also correctly assigned the gene to the ME-steatotic region."

"Importantly, many of these top-correlated genes were discovered as potential HCC markers via large-scale bioinformatics analyses, and most of them have unknown, unclear, or even conflicting previous reports on their functions or roles in HCC. By using SgME profiling and COMET's Path analysis, we were able to assign them to specific metabolic regions or metabolites and hypothesize their potential roles in HCC. The first example is the Like-Smith (LSM) protein family, a group of RNA-binding proteins involved in RNA metabolism. In a previous integrative bioinformatics analysis based on multiple clinical databases, *LSM4* was found to be overexpressed in HCC and a strong diagnostic marker (AUC ~0.9) (Chen *et al*, 2022). COMET's Path found that *LSM4* is the most correlated RNA with the ME-low-grade region, including PC(18:0/20:4) (**Fig. 5b**), and thus the gene is likely to be involved in the tumorigenesis process and a strong HCC marker candidate. The second example is the urokinase-type plasminogen activator receptor (uPAR) encoded by *PLAUR*, which was found to be a potential HCC invasiveness and prognosis marker (Zheng *et al*, 2000), but the precise roles of the receptor in HCC are unclear. COMET's Path assigned *PLAUR* to the ME-necrotic region, agreeing to its known roles in extracellular matrix remodeling and known inductions by hypoxia and inflammation (Graham *et al*, 1998; Smith & Marshall, 2010), Our results suggest that the receptor may involve in repairing the injured regions in or around necrotic regions within tumors, and thus may not be a predictive marker for tumorigenesis. The third example is RNA-binding motif protein 4 (RBM4), which is a post-transcriptional regulator that promotes cell differentiation by modulating alternative splicing and translation of target genes. In some studies, RBM4 was found to be anti-tumor with low expressions indicating more aggressive HCC and poorer prognosis (Chen *et al*, 2017; Wang *et al*, 2014); while in other studies, RBM4 was found to be pro-tumor with animal knockdown resulting in reduced liver tumor growth and angiogenesis (Han *et al*, 2023). COMET's Path found that *RBM4* is the most correlated with the metabolites from the ME-high-grade region, including PG(18:0/18:2) (**Fig. 5b**). Thus, the molecule is unlikely to be directly involved in tumorigenesis, but may be involved in immune responses or activations (Gowen *et al*, 2015). In conclusion, COMET's Path is a very powerful method to use the discriminative metabolites found by SgME classifiers to

help us to interpret and prioritize potential HCC markers for further mechanistic or clinical validations."

Spatial transcriptomics validation is limited to two tumors only. The authors should discuss the limitations and biases of this approach.

2h. We have added the following sentences to Discussion (**lines 16-19, pg. 25**):

"Another limitation of our current study is the small number of samples tested using spatial transcriptomics. Follow-up studies based on more tissue samples from different disease stages are needed to confidently map out the spatial localization patterns of the found highly-correlated genes using multiplex protein-marker imaging or spatial transcriptomics assays."

4. Machine learning models and generalization

Regression models predicting MER composition from bulk LC-MS are promising, but performance metrics (R^2 up to 0.93) seem unrealistically high. Overfitting risk should be explicitly addressed.

2i. We have added the following sentences to Results (**pg. 22**):

"Although these high R^2 values were very encouraging, they may reflect some degree of overfitting and should therefore be interpreted with appropriate caution. The true generalizability of the models will require confirmation in independent external cohorts."

5. Biological insights and clinical relevance

The manuscript identifies clusters of metabolites (e.g., low-grade+/necrotic-) that would be invisible in bulk profiling. This is a strong result. However, their biological significance (drivers of tumor progression, therapeutic vulnerability) is not fully developed.

2j. We have changed to use Gene Set Enrichment Analysis (GSEA) (Subramanian *et al*, 2005), which requires only the full list of query genes to be sorted by their correlations to the discriminative metabolites from a MER, and does not require any thresholding or filtering of the query genes. Consequently, we are able to identify more enriched biological processes and also had clearer interpretations of the biology of the six MERs (new **Fig. 5c** and **Appendix Fig. S9**).

The following paragraph has been added to the Results (**lines 4-22, pg. 15**):

> "Importantly, we also found that most of these biological processes agree with our definitions of MERs. For example, discriminative putative metabolites of the ME-normal regions are correlated with genes involved in core metabolic functions of normal livers, such as lipid and fatty acid metabolism, catabolic, and biosynthetic processes. We also found that putative metabolites of the ME-low-grade and -steatotic regions are correlated with RNAs involved in cell cycles, divisions, and proliferation, which are the characteristics of hepatocytes during tumorigenesis or steatosis (Hall *et al*, 2021). Unexpectedly, we found that the metabolites in the ME-high-grade regions are quite different from those in the ME-low-grade regions and correlated with RNAs involved in adaptive immune responses, including activations of B-cell receptors (BCR), high-affinity IgE receptors (FcεRI), and the complement systems. Our results suggest that in ME-high-grade regions, the transformed hepatocytes may slow down their proliferation and metabolism functions, while B cells may produce antibodies that form immune complexes with tumor antigens, activate the complement pathways, and drive chronic inflammation, angiogenesis, and T-cell suppression in HCC (Xiao *et al*, 2022). Interestingly, these pathways were also enriched in the ME-necrotic and -steatotic regions, suggesting these three MERs may overlap or close to each other in the tumor tissues. Finally, we found that leukocyte activation and migration and vasculature development were enriched only in the ME-fibrotic and -steatotic regions, agreeing with known roles of T-cell and other lymphocytes and angiogenesis in liver fibrosis and fatty livers (Hammerich & Tacke, 2023; Peiseler *et al*, 2022)."

2k. For clinical relevance, we found that almost all of the top-most significantly correlated genes have been previously found to be potential HCC markers or associated with HCC. We have added disease-free survival curves for two of the top correlated genes, *LSM4* and *RBM24* (new **Fig. 5e**), based on TCGA HCC datasets for 372 patients. Both of these genes have significant hazard ratios (1.842 and 2.108) with 95%CI higher than 1. Thus, they are potential HCC markers.

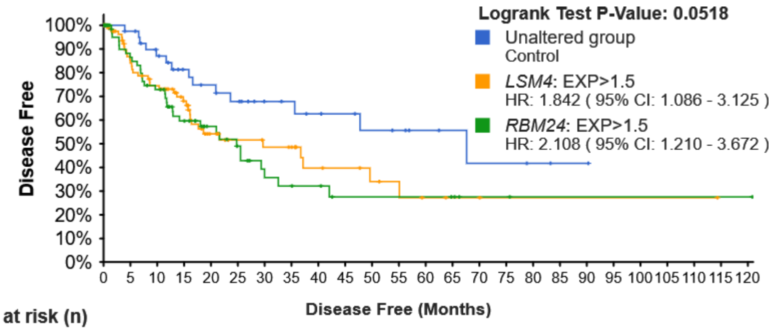

Number at risk (n)

Unaltered group	40	39	32	25	22	19	14	13	11	10	8	7	5	4	3	3	2	1	1	0	0	0	0	0	0
LSM4: EXP>1.5	87	62	53	38	23	18	16	15	9	8	6	5	3	2	2	1	1	1	1	1	1	1	1	0	0
RBM24: EXP>1.5	68	51	42	30	21	16	10	9	8	5	5	5	5	4	2	2	1	1	1	1	1	1	1	1	1

2l. We also added new scatter plots to show that the significantly positive correlations between the RNAs of these two genes and the discriminative metabolites from the ME-low-grade and -high-grade regions, respectively (new **Fig. 5b,** both P<0.05).

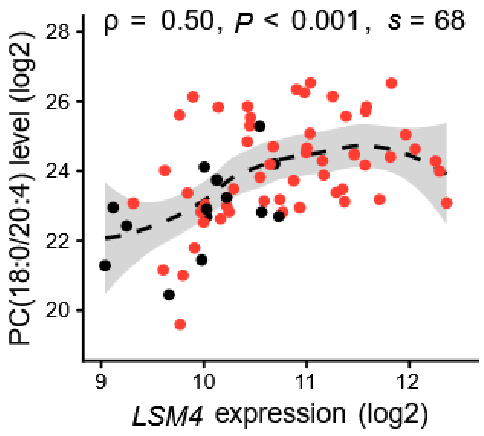

6. <u>Positioning relative to existing literature</u>
The authors should more clearly contrast SgME with other integrative frameworks and methods. The degree of conceptual novelty is somewhat overstated.

2m. The objective of SgME is to integrate <u>the mass separation capabilities of LC-MS/MS and the spatial separation capabilities of mass spectrometry imaging (MSI)</u> to identify and annotate putative metabolites in heterogenous tumor tissues. Our approach first annotates peaks using LC-MS/MS and then determines the spatial localization of these putative metabolites using MSI.

We have already provided a review of current MSI data analysis and integration approaches and their limitations (**pg. 4**), and how SgME overcome these limitations (**pg. 5**). Briefly, most existing MSI-based studies followed an opposite approach from SgME, in which they first identified peaks

using MSI and then tried to annotate these peaks using LC-MS/MS. The following three recent papers that used such an opposite data integration approach have been cited in our Introduction (**pg. 4**):

- Hendriks TFE, Krestensen KK, Mohren R, Vandenbosch M, De Vleeschouwer S, Heeren RMA & Cuypers E (2024) MALDI-MSI-LC-MS/MS Workflow for Single-Section Single Step Combined Proteomics and Quantitative Lipidomics. *Anal Chem* 96: 4266–4274
- Sun C, Wang A, Zhou Y, Chen P, Wang X, Huang J, Gao J, Wang X, Shu L, Lu J, *et al* (2023) Spatially resolved multi-omics highlights cell-specific metabolic remodeling and interactions in gastric cancer. *Nat Commun* 14: 2692
- Zhu Y, Zang Q, Luo Z, He J, Zhang R & Abliz Z (2022) An Organ-Specific Metabolite Annotation Approach for Ambient Mass Spectrometry Imaging Reveals Spatial Metabolic Alterations of a Whole Mouse Body. *Anal Chem* 94: 7286–7294

5th Feb 2026

Manuscript Number: MSB-2025-13233R
Title: Spatially-guided metabolomics profiling of heterogeneous human tumor tissues
Author: Jia-Ying Lee
Jingtao Zhang
Sin-Chi Chew
Shihleone Loong
Liang Xu
Jia-Wen Kong
Fedor Grigoryev
Alexander CHUNG
Jin-Yao Teo
Peng CHEOW
Glenn Bonney
Brian GOH
Wei-Qiang Leow
Yulan Wang
Lit-Hsin Loo
Pierce CHOW

Dear Dr Loo,

Thank you for submitting your revised manuscript. First, I apologize for the delay in the review process. This was due to the holiday season and the unavailability of the original reviewers, which required us to invite two new reviewers.

We have now received feedback from the new reviewers, who were asked to evaluate your response to the concerns raised in the previous round of review. As you will see below, the reviewers find that the revision has improved the manuscript; however, several -relatively minor- issues remain and should be carefully addressed.

Referee #4 indicates that further revision is needed to better clarify the main findings and to provide a clearer definition of MERS. Referee #5's comment should be addressed either by repeating the training as suggested or through appropriate textual revisions.

On a more editorial level, please address the following points:

1. "Disclosure statement" should be renamed to "Disclosure and competing interests statement".

2. Data availability:
- "Code Availability" should be included under "Data Availability," and the separate "Code Availability" heading should be removed.
- Please remove"Reviewer Access" and make sure the datasets will be made publicly available upon the acceptance of the manuscript.

3. Author checklist:
- Please enter the corresponding authors' names.
- The response under "Computational Model" in the Data Availability section is missing; please select a response.

4. Please download and fill our Reagents and Tools Table template (.docx), which you can find in our author guidelines: https://link.springer.com/journal/44320/submission-guidelines#structuredmethods. The current version should be removed.

5. Appendix
- Appendix figures should be listed individually in the Table of Contents with their corresponding page numbers.
- Appendix Tables S1a and S1b should be renamed to Appendix Tables S1 and S2, and all subsequent Appendix tables should be renumbered accordingly, with updated callouts throughout the manuscript.

6. Source Data: Source data files should be organized by figure, with all files for each figure saved in a single folder. Each folder should then be compressed into a .zip file. For example, all source data files for Figure 1 should be saved in one folder, zipped, and uploaded as "SD_Figure_1.zip."

7. During our routine image integrity checks, we observed that the images within the Appendix Figure S10 appear pixelated

under analysis. This is often a result of converting original 16-bit TIFF files to RGB format for publication. While this is not inherently problematic, it can give the impression of image alteration to critical readers.

To address this, please upload the Appendix file in a higher resolution. If it is not possible to reproduce the Appendix at higher resolution, we recommend instead uploading the original blot source data with your online submission or depositing the raw files on BioStudies https://www.ebi.ac.uk/biostudies/sourcedata/studies and including the archive accession number in your Data Availability section. This will enable us to confirm the integrity of the complete figure set and enhance transparency for readers.

8. During our routine image integrity checks, potential figure reuse was identified between the following panels. Please review these panels, and if reuse is confirmed, it should be explicitly indicated in the figure legends:
- Possible Figure reuse between Figure 2E and Figure 3D
- Possible Figure reuse between Figure 3D and Appendix S10
- Possible Figure reuse between Figure 6A and Appendix S10

9.Please address the followiing issues related to figure legends:
- Please note that the exact p values are not provided in the legends of figures 7C, F; EV2 A
- Please indicate the statistical test used for data analysis in the legends of figures 5F, 7B
- Please note that information related to n is missing in the legends of figures 7C, F

10. Sections need to be named and the order should be corrected: Title page - Abstract - Keywords - Introduction - Results - Discussion - Methods - Data Availability - Acknowledgements - Disclosure and Competing Interests Statement - References - Figure Legends - Table(s) - Expanded View Figure Legends.

As a matter of course, please make sure that you have correctly followed the instructions for authors as given on the submission website.

Thank you for submitting this paper to Molecular Systems Biology.

Kind regards,
Jingyi

Jingyi Hou, PhD
Senior Editor
Molecular Systems Biology

*** PLEASE NOTE *** As part of the EMBO Press transparent editorial process initiative (see our Editorial at https://dx.doi.org/10.1038/msb.2010.72 , Molecular Systems Biology will publish online a Review Process File to accompany accepted manuscripts. When preparing your letter of response, please be aware that in the event of acceptance, your cover letter/point-by-point document will be included as part of this File, which will be available to the scientific community. More information about this initiative is available in our Instructions to Authors. If you have any questions about this initiative, please contact the editorial office (msb@embo.org).

Reviewer #4:

This is not a full review of the manuscript, but rather a specific evaluation of whether the revised manuscript has adequately responded two original reviewers' (Reviewer #2 and #3) comments as requested by the editor.

The issues raised by Reviewer #2 can be roughly summarized as two parts. First, the study is largely descriptive and it is unclear what are the new findings. Second, the presentation is a bit unfocused and the figures and text should be reorganized. In this revision, the authors have done a good job improving the quality of presentation, thereby sufficiently addressing the second criticism. On the other hand, additional revision is needed to further clarify what are the main findings. It is until the Discussion section did the authors summarize their main findings, leaving the readers in suspension while reading the majority of the manuscript. It would be helpful if the authors could also including summary statements in the Abstract and Introduction sections and, when appropriate, further provide motivating discussions in this section to guide the readers.

Reviewer #3 raised significant concerns regarding the definition of metabolic regions (MERs), and questioned the validity of using some of the noncommon markers for MER identification. This is an important concern, since MER-based analysis is central to this study. Unfortunately this reviewer does not have the expertise to evaluate if the explanation and additional analysis presented in this revision are sufficient to address this concern.

In addition to Reviewer #3's original comments, it appears that an exact definition of MER is missing. While a large portion of the manuscript is dedicated to the comparison of different MERs, it is unclear which properties are different because they are used to define the MERs and which are additional characteristics through integrative analysis. It would be essential for the authors to clearly define the MERs by first describing the basic principle in the main text and followed by describing the technical steps in details in the Methods section, preferably using a dedicated subsection.

Finally, the last statement in Introduction: "Our approach provides systems-level understandings of the roles of ..." may not be justified. Such a statement implies mechanistical insights; however this study only identifies markers but does not go further to investigate the underlying mechanism.

Reviewer #5:

The manuscript "Spatially guided metabolomics profiling of heterogeneous human tumor tissues" describes the development of a spatial metabolomics approach for analysing human samples, and their application to tumours of the liver.

As requested by the editorial team, this is not a full review of the manuscript. I am specifically addressing whether two issues raised by reviewer 3 have been adequately addressed in revision;

1. The definition of metabolic regions (MERs)
2. The validity of using certain non-standard markers for MER identification

Authors state that MERs are based on metabolic features following training with supervised learning approaches, using histopathology data in response to reviewer 3 point 2. The use of labels in this training would appear to confirm the reviewers point that the model is influenced by the histopathology classifications. This observation does not preclude the reported observations about clustering, and indeed this example illustrates the value added by this approach, but without repeating the training without the histopathology labels the authors cannot state that MERs are dependent solely on the MSI data. If this cannot be done, the text must more clearly describe how different inputs into the training may influence the interpretation and model responses.

The explanations of non-standard metabolites are clear and make sense. The difference between the authors response and text edits is striking (which describe the observation of Laudanosine as being not surprising and surprising respectively) but not in itself problematic. Authors clearly indicate that these treatment associated metabolites are not used as biomarkers.

Specific Responses to Reviewer #4's Comments (major changes in red):

This is not a full review of the manuscript, but rather a specific evaluation of whether the revised manuscript has adequately responded two original reviewers' (Reviewer #2 and #3) comments as requested by the editor.

The issues raised by Reviewer #2 can be roughly summarized as two parts. First, the study is largely descriptive and it is unclear what are the new findings. Second, the presentation is a bit unfocused and the figures and text should be reorganized. In this revision, the authors have done a good job improving the quality of presentation, thereby sufficiently addressing the second criticism.

On the other hand, additional revision is needed to further clarify what are the main findings. It is until the Discussion section did the authors summarize their main findings, leaving the readers in suspension while reading the majority of the manuscript. It would be helpful if the authors could also including summary statements in the Abstract and Introduction sections and, when appropriate, further provide motivating discussions in this section to guide the readers.

1. To clarify the main findings of our work, we have revised the abstract (**pg. 2**):

> Bulk high-resolution mass spectrometry provides sensitive and global snapshots of metabolites involved in cancer metabolism. However, intra-tumoral heterogeneity obscures the cellular origins of detected metabolites, making it difficult to identify reproducible and predictive metabolic markers. Here, we present "Spatially-guided MEtabolomics (SgME) profiling", a multimodal metabolomics data analysis approach that delineates and maps metabolic regions (MERs), including overlapping regions, within tumor tissues. We applied SgME profiling to human hepatocellular carcinoma (HCC) tumors and refined potential RNA markers that were also found in previous transcriptomics or bioinformatics studies to those specifically associated with malignant regions. We further estimated that more than 50% of the highly abundant metabolites detected in bulk tumors originated from the non-malignant MERs and are therefore unlikely to be predictive and/or reproducible markers. Importantly, SgME profiling also revealed new potential metabolic markers that were not apparent in bulk analysis because they increased in low-grade tumor regions but declined sharply in necrotic regions. Together, these findings show that SgME profiling overcomes key limitations of conventional metabolomics profiling by enabling more granular, spatially resolved metabolic characterization.

We have also revised the last paragraph of the Introduction section (**pg. 7**):

> In this study, we used HCC and iCCA tumors as models to evaluate and demonstrate the utility of SgME profiling. We systematically compared the transcriptomics and metabolomics landscapes across different stages of HCC and iCCA and found that early-

stage HCC exhibits the greatest intra-tumoral metabolic heterogeneity. Using SgME profiling, we trained three supervised classifiers based on metabolomic profiles to identify six different MERs in HCC tumors: metabolically (ME)-normal, -low-grade, -high-grade, -necrotic, -fibrotic, and -steatotic regions. Using COMET's Path analysis, we found many potential HCC biomarkers that had also been reported in previous large-scale transcriptomics or bioinformatics studies, assigned these potential markers to specific MERs, and narrowed them down to those associated specifically with malignant regions. We also estimated that more than 50% of the highly abundant metabolites detected in bulk tumors did not originate from malignant regions and are therefore unlikely to be predictive and/or reproducible biomarkers for HCC. Importantly, we discovered additional potential metabolic markers that were not apparent in bulk analysis because they increased in low-grade tumor regions but sharply decreased in ME-necrotic regions. Our study provides new insights into the metabolic intra-tumor heterogeneity of HCC and may help to guide future metabolomics research.

Reviewer #3 raised significant concerns regarding the definition of metabolic regions (MERs), and questioned the validity of using some of the noncommon markers for MER identification. This is an important concern, since MER-based analysis is central to this study. Unfortunately this reviewer does not have the expertise to evaluate if the explanation and additional analysis presented in this revision are sufficient to address this concern.

In addition to Reviewer #3's original comments, it appears that an exact definition of MER is missing. While a large portion of the manuscript is dedicated to the comparison of different MERs, it is unclear which properties are different because they are used to define the MERs and which are additional characteristics through integrative analysis. It would be essential for the authors to clearly define the MERs by first describing the basic principle in the main text and followed by describing the technical steps in details in the Methods section, preferably using a dedicated subsection.

2. To clearly define MER, we have added a new second paragraph to the Introduction section (**pg. 3 and 4**):

To better delineate metabolic heterogeneity in tumors, we define a "metabolic region" (MER) as a local cellular niche characterized by the spatial presence of metabolites that are mechanistically associated with key metabolic or biological processes in tumor metabolism or tumorigenesis. This definition is compatible with the concept of "metabolic zonation" in the liver (Kietzmann, 2017; Gebhardt, 1992; Jungermann & Keitzmann, 1996; Porat-Shliom, 2024), in which the spatial compartmentalization of metabolic pathways and functions enables efficient adaptation of liver metabolism to the heterogenous metabolic states and

nutritional demands of morphologically similar hepatocytes residing in different local niches. Such zonation spatially separates opposing pathways, thereby preventing competition for shared substrates, and has been described for carbohydrate, amino-acid, lipid, and xenobiotic metabolisms in both parenchymal and non-parenchymal cells (Kietzmann, 2017; Jungermann & Keitzmann, 1996). However, our definition of MER makes explicit a principle that prior definitions of zonation often leave implicit or undefined, namely the key metabolic or biological processes associated with the metabolites defining a MER may occur in the local, adjacent, or even distant cellular niches. These metabolites may be generated locally or delivered from adjacent or distant regions through transport or diffusion. Therefore, a MER represents a broader and spatially defined tissue domain that may propagate, mediate, or execute the key metabolic or biological processes in tumor metabolism, rather than being restricted to the morphologically or molecularly obvious sites where such processes are most apparent. For example, a metabolically-(ME)-necrotic region may comprise of all tissue regions containing metabolites consumed or produced during cellular necrosis, including histologically non-necrotic regions. As a corollary, this definition allows a single MER to encompass multiple cellular niche types that may have different histological or molecular profiles, while a local cellular niche may be associated with one or more MER types.

We have also revised the paragraph on PLS-DA classifiers in the Methods section (**pg. 35**):

MER definitions and PLS-DA classifiers

The MERs were fully defined by PLS-DA classifiers that were trained based on the metabolomic profiles of the initial local cellular niches (i.e., small square ROIs) annotated as described under **Histological ROI annotations**. Thus, both the mass features and histological labels of these initial local cell niches influenced the MERs identified by the classifiers. To ensure reproducibility, only 117 highly abundant putative metabolites that could be detected in >50% of all the 116 ROIs were used for training. To reduce the contributions of noise or spurious peaks, the mean abundance levels of these 117 peaks were computed for each ROIs and used for the classifier training (**Fig. EV3**). The PLS-DA classifiers were trained using the ropls package (v1.3.2) (Thévenot *et al*, 2015). For each classifier, the optimum number of predictive components (predI) was tuned to be the largest number of components before the predictive performance (Q2Y) (Wold *et al*, 2001) stopped increasing or started to decrease. The Q2Y values were estimated using a 5-fold cross validation procedure (Kohavi, 1995). The statistical significance levels of the Q2Y value were estimated by randomly permutating the labels. All the PLS-DA classifiers reported in this study have P-values < 0.05. The final trained PLS-DA classifiers were applied to the whole MSI images of

all samples, including those not used for training, to obtain the full-tissue maps for all the MERs.

Finally, we also updated the title of the manuscript to (**pg. 1**):

Spatially-guided metabolomics profiling of metabolic regions in human tumor tissues

Finally, the last statement in Introduction: "Our approach provides systems-level understandings of the roles of ..." may not be justified. Such a statement implies mechanistical insights; however this study only identifies markers but does not go further to investigate the underlying mechanism.

3. We have revised the last sentence of the Introduction to be (**pg. 7**):

Our study provides new insights into the metabolic intra-tumor heterogeneity of HCC and may help to guide future metabolomics research.

Specific Responses to Reviewer #5's Comments (major changes in red):

The manuscript "Spatially guided metabolomics profiling of heterogeneous human tumor tissues" describes the development of a spatial metabolomics approach for analysing human samples, and their application to tumours of the liver.

As requested by the editorial team, this is not a full review of the manuscript. I am specifically addressing whether two issues raised by reviewer 3 have been adequately addressed in revision;

1. The definition of metabolic regions (MERs)

2. The validity of using certain non-standard markers for MER identification

Authors state that MERs are based on metabolic features following training with supervised learning approaches, using histopathology data in response to reviewer 3 point 2. The use of labels in this training would appear to confirm the reviewers point that the model is influenced by the histopathology classifications. This observation does not preclude the reported observations about clustering, and indeed this example illustrates the value added by this approach, but without repeating the training without the histopathology labels the authors cannot state that MERs are dependent solely on the MSI data. If this cannot be done, the text must more clearly describe how different inputs into the training may influence the interpretation and model responses.

4. To make it clear that both mass and histological features can influence the MER classifiers, we have revised the description of the classifiers under the Methods section (**pg. 35**):

> **MER definitions and PLS-DA classifiers**
> The MERs were fully defined by PLS-DA classifiers that were trained based on the metabolomic profiles of the initial local cellular niches (i.e., small square ROIs) annotated as described under **Histological ROI annotations**. Thus, both the mass features and histological labels of these initial local cell niches influenced the MERs identified by the classifiers. To ensure reproducibility, only 117 highly abundant putative metabolites that could be detected in >50% of all the 116 ROIs were used for training.

We have revised the Discussion to make it clearer on the limitations of histological labels and the interpretations of the predicted regions, and provided some suggestions on future improvements (**pg. 25**):

> Our approach is general and may be applied to study other cancer types and/or metabolic diseases. Our usage of known histopathological features to identify and label ROIs for classifier training is simple and interpretable, and the identified SgME maps can be easily checked or validated based on molecular pathways known to be associated with these histopathological regions. However, such histopathological-based labelling of the training tissue regions may not fully capture the molecular complexity of MERs and thus may either completely miss a MER type (which may have unknown histology) or partially miss cellular niches with different histological features. To discover novel MERs or fully elucidate MERs, our approach may be further extended by defining ROIs for classifier training based on molecular profiles of key cancer metabolism or tumorigenesis processes (such as RNA or protein expressions or a combination of them).

The explanations of non-standard metabolites are clear and make sense. The difference between the authors response and text edits is striking (which describe the observation of Laudanosine as being not surprising and surprising respectively) but not in itself problematic. Authors clearly indicate that these treatment associated metabolites are not used as biomarkers.

12th Mar 2026
Manuscript number: MSB-2025-13233RR
Title: Spatially-guided metabolomics profiling of metabolic regions in human tumor tissues

Dear Dr Loo,

Thank you again for sending us your revised manuscript. We are now satisfied with the modifications made and I am pleased to inform you that your paper has been accepted for publication.

Your manuscript will be processed for publication by EMBO Press. It will be copy edited and you will receive page proofs prior to publication. Please note that you will be contacted by Springer Nature Author Services to complete licensing and payment information.

You may qualify for financial assistance for your publication charges - either via a Springer Nature fully open access agreement or an EMBO initiative. Check your eligibility: https://link.springer.com/journal/44320/how-to-publish-with-us

Should you be planning a Press Release on your article, please get in contact with embo_production@springernature.com as early as possible in order to coordinate publication and release dates.

If you have any questions, please do not hesitate to contact the Editorial Office. Thank you for your contribution to Molecular Systems Biology.

Sincerely,
Jingyi

Jingyi Hou, PhD
Senior Editor
Molecular Systems Biology

>>> Please note that it is Molecular Systems Biology policy for the transcript of the editorial process (containing referee reports and your response letter) to be published as an online supplement to each paper. If you do NOT want this, you will need to inform the Editorial Office via email immediately. More information is available here: https://link.springer.com/partners/embo-press/editorial-policies#Peer%20review

EMBO Press Author Checklist

Corresponding Author Name: Yulan Wang, Lit-Hsin Loo, Pierce Kah-Hoe Chow	
Journal Submitted to: Molecular Systems Biology	
Manuscript Number: MSB-2025-1323	

USEFUL LINKS FOR COMPLETING THIS FORM
The EMBO Journal - Author Guidelines
EMBO Reports - Author Guidelines
Molecular Systems Biology - Author Guidelines
EMBO Molecular Medicine - Author Guidelines

Reporting Checklist for Life Science Articles (updated January
This checklist is adapted from Materials Design Analysis Reporting (MDAR) Checklist for Authors. MDAR establishes a minimum set of requirements in transparent reporting in the life sciences (see Statement of Task: 10.31222/osf.io/9sm4x). Please follow the journal's guidelines in preparing your
Please note that a copy of this checklist will be published alongside your article.

Abridged guidelines for figures
1. Data
The data shown in figures should satisfy the following conditions:
- the data were obtained and processed according to the field's best practice and are presented to reflect the results of the experiments in an accurate and unbiased manner.
- ideally, figure panels should include only measurements that are directly comparable to each other and obtained with the same assay.
- plots include clearly labeled error bars for independent experiments and sample sizes. Unless justified, error bars should not be shown for technical
- if n<5, the individual data points from each experiment should be plotted. Any statistical test employed should be justified.
- Source Data should be included to report the data underlying figures according to the guidelines set out in the authorship guidelines on Data

2. Captions
Each figure caption should contain the following information, for each panel where they are relevant:
- a specification of the experimental system investigated (eg cell line, species name).
- the assay(s) and method(s) used to carry out the reported observations and measurements.
- an explicit mention of the biological and chemical entity(ies) that are being measured.
- an explicit mention of the biological and chemical entity(ies) that are altered/varied/perturbed in a controlled manner.
- the exact sample size (n) for each experimental group/condition, given as a number, not a range;
- a description of the sample collection allowing the reader to understand whether the samples represent technical or biological replicates (including how many animals, litters, cultures, etc.).
- a statement of how many times the experiment shown was independently replicated in the laboratory.
- definitions of statistical methods and measures:
  - common tests, such as t-test (please specify whether paired vs. unpaired), simple χ2 tests, Wilcoxon and Mann-Whitney tests, can be unambiguously identified by name only, but more complex techniques should be described in the methods section;
  - are tests one-sided or two-sided?
  - are there adjustments for multiple comparisons?
  - exact statistical test results, e.g., P values = x but not P values < x;
  - definition of 'center values' as median or average;
  - definition of error bars as s.d. or s.e.m.

Please complete ALL of the questions below.
Select "Not Applicable" only when the requested information is not relevant for your study.

Materials

**Newly Created Materials**	**Information included in the manuscript?**	**In which section is the information available?** (Reagents and Tools Table, Materials and Methods, Figures, Data Availability Section)
New materials and reagents need to be available; do any restrictions apply?	Not Applicable	

**Antibodies**	**Information included in the manuscript?**	**In which section is the information available?** (Reagents and Tools Table, Materials and Methods, Figures, Data Availability Section)
For **antibodies** provide the following information:<br>- Commercial antibodies: RRID (if possible) or supplier name, catalogue number and or/clone number<br>- Non-commercial: RRID or citation	Not Applicable	

**DNA and RNA sequences**	**Information included in the manuscript?**	**In which section is the information available?** (Reagents and Tools Table, Materials and Methods, Figures, Data Availability Section)
**Short novel DNA or RNA including primers, probes**: provide the sequences.	Not Applicable	

**Cell materials**	**Information included in the manuscript?**	**In which section is the information available?** (Reagents and Tools Table, Materials and Methods, Figures, Data Availability Section)
**Cell lines:** Provide species information, strain. Provide accession number in repository **OR** supplier name, catalog number, clone number, and/**OR** RRID.	Not Applicable	
**Primary cultures:** Provide species, strain, sex of origin, genetic modification status.	Not Applicable	
Report if the cell lines were recently **authenticated** (e.g., by STR profiling) and tested for mycoplasma contamination.	Not Applicable	

**Experimental animals**	**Information included in the manuscript?**	**In which section is the information available?** (Reagents and Tools Table, Materials and Methods, Figures, Data Availability Section)
**Laboratory animals or Model organisms:** Provide species, strain, sex, age, genetic modification status. Provide accession number in repository **OR** supplier name, catalog number, clone number, **OR** RRID.	Not Applicable	
**Animal observed in or captured from the field:** Provide species, sex, and age where possible.	Not Applicable	
Please detail **housing and husbandry conditions**.	Not Applicable	

**Plants and microbes**	**Information included in the manuscript?**	**In which section is the information available?** (Reagents and Tools Table, Materials and Methods, Figures, Data Availability Section)
**Plants:** provide species and strain, ecotype and cultivar where relevant, unique accession number if available, and source (including location for collected wild specimens).	Not Applicable	
**Microbes:** provide species and strain, unique accession number if available, and source.	Not Applicable	

**Human research participants**	**Information included in the manuscript?**	**In which section is the information available?** (Reagents and Tools Table, Materials and Methods, Figures, Data Availability Section)
If collected and within the bounds of privacy constraints report on age, sex and gender or ethnicity for all study participants.	Yes	Appendix

**Core facilities**	**Information included in the manuscript?**	**In which section is the information available?** (Reagents and Tools Table, Materials and Methods, Figures, Data Availability Section)
If your work benefited from core facilities, was their service mentioned in the acknowledgments section?	Not Applicable	

Design

**Study protocol**	**Information included in the manuscript?**	**In which section is the information available?** (Reagents and Tools Table, Materials and Methods, Figures, Data Availability Section)
If study protocol has been **pre-registered, provide DOI in the manuscript**. For clinical trials, provide the trial registration number **OR** cite DOI.	Yes	Methods
Report the **clinical trial registration number** (at ClinicalTrials.gov or equivalent), where applicable.	Yes	Methods

**Laboratory protocol**	**Information included in the manuscript?**	**In which section is the information available?** (Reagents and Tools Table, Materials and Methods, Figures, Data Availability Section)
Provide DOI OR other citation details if **external detailed step-by-step protocols** are available.	Yes	Methods

**Experimental study design and statistics**	**Information included in the manuscript?**	**In which section is the information available?** (Reagents and Tools Table, Materials and Methods, Figures, Data Availability Section)
Include a statement about **sample size** estimate even if no statistical methods were used.	Yes	Methods
Were any steps taken to minimize the effects of subjective bias when allocating animals/samples to treatment (e.g. **randomization procedure**)? If yes, have they been described?	Not Applicable	
Include a statement about **blinding** even if no blinding was done.	Not Applicable	
Describe **inclusion/exclusion criteria** if samples or animals were excluded from the analysis. Were the criteria pre-established?<br><br>If sample or data points were omitted from analysis, report if this was due to attrition or intentional exclusion and provide justification.	Yes	Methods
For every figure, are **statistical tests** justified as appropriate? Do the data meet the assumptions of the tests (e.g., normal distribution)? Describe any methods used to assess it. Is there an estimate of variation within each group of data? Is the variance similar between the groups that are being statistically compared?	Yes	Methods and Figure legends

**Sample definition and in-laboratory replication**	**Information included in the manuscript?**	**In which section is the information available?** (Reagents and Tools Table, Materials and Methods, Figures, Data Availability Section)
In the figure legends: state number of times the experiment was **replicated** in laboratory.	Yes	Figure legends
In the figure legends: define whether data describe **technical or biological replicates**.	Yes	Figure legends

Ethics

**Ethics**	**Information included in the manuscript?**	**In which section is the information available?** (Reagents and Tools Table, Materials and Methods, Figures, Data Availability Section)
Studies involving **human participants**: State details of **authority granting ethics approval** (IRB or equivalent committee(s), provide reference number for approval.	Yes	Methods
Studies involving **human participants**: Include a statement confirming that **informed consent** was obtained from all subjects and that the experiments conformed to the principles set out in the WMA Declaration of Helsinki and the Department of Health and Human Services Belmont Report.	Yes	Methods
Studies involving **human participants:** For publication of **patient photos**, include a statement confirming that consent to publish was obtained.	Not Applicable	
Studies involving experimental **animals**: State details of **authority granting ethics approva**l (IRB or equivalent committee(s), provide reference number for approval. Include a statement of compliance with ethical regulations.	Not Applicable	
Studies involving **specimen and field samples:** State if relevant **permits** obtained, provide details of authority approving study; if none were required, explain why.	Yes	Methods

**Dual Use Research of Concern (DURC)**	**Information included in the manuscript?**	**In which section is the information available?** (Reagents and Tools Table, Materials and Methods, Figures, Data Availability Section)
Could your study fall under dual use research restrictions? Please check biosecurity documents and list of **select agents and toxins** (CDC): https://www.selectagents.gov/sat/list.htm	Not Applicable	
If you used a select agent, is the security level of the lab appropriate and reported in the manuscript?	Not Applicable	
If a study is subject to dual use research of concern regulations, is the name of the **authority granting approval and reference number** for the regulatory approval provided in the manuscript?	Not Applicable	

Reporting

The MDAR framework recommends adoption of discipline-specific guidelines, established and endorsed through community initiatives. Journals have their own policy about requiring specific guidelines and recommendations to complement MDAR.

**Adherence to community standards**	**Information included in the manuscript?**	**In which section is the information available?** (Reagents and Tools Table, Materials and Methods, Figures, Data Availability Section)
State if relevant guidelines or checklists (e.g., **ICMJE, MIBBI, ARRIVE, PRISMA**) have been followed or provided.	Not Applicable	
For **tumor marker prognostic studies,** we recommend that you follow the **REMARK** reporting guidelines (see link list at top right). See author guidelines, under 'Reporting Guidelines'. Please confirm you have followed these guidelines.	Not Applicable	
For **phase II and III randomized controlled trials**, please refer to the **CONSORT** flow diagram (see link list at top right) and submit the CONSORT checklist (see link list at top right) with your submission. See author guidelines, under 'Reporting Guidelines'. Please confirm you have submitted this list.	Not Applicable	

Data Availability

**Data availability**	**Information included in the manuscript?**	**In which section is the information available?** (Reagents and Tools Table, Materials and Methods, Figures, Data Availability Section)
Have **primary datasets** been deposited according to the journal's guidelines (see 'Data Deposition' section) and the respective accession numbers provided in the Data Availability Section?	Yes	Data availability section
Were **human clinical and genomic datasets** deposited in a public access-controlled repository in accordance to ethical obligations to the patients and to the applicable consent agreement?	Not Applicable	
Are **computational models** that are central and integral to a study available without restrictions in a machine-readable form? Were the relevant accession numbers or links provided?	Yes	Data availability section (can be downloaded from GitHub)
If publicly available data were reused, provide the respective **data citations in the reference list**.	Yes	Reference
